# Micronutrients or processing? An analysis of food and drink items from the UK National Diet and Nutrition Survey based on micronutrient content, the Nova classification and front-of-package traffic light labelling

**DOI:** 10.1017/S0007114524003374

**Published:** 2025-02-14

**Authors:** Samuel J. Dicken, Rachel L. Batterham, Adrian Brown

**Affiliations:** 1 Centre for Obesity Research, Department of Medicine, University College London (UCL), London WC1E 6JF, UK; 2 Department of Behavioural Science and Health, University College London (UCL), London WC1E 7HB, UK; 3 National Institute for Health Research, Biomedical Research Centre, University College London Hospital (UCLH), London W1T 7DN, UK; 4 Bariatric Centre for Weight Management and Metabolic Surgery, University College London Hospital (UCLH), London NW1 2BU, UK

**Keywords:** Front-of-package labelling, Ultra-processed food, NOVA classification, Micronutrients, Dietary guidelines, Diet recommendations

## Abstract

Increased ultra-processed food (UPF) is associated with adverse health outcomes. However, with limitations in UPF evidence, and partial overlap between UK front-of-package labelling (FOPL) and degree of food processing, the value of food processing within dietary guidance is unclear. This study compared food and drink from the UK National Diet and Nutrition Survey (NDNS) database based on micronutrient content, Nova classification and FOPL. The aim was to examine the micronutrient contributions of UK food and drink to UK government dietary micronutrient recommendations for adult females and males, aged 19–64 years, based on the degree of food processing and FOPL. NDNS items were coded into minimally processed food (MPF), processed culinary ingredients, processed food (PF) and UPF, and FOPL traffic lights. MPF, PF and UPF provided similar average contributions per 100 g to micronutrient recommendations. Per 100 kcal, MPF provided the greatest average contribution (14·4 % (interquartile range (IQR): 8·2–28·1)), followed by PF (7·7 % (IQR: 4·6–10·9) and then UPF (5·8 % (IQR: 3·1–9·7)). After adjusting for healthy/unhealthy items (presence of 1+ red FOPL), MPF had higher odds of an above-average micronutrient contribution per 100 kcal than UPF (OR: 5·9 (95 % CI 4·9–7·2)) and PF (OR: 3·2 (95 % CI 2·4–4·2)). MPF were more likely to provide greater contributions to micronutrient recommendations than PF or UPF per 100 kcal. These findings suggest that UPF or PF diets are less likely to meet micronutrient recommendations than an energy-matched MPF diet. The results are important for understanding how consumers perceive the healthiness of products based on FOPL.

For centuries, food processing has provided people around the world with a safe and long-lasting food supply^(1)^. However, recent evidence suggests the degree of food processing may also be an important dietary determinant of health^(2,3)^. Most commonly defined by the Nova classification^(4)^, ultra-processed foods (UPF) are industrially formulated, typically with five or more ingredients, resulting in highly palatable, long-lasting, readily accessible and more affordable products^(5)^. The Nova classification also categorises foods and drinks into three other groups with increasing degrees of processing: minimally processed food (MPF), processed culinary ingredients (PCI) and processed food (PF).

Prospective studies suggest increased risks of several non-communicable diseases with increasing UPF intake, generally with low or very-low quality of evidence^(3)^. However, scientific debate continues regarding the role of food processing alongside existing dietary guidance, such as those in the UK^(6,7)^. Ultra-processing may impact on health beyond nutrient content or dietary pattern^(2)^, such as from additives or neo-formed contaminants^(8)^, and influence dietary behaviours through changes in food texture, palatability, portion size, energy density and eating rate^(9,10)^. However, the exact mechanisms are yet to be confirmed.

Dietary advice in the UK is provided to the public via the Eatwell Guide (EWG)^(11)^. The EWG is communicated in the retail environment through multiple traffic light (MTL) front-of-package labels (FOPL)^(11,12)^, which assigns a green, amber or red FOPL colour based on low, medium or high fat, saturated fat, salt or sugar content, respectively^(12)^. A previous meta-analysis reported that diets higher in UPF tend to be higher in fat, saturated fat and free sugar, and lower in fibre, protein and micronutrients including potassium, Zn, Mg and vitamins A, C, D, E, B_12_ and niacin^(13)^. However, assessment of UPF intake with dietary recalls, often limited to a single day^(13)^, may not fairly represent and reflect the wide range of foods and drink within each food processing group, or consumed by an individual over time. To do so requires assessment of a national food/drink and nutrient database. A recent analysis of the nationally representative UK National Diet and Nutrition Survey (NDNS) nutrient database demonstrated partial overlap between macronutrient content, MTL FOPL and the degree of food processing^(14)^. UPF tended to have an unhealthier nutrient profile and were more likely to have a worse MTL FOPL score, for example, higher in fat, saturated fat, salt, sugar and/or energy, but some UPF were considered healthy according to their macronutrient profile and MTL FOPL, which aligned with previously reported data^(5)^. However, no study has comprehensively assessed the micronutrient content of a representative supply of food and drink in the UK based on FOPL MTL and Nova classification, and their relative contributions to governmental dietary recommendations^(11)^.

The aim of this study was to assess the micronutrient content of the NDNS food/drink database; first, to determine the contributions of food and drink to recommended micronutrient intakes overall and across processing groups according to Nova; second, whether contributions differed according to FOPL MTL; and third, whether contributions differed according to Nova, independent of FOPL MTL.

## Methods

### Data sources

Details on the methods used have been reported elsewhere^(14)^. Briefly, NDNS is a repeated cross-sectional survey, that since 2008 provides comprehensive assessment of dietary intake from a nationally representative UK sample living in private households and aged 1·5 years and older^(15)^. From 2008 to 2019, the survey was conducted using 4-d food diaries completed across consecutive days. In 2019/20, 4-d food diaries were replaced with four non-consecutive, multiple-pass, 24-h dietary recalls using Intake24^(16)^. Intake24 is an online, automated, self-reported 24-h dietary recall (https://intake24.co.uk)^(17)^. This analysis used nutrient composition data of food and drink items in the Year 12 survey and reported nutrient intakes from participant food diaries from the Year 9–11 survey (2016/17–2018/19). Food and drink names and subgroups were obtained via the Intake24 team. The matching nutrient databank from NDNS with the latest publicly available data on the nutrient content for each item was obtained from the UK Data Service (https://beta.ukdataservice.ac.uk), as well as the latest publicly available data on reported nutrient intakes from Years 9–11. Nutrient content in the nutrient databank was determined from multiple sources, primarily, from the UK Composition of Foods Integrated Dataset^(18)^. This is supplemented with manufacturer data from food labels and the web, and from the Food Standards Agency (FSA) Food Recipes Database^(19)^, and manufacturers’ data gathered through food labels and web information. Nutrient values are assigned to all foods, ensuring no missing values. For nutrients without reliable information, values are extrapolated from similar foods. Finally, all nutrient values are inspects before being added to the databank^(20)^. Detail on Intake24 and NDNS have been previously published^(16)^.

### Nova classification

Coding of food and drink items into the Nova classification (see online Supplementary Materials for definitions)^(4)^ has been described in detail elsewhere^(14)^. Each item in the Year 12 NDNS dataset was individually coded and conducted with authors blind to the nutrient database. Classification was determined based on definitions of each processing group in the Nova classification^(4)^, item name, subgroup code, representative products from UK supermarkets and the assigned Nova group of the corresponding item in the NDNS database from Years 1–11^(21)^. Of the 3105 items in the database, 109 items were coded outside of the NOVA classification (e.g. fish oil supplements and multivitamins) and were removed before analysis as in previous publications^(14)^. An additional sixteen items had no corresponding item in the NDNS nutrient databank. Of the 2980 remaining items, 55·4 % were UPF (*n* 1650), 33·1 % were MPF (*n* 986), 9·5 % were PF (*n* 283) and 2·0 % (*n* 61) were PCI.

### Front-of-package labelling classification

Coding of food and drink items into MTL FOPL according to the Department of Health and Food Standards Agency guidance for fat, saturated fat, total sugar and salt content^(12)^ has been also been described in detail elsewhere^(14)^. For each nutrient, items with low content are coded green, with moderate content as amber and with high content as red (thresholds are outlined in online Supplementary Table 1). For comparability, items were coded per 100 g. Drinks were coded using the lower threshold guidance for amber or red coding per 100 g, which was assumed equivalent to 100 ml of drink.

### Micronutrient content

The NDNS nutrient databank includes data on micronutrient content (vitamins and minerals) per 100 g for foods and drinks, including retinol, total carotene, *α*-carotene, *β*-carotene, *β*-cryptoxanthin, vitamins A (retinol equivalents), D, C, E, B_6_, and B_12_, thiamin, riboflavin, niacin equivalent, folate, pantothenic acid, biotin, potassium, Ca, Mg, phosphorus, Fe, haem iron, non-haem iron, Cu, Zn, Na, chloride (Cl), iodine, Mn and Se. Micronutrient content was also calculated per 100 kcal by dividing the micronutrient value per 100 g for each item by its energy content per 100 g and then multiplying by 100. A total of twenty-five items contained zero energy content per 100 g (i.e. water, weak teas, salts and no-calorie sweeteners) and were removed from the per 100 kcal analysis.

Percent contributions to meeting daily reference nutrient intakes (RNI) (the quantity considered to meet 97·5 % of the population’s requirements) for key micronutrients were calculated from the UK government dietary recommendations for females and males aged 19–64 years in the general population, respectively^(11)^. These recommendations are based on the Committee on Medical Aspects of Food Policy (COMA) and the Scientific Advisory Committee on Nutrition (SACN) reports on Dietary Reference Values^(22–24)^. Micronutrient dietary recommendations are provided for: vitamin A (retinol equivalents), D, C, E, B_6_, B_12_, thiamin, riboflavin, niacin equivalent, folate, potassium, Ca, Mg, phosphorus, Fe, Cu, Zn, Na, chloride, iodine and Se (online Supplementary Table 2). For females, 14·8 mg was used for Fe, as the daily recommendation for 19–49 year olds (with 8·7 mg recommended for females aged 50–64 years)^(11)^. Percent contributions to meeting government daily micronutrient intakes were reported per 100 g and per 100 kcal of each food and drink. This represents the quantity of each micronutrient per 100 g and per 100 kcal from compositional data, as a proportion of the recommended intake. Two UPF items were missing data on Se content: low-protein pasta (e.g. Loprofin) and crunchy, cluster-type cereal (e.g. Kellogg’s/Nestle) – they were included in analyses for other micronutrients but excluded from overall micronutrient calculations.

To calculate overall average percent contributions to government dietary micronutrient recommendations for males and females, the percent contribution per 100 g and 100 kcal of each food or drink to each micronutrient recommendation was averaged (i.e. the percentage values for each individual micronutrient were averaged to provide a composite micronutrient value, per 100 g and per 100 kcal). Na and chloride were excluded from the overall average as Na is a nutrient to limit, and consumption of chloride as it is most commonly consumed as sodium chloride. Vitamin E was excluded from overall contributions as it is reported in NHS guidance^(25)^, but not in UK government guidance^(11)^.

For comparison to actual UK adult intakes, the average percent contributions to government dietary micronutrient recommendations from micronutrient intakes obtained from participant food diaries from the NDNS Year 9–11 survey for males and females, aged 19–64, were reported, as well as the percent contributions per 100 kcal of reported total daily energy intake. Total diet weight was not available in the publicly available NDNS dataset.

### Statistical analysis

Given the sex-specific government micronutrient recommendations, analyses were conducted separately for males and females. Analysis by weight (100 g) was conducted as the unit used for creating MTL FOPL. However, this does not reflect energy content. Analysis by energy content (100 kcal) was conducted, given the evidence linking UPF with increased energy intake and the importance of energy intake for weight management and obesity-related non-communicable disease^(24)^. Non-parametrically distributed variables were described using medians and interquartile ranges (IQR), and categorical variables using counts and percentages. Comparisons of non-parametrically distributed micronutrient variables between Nova groups were analysed using Kruskal–Wallis ANOVA. Categorical variables were analysed using *χ* ^2^ tests. Comparisons of two non-parametrically distributed micronutrient variables (healthy and unhealthy foods within each Nova group (e.g. UPF with or without a red FOPL traffic light)) were analysed using Mann–Whitney *U* tests. Bonferroni correction was used for multiple comparisons.

The average micronutrient content and percent contributions to government micronutrient recommendations per 100 g and per 100 kcal were described for all food and drinks, and for each Nova group, for males and females, separately. The proportion of items with zero content for each micronutrient were also reported overall, and for each Nova group.

As in a previous analysis of the UK NDNS and Nova classification^(14)^, items were classified into ‘healthy’ or ‘unhealthy’ based on the presence or absence of a red FOPL traffic light for fat, saturated fat, total sugar or salt. This is based on research that when identifying healthier products, UK consumers are more cautious to avoid items with red traffic lights, than to select items with green traffic lights^(26,27)^. Subgroup analyses were then used to compare the average micronutrient content and percent contributions to government dietary recommendations of ‘healthy’ *v*. ‘unhealthy’ food and drink items per 100 g and per 100 kcal across the NDNS database, for each Nova group (i.e. healthy *v*. unhealthy UPF), and then between NOVA food groups within healthy’ *v*. ‘unhealthy’ subgroups (i.e. comparing the micronutrient content of healthy products across Nova groups). The number of items across quartiles of average percentage contributions to daily government micronutrient recommendations of food and drink items per 100 g and per 100 kcal were then compared by Nova group and by healthy/unhealthy FOPL score.

Regression analysis was then used to examine the relationship between NOVA group and micronutrient content, accounting for MTL FOPL score. Binary regression was used to model the odds of a food or drink item containing above-average percent contribution to government micronutrient recommendations per 100 g and per 100 kcal (i.e. an above median *v*. median or below percent contribution to government dietary recommendations), with Nova group (categorical: MPF, PCI, PF and UPF) and FOPL score (categorical: healthy *v*. unhealthy) as explanatory variables.

### Sensitivity analysis

Overall average percent contributions to government micronutrient recommendations of each food or drink per 100 g and 100 kcal for each micronutrient was repeated including Na and chloride into the overall estimate. Statistical significance was set at < 0·05. Data analysis was conducted in SPSS V29.0, and R version 2024.04.1 + 748.

## Results

### Contributions of food and drink to recommended micronutrient intakes overall and by Nova group

**Figure 1.**
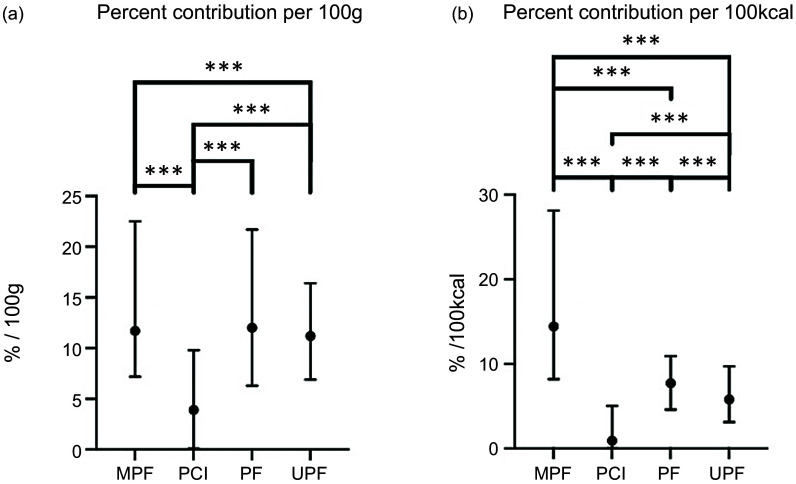
Average percentage contribution to meeting UK government micronutrient recommendations from food and drink items in the UK National Diet and Nutrition Survey for females aged 19–64 years across each Nova group: (a) all items per 100 g and (b) all items per 100 kcal. ***denotes significance at *P* < 0·001, ** denotes significance *P* < 0·01 conducted from Kruskal–Wallis ANOVA with Bonferroni correction for multiple comparisons. MPF, minimally processed food; PCI, processed culinary ingredient; PF, processed food; UPF, ultra-processed food.

Given the similarities in results between females and males, results for females are presented in the main tables and males in the online Supplementary Materials. The average percent contributions to government dietary micronutrient recommendations from micronutrient intakes obtained from participant food diaries from the NDNS Year 9–11 survey for males and females, aged 19–64, are reported in online Supplementary Table 2, including the percentage of adults meeting the government dietary micronutrient recommendations. From the food diaries in NDNS Year 9–11, on average, females met 10 (IQR: 7–13), and males, 12 (IQR: 8–15) out of eighteen micronutrient recommendations. From the nutrient database for the 2980 food and drink items, average percentage contributions to daily micronutrient recommendations of each key vitamin and mineral per 100 g for females aged 19–64 years across each Nova group are reported in Fig. 1 and online Supplementary Table 3. The nutrient content of food and drink items from the nutrient database on average would provide 11·3 % (IQR: 6·8–18·0) to each female dietary micronutrient recommendation per 100 g. MPF would contribute on average 11·7 % (IQR: 7·2–22·5), PF 12·0 % (IQR: 6·3–12·7), UPF 11·2 % (IQR: 6·9–16·4) and PCI 3·9 % (IQR: 0·1–9·8). The distributions of MPF and UPF contributions significantly differed (*P* < 0·001), but PF did not significantly differ from MPF and UPF contributions. The distributions of MPF, PF and UPF contributions did not significantly differ when including Na and Cl into average percentage contributions. Average absolute micronutrient content per 100 g is reported in online Supplementary Table 4, including micronutrients without governmental recommendations. Findings were similar for percentage contributions to daily dietary micronutrient recommendations for males aged 19–64 years per 100 g (online Supplementary Table 5).

Table 1 reports the average percentage contribution to daily recommendations of each key micronutrient per 100 kcal of the 2955 food and drink items for females aged 19–64 years and across each Nova group. Table 1 also reports percent contributions per 100 kcal of reported energy intake from food diaries of females, aged 19–64 years from the NDNS Year 9–11 survey. The average absolute micronutrient content per 100 kcal is reported in online Supplementary Table 6, including micronutrients without governmental recommendations. Food and drink items on average would contribute 7·8 % (IQR: 4·1–14·4) to each female dietary micronutrient recommendation per 100 kcal. MPF would contribute on average 14·4 % (IQR: 8·2–28·1), PF 7·7 % (IQR: 4·6–10·9), UPF 5·8 % (IQR: 3·1–9·7) and PCI 0·9 % (IQR: 0·0–5·0). MPF, PCI, PF and UPF contributions all significantly differed. Including Na and Cl into average percentage contributions did not alter findings. Findings were similar for percentage contributions to daily dietary micronutrient recommendations for males aged 19–64 years per 100 kcal (online Supplementary Table 7, including percent contributions per 100 kcal of reported energy intake from food diaries of males, aged 19–64 from the NDNS Year 9–11 survey). Online Supplementary Fig. 1 shows the percentage contributions to UK government micronutrient recommendations for females aged 19–64 years for each micronutrient by Nova group, per 100 g (1a), and per 100 kcal (1b).

**Table 1.** Average percentage contribution per 100 kcal to UK government dietary micronutrient recommendations from the UK National Diet and Nutrition Survey for females aged 19–64 years, and across each Nova group, and percentage of government dietary micronutrient recommendations consumed per 100 kcal of reported energy intake for females, aged 19–64 years from the national diet and nutrition survey Year 9–11 survey (Median values and interquartile ranges)

	Total (*n* 2955)		MPF (*n* 978)		PCI (*n* 57)		PF (*n* 283)		UPF (*n* 1637)		Number of items with 0 content of micronutrient per 100 kcal	Percentage of RNI consumed per 100 kcal of reported energy intake from NDNS years 9–11	
Micronutrient	Median	IQR	Median	IQR	Median	IQR	Median	IQR	Median	IQR		Median (%)	IQR
Vitamins												7·1	
Vitamin A (retinol equivalents) (%)	1·4	0–8·3	2·4 (a)	0·0–15·9	0·0 (b)	0·0–20·8	4·5 (a)	0·5–10·7	0·9 (b)	0·0–5·3	949		4·6–11·1
Vitamin D (%)	0·0	0–1·242	0·0 (a)	0·0–0·9	0·0 (a)	0·0–0·5	0·7 (b)	0·0–2·5	0·0 (c)	0·0–1·2	1733	1·4	0·8–2·2
Thiamin (%)	7·1	2·9–15·4	12·1 (a)	6·6–25·0	0·0 (b)	0·0–0·7	4·1 (c)	1·6–8·0	5·6 (d)	2·0–12·5	331	10·3	8·5–12·5
Riboflavin (%)	5·2	2·0–10·8	8·1 (a)	3·8–16·1	0·0 (b)	0·0–7·1	5·9 (c)	2·7–10·0	4·0 (d)	1·4–8·2	287	7·6	6·1–9·7
Niacin equivalent (%)	11·6	4·6–22·4	17·3 (a)	9·5–33·3	0·0 (b)	0·0–0·8	11·6 (c)	5·6–19·9	8·8 (d)	3·5–17·4	173	14·7	12·1–18·0
Vitamin C (%)	0·0	0·0–11·2	7·3 (a)	0·0–64·5	0·0 (b)	0·0–0·0	0·1 (c)	0·0–6·0	0·0 (d)	0·0–2·5	1481	11·6	7·1–17·0
Vitamin E (%)	11·2	3·3–31·0	17·3 (a)	4·6–47·6	6·5 (b)	0·0–18·9	10·7 (b)	4·0–27·1	10·5 (b)	2·8–24·7	459		
Vitamin B_6_ (%)	5·7	0·0–13·0	11·9 (a)	6·2–24·9	0·0 (b)	0·0–0·0	4·6 (c)	2·0–9·5	3·4 (d)	0·0–8·2	805	7·4	6·1–9·3
Vitamin B_12_ (%)	0·0	0·0–22·5	0·0 (a)	0·0–38·2	0·0 (b)	0·0–1·9	10·3 (c)	0·0–35·8	1·6 (a)	0·0–18·3	1583	16·9	12·5–23·2
Folate (%)	3·6	1·3–8·9	6·8 (a)	2·9–23·1	0·0 (b)	0·0–0·8	3·4 (c)	1·5–7·2	2·6 (d)	1·0–5·9	279	6·4	5·1–7·9
Minerals													
Potassium (%)	3·6	1·8–8·0	9·1 (a)	5·1–15·0	0·1 (b)	0·0–1·5	3·2 (c)	1·6–5·4	2·5 (d)	1·5–4·2	36	4·5	3·8–5·3
Ca (%)	3·8	1·6–9·3	4·2 (a)	1·6–14·3	0·3 (b)	0·0–6·5	4·3 (a)	2·1–10·8	3·6 (c)	1·6–7·5	31	6·5	5·3–7·6
Mg (%)	4·8	2·9–9·3	9·0 (a)	5·2–15·4	0·1 (b)	0·0–1·9	3·9 (c)	2·6–6·7	3·8 (c)	2·2–6·3	59	5·5	4·6–6·5
Phosphorus (%)	11·7	6·0–19·7	18·2 (a)	9·9–29·9	0·2 (b)	0·0–7·3	12·4 (c)	5·8–20·1	9·5 (d)	5·0–14·8	61	12·4	10·9–14·5
Fe (%)	3·4	1·8–7·3	6·6 (a)	3·0–13·5	0·1 (b)	0·0–0·9	2·7 (c)	1·4–5·3	2·8 (c)	1·6–5·2	210	4·3	3·5–6·0
Cu (%)	4·4	2·2–8·3	7·4 (a)	3·5–15·5	0·1 (b)	0·0–1·3	3·5 (c)	1·7–7·6	3·8 (c)	1·9–6·3	376	5·5	4·7–6·6
Zn (%)	5·7	2·7–11·9	10·9 (a)	5·1–19·0	0·4 (b)	0·0–2·4	6·2 (c)	2·8–11·6	4·1 (d)	2·1–8·0	266	6·6	5·6–7·9
Na (%)	4·5	1·2–10·4	2·0 (a)	0·3–5·8	0·2 (b)	0·0–1·4	6·0 (c)	2·1–11·7	6·8 (c)	2·6–12·6	85	6·9	5·8–8·2
Chloride (%)	5·0	1·6–10·9	3·5 (a)	1·3–8·2	0·1 (b)	0·0–1·4	6·1 (c)	2·7–11·0	6·1 (c)	2·0–12·5	115	7·4	6·3–8·4
Iodine (%)	2·5	0·8–6·0	3·1 (a)	1·3–7·9	0·0 (b)	0·0–1·6	4·4 (c)	1·7–8·4	2·0 (c)	0·6–4·6	493	5·6	4·3–7·4
Se (%)	2·6	0–6·5	4·4 (a)	0·7–10·5	0·0 (b)	0·0–0·0	2·4 (c)	0·0–5·9	2·2 (c)	0·7–5·0	668	4·3	3·4–5·7
Overall (%) /18–Exc VitE, NA, CL) (*n* 2953)	7·8	4·1–14·4	14·4 (a)	8·2–28·1	0·9 (b)	0·0–5·0	7·7 (c)	4·6–10·9	5·8 (d)	3·1–9·7	14	8·0	6·9–9·4
Overall (%) /20, Inc NA, CL, Exc VItE) (*n* 2953)	7·9	4·3–13·8	13·3 (a)	7·8–26·4	0·8 (b)	0·0–4·7	7·5 (c)	4·8–11·0	6·3 (d)	3·2–10·4	11	7·9	7·0–9·1

IQR, interquartile range; MPF, minimally processed food; PCI, processed culinary ingredients; PF, processed food; UPF, ultra-processed food; Exc., excluded; Inc, included; RNI, reference nutrient intake; NDNS, National Diet and Nutrition Survey.

Overall (%) (/18) includes all micronutrients except Na, chloride and vitamin E. Overall (%) (/20) includes all micronutrients except vitamin E. Unlike letters indicate significantly different *P* < 0·05. Pairwise comparisons conducted using Kruskal–Wallis ANOVA with Bonferroni correction for multiple comparisons. Se: total = 2953 (UPF, *n* 1635).

The proportions of Nova groups across quartiles of average percentage contributions to daily dietary recommendations of food and drink items significantly differed per 100 g and per 100 kcal (both *P* < 0·001) (online Supplementary Table 8). In the highest quartile of average percentage contribution to daily micronutrient recommendations per 100 g, 330 were MPF, 5 were PCI, 91 were PF and 322 were UPF. In total, 33·5 % of MPF were in the highest quartile, compared with 19·3 % of UPF, and 32·2 % of PF. In the highest quartile of average percentage contribution to daily micronutrient recommendations per 100 kcal, 489 were MPF, 7 were PCI, 43 were PF and 199 were UPF. Fifty per cent of MPF were in the highest quartile, compared with 12·2 % of UPF and 15·2 % of PF, while 6·1 % of MPF were in the lowest quartile, compared with 35·2 % of UPF and 21·6 % of PF. The proportion of healthy and unhealthy items based on FOPL significantly differed across quartiles of average percentage contributions to daily micronutrient recommendations per 100 g and per 100 kcal (both *P* < 0·001) (online Supplementary Table 8). Per 100 g, the lowest *v*. highest quartile of average percentage contribution to daily micronutrient recommendations contained a greater proportion of items with one or more red FOPL (unhealthy items; 30·4 % *v*. 50·5 %). Per 100 kcal, the lowest *v*. highest quartile of average percentage contribution to daily micronutrient recommendations contained a greater proportion of items with no red FOPL (healthy items; 27·9 % *v*. 83·5 %). Per 100 kcal, only 16·5 % of the highest quartile were unhealthy items.

### Contributions of food and drink to recommended micronutrient intakes by Nova group and front-of-package labelling multiple traffic light

**Figure 2.**
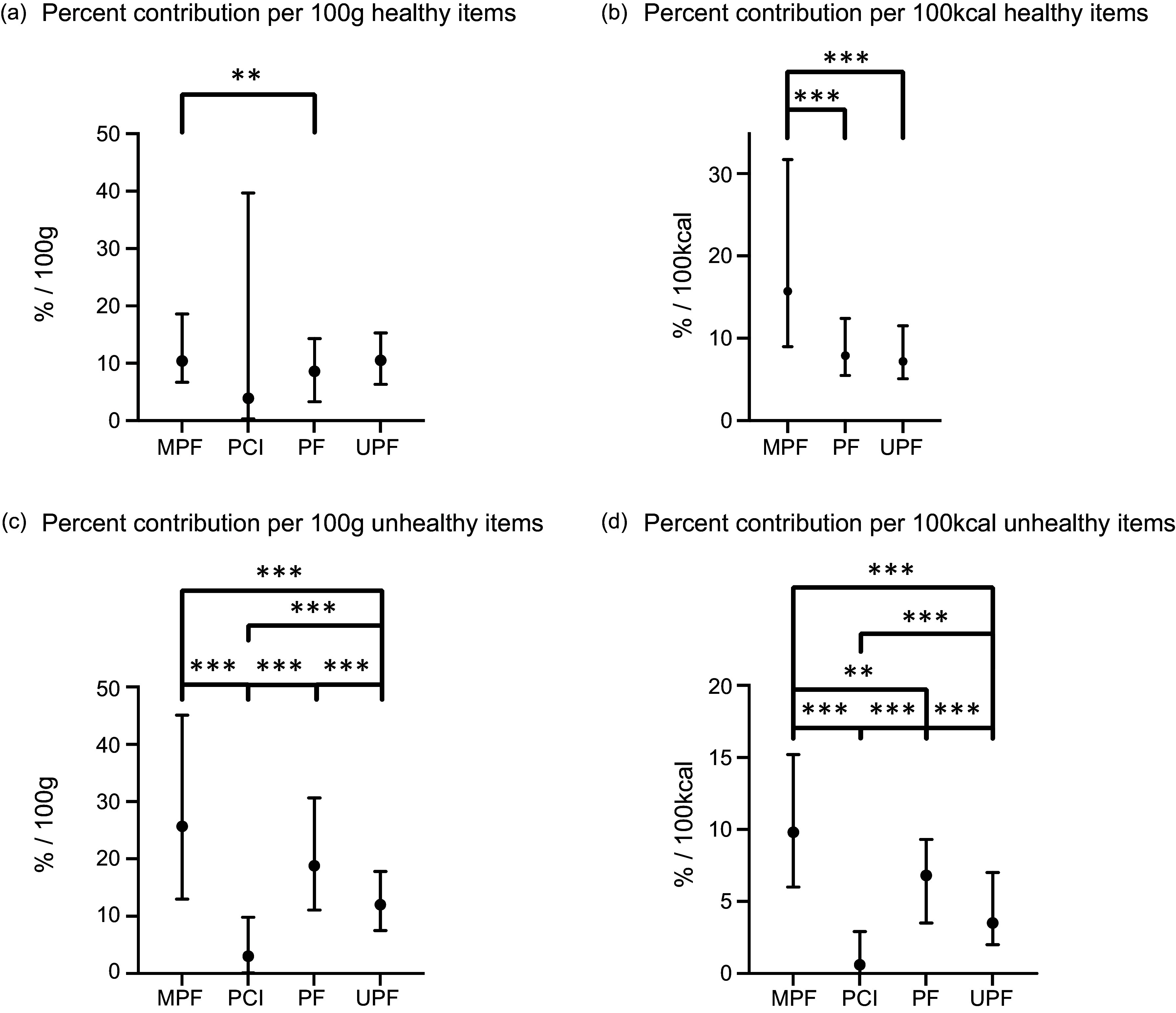
Average percentage contribution to UK government dietary micronutrient recommendations from the UK National Diet and Nutrition Survey for females aged 19–64 years across Nova groups: (a) healthy items per 100 g, (b) healthy items per 100 kcal, (c) unhealthy items per 100 g and (d) unhealthy items per 100 kcal. ***denotes significance at *P* < 0·001, ** denotes significance *P* < 0·01 conducted from Kruskal–Wallis ANOVA with Bonferroni correction for multiple comparisons. 1.d PCI omitted from the graph for clarity. Items were classified into ‘healthy’ or ‘unhealthy’ based on the presence or absence of a red FOPL traffic light for fat, saturated fat, total sugar or salt. This is based on research that when identifying healthier products, UK consumers are more cautious to avoid items with red traffic lights than to select items with green traffic lights^(26,27)^. MPF, minimally processed food; PCI: processed culinary ingredient; PF, processed food; UPF, ultra-processed food.

Average percentage contributions to daily recommendations of key micronutrients per 100 g of the 1849 healthy food and drink items for females aged 19–64 years across each Nova group are reported in Fig. 2 and online Supplementary Table 9. Healthy food and drink items on average would contribute 10·3 % (IQR: 6·3–16·4) to each female dietary micronutrient recommendation per 100 g. MPF would contribute on average 10·4 % (IQR: 6·7–18·6), PF 8·6 % (IQR: 3·3–14·3), UPF 10·5 % (IQR: 6·3–15·3) and PCI 3·9 % (IQR: 0·3–39·7) per 100 g. The distribution of MPF was similar to UPF, but PF was significantly different to MPF. Healthy UPF would provide significantly lower average micronutrient content per 100 g than healthy MPF for three micronutrients and higher average content for ten micronutrients (including Na and chloride). Compared with healthy PF, UPF would provide significantly lower average micronutrient content for two micronutrients (vitamins A and C) and significantly greater average micronutrient content for nine micronutrients.

Table 2 reports the average percentage contribution to daily recommendations of key micronutrients per 100 kcal of the 1825 healthy food and drink items for females aged 19–64 years and across each Nova group. Healthy food and drink items on average would contribute 9·9 % (IQR: 6·1, 18·8) to each female micronutrient recommendation per 100 kcal. MPF would contribute on average 15·7 % (IQR: 9·0, 31·7), PF 7·9 % (IQR: 5·5, 12·4), UPF 7·2 % (IQR: 5·1, 11·5) and PCI 132·7 % (IQR: 0·3, 386·2) per 100 kcal. The distribution of MPF was significantly higher than PF and UPF, but PF and UPF were similar. Healthy UPF would provide significantly lower average micronutrient content per 100 kcal than healthy MPF for seventeen micronutrients. Compared with healthy PF, UPF would provide significantly lower average micronutrient content for four micronutrients (vitamin A and C, potassium and iodine) and would provide significantly greater average micronutrient content for three micronutrients (thiamine, Na and chloride). Absolute content of healthy items per 100 g and per 100 kcal is reported in online Supplementary Tables 10 and 11.

**Table 2.** Average percentage contribution of healthy items per 100 kcal to UK government dietary micronutrient recommendations from the UK National Diet and Nutrition Survey for females aged 19–64 years, and across each Nova group (Median values and interquartile ranges)

Micronutrient	Total (*n* 1825)		MPF (*n* 812)		PCI (*n* 9)		PF (*n* 162)		UPF (*n* 842)		Number of items with 0 content of micronutrient per 100 kcal
	Median	IQR	Median	IQR	Median	IQR	Median	IQR	Median	IQR	
Vitamins											
Vitamin A (retinol equivalents) (%)	1·7	0·0–9·1	2·9 (a)	0·0–18·2	0·0 (b)	0·0–0·0	2·6 (a)	0·4–8·3	0·8 (c)	0·0–5·7	572
Vitamin D (%)	0·0	0·0–1·1	0·0 (a)	0·0–0·8	0·0 (a)	0·0–0·0	0·1 (b)	0·0–2·2	0·0 (b)	0·0–1·1	1118
Thiamin (%)	10·3	5·2–19·2	13·4 (a)	7·3–27·4	125·0 (abc)	0·0–146·2	5·2 (b)	2·4–9·5	9·4 (c)	4·3–15·5	174
Riboflavin (%)	6·1	2·7–12·4	8·3 (a)	4·0–17·6	215·2 (ab)	0·0–545·5	5·4 (b)	2·7–10·0	4·5 (b)	1·9–9·1	161
Niacin equivalent (%)	14·8	8·0–26·8	18·3 (a)	10·3–36·7	0·0 (b)	0·0–35·3	10·8 (b)	5·5–21·6	13·3 (b)	6·6–21·2	66
Vitamin C (%)	2·2	0·0–31·1	13·3 (a)	0·0–80·6	0·0 (b)	0·0–0·0	2·2 (c)	0·0–20·9	0·1 (b)	0·0–8·1	733
Vitamin E (%)	12·4	3·1–33·9	17·5 (a)	4·5–50·5	0·0 (b)	0·0–0·0	12·9 (c)	3·9–29·6	10·9 (c)	2·8–27·8	306
Vitamin B_6_ (%)	7·6	2·3–18·3	13·9 (a)	6·3–27·8	0·0 (b)	0·0–47·2	6·9 (b)	0·0–13·4	4·8 (b)	0·0–10·4	438
Vitamin B_12_ (%)	0·0	0·0–27·9	0·0 (a)	0·0–36·7	0·0 (a)	0·0–0·0	5·5 (b)	0·0–29·4	0·0 (ab)	0·0–24·5	1061
Folate (%)	5·1	2·6–13·7	8·2 (a)	3·3–30·2	50·0 (ab)	0·0–614·6	3·4 (b)	1·8–8·6	4·4 (b)	2·3–8·0	107
Minerals											
Potassium (%)	5·4	2·9–10·8	10·0 (a)	5·8–17·3	20·0 (ab)	0·1–28·6	4·7 (b)	2·9–6·9	3·3 (c)	2·1–5·4	13
Ca (%)	4·6	2·2–11·1	4·4 (a)	1·8–14·8	6·8 (a)	0·2–100·0	4·1 (a)	2·2–9·5	4·7 (a)	2·5–9·6	11
Mg (%)	6·4	4·0–11·3	9·3 (a)	5·8–16·5	37·0 (ab)	0·0–39·1	4·7 (bc)	3·4–8·0	4·8 (c)	3·3–7·8	18
Phosphorus (%)	14·2	8·7–23·8	19·9 (a)	10·7–32·7	36·4 (ab)	0·0–89·6	11·6 (b)	5·7–17·6	12 (b)	7·8–16·4	28
Fe (%)	4·6	2·5–9·1	6·9 (a)	3·0–14·5	63·7 (ab)	0·0–1824·3	3·5 (b)	2·3–7·3	3·7 (b)	2·2–5·9	129
Cu (%)	5·4	2·8–9·7	7·4 (a)	3·8–15·8	246·5 (ab)	0·0–3500·0	4·3 (b)	2·2–7·6	4·4 (b)	2·4–6·9	223
Zn (%)	7·3	3·7–13·9	11·4 (a)	5·3–20·4	67·6 (ab)	0·0–714·3	5·8 (b)	2·5–9·5	5·6 (b)	3·2–9·1	170
Na (%)	5·2	1·2–10·3	2·1 (a)	0·4–6·1	1·9 (abc)	0·3–1250·0	5·9 (b)	1·3–11·0	8·6 (c)	3·8–13·0	57
Chloride (%)	5·9	2·0–11·3	4·0 (a)	1·6–8·5	0·0 (a)	0·0–8·2	5·9 (a)	1·5–10·5	8·3 (b)	3·5–12·9	74
Iodine (%)	2·8	1·0–6·8	3·4 (a)	1·4–7·9	0·0 (b)	0·0–1·5	5·4 (a)	1·8–8·6	2·2 (b)	0·6–5·2	315
Se (%)	3·9	0·8–7·9	4·9 (a)	0·8–11·3	0·0 (ab)	0·0–24·4	2·3 (b)	0·0–7·9	3·5 (b)	1·3–6·5	443
Overall (/18, Exc VitE, NA, CL) (*n* 1824)	9·9	6·1–18·8	15·7 (a)	9·0–31·7	132·7 (ab)	0·3–386·2	7·9 (b)	5·5–12·4	7·2 (b)	5·1–11·5	9
Overall (/20, Inc NA, CL, Exc VItE)	9·8	6·3–17·6	14·7 (a)	8·7–29·6	120·3 (ab)	0·3–410·1	7·6 (b)	5·4–12·1	7·6 (b)	5·4–11·8	7

MPF, minimally processed food; MTL, multiple traffic light; PCI, processed culinary ingredients; PF, processed food; UPF, ultra-processed food; Exc., excluded; Inc, included; RNI, reference nutrient intake; NDNS, National Diet and Nutrition Survey.

Overall (%) (/18) includes all micronutrients except Na, chloride and vitamin E. Overall (%) (/20) includes all micronutrients except vitamin E. Unlike letters indicate significantly different *P* < 0·05. Pairwise comparisons conducted using Kruskal–Wallis ANOVA with Bonferroni correction for multiple comparisons. Items were classified into ‘healthy’ or ‘unhealthy’ based on the presence or absence of a red FOPL traffic light for fat, saturated fat, total sugar or salt. This is based on research that when identifying healthier products, UK consumers are more cautious to avoid items with red traffic lights, than to select items with green traffic lights^(26,27)^ and has been previously used in published research^(14)^. Se: total = 1824 (UPF, *n* 841).

The average percentage contribution to daily recommendations of key micronutrients of unhealthy items for females aged 19–64 years is reported in Fig. 2 and online Supplementary Tables 12 (per 100 g) and 13 (per 100 kcal). *P*-values comparing healthy and unhealthy items are in online Supplementary Table 14. Compared with healthy items per 100 g, unhealthy items would provide higher average contributions to female micronutrient recommendations (unhealthy: 13·1 % (IQR: 8·0–22·7); healthy: 10·3 % (IQR: 6·3–16·4), *P* < 0·001). Within MPF, PF and UPF, unhealthy items would also provide higher average micronutrient content than healthy items in the same Nova group. Unhealthy MPF would provide significantly greater average micronutrient contributions per 100 g (25·7 % (IQR: 13·0–45·1) compared with UPF (12·0 % (IQR: 7·5–17·8), but not PF (18·8 % (IQR: 11·1–30·7)).

Compared with healthy items per 100 kcal, unhealthy items would provide lower average contributions to female micronutrient recommendations (unhealthy: 4·4 % (IQR: 2·2–8·6); healthy: 9·9 % (IQR: 6·1–18·8), *P* < 0·001). Within MPF, PF and UPF, unhealthy items would provide lower average micronutrient contributions per 100 kcal than healthy items in the same Nova group. Unhealthy MPF would contribute significantly greater average micronutrient content per 100 kcal (9·8 % (IQR: 6·0–15·2)) than unhealthy UPF (3·5 % (IQR: 2·0–7·0)) or unhealthy PF (6·8 % (IQR: 3·5–9·3)). Healthy UPF and MPF would provide significantly greater contributions to Na recommendations per 100 kcal than unhealthy UPF (healthy: 8·6 % (IQR: 3·8–13·0) *v*. unhealthy: 4·5 % (IQR: 1·9–11·3)) and MPF (healthy: 2·1 % (IQR: 0·4–6·1) *v*. unhealthy: 1·4 % (IQR: 0·2–4·1)), respectively (both *P* < 0·001).

Average percentage contributions to daily recommendations of key micronutrients of healthy items for males aged 19–64 years are reported in online Supplementary Table 15 (per 100 g) and 16 (per 100 kcal), and unhealthy items in online Supplementary Table 17 (per 100 g) and 18 (per 100 kcal). Findings for percentage contributions to daily micronutrient recommendations within and between healthy and unhealthy items were similar for males aged 19–64 years, both per 100 g and per 100 kcal.

### Contributions of food and drink to recommended micronutrient intakes, independent of multiple traffic light front-of-package labels

Table 3 reports the binary regression models between Nova groups, FOPL score and micronutrient contributions per 100 g and per 100 kcal for females and males aged 19–64 years. After accounting for a healthy/unhealthy FOPL, compared with MPF, UPF had lower odds of an above median micronutrient contribution (i.e. content) per 100 g (OR: 0·76 (95 % CI 0·64, 0·90), *P* < 0·001), as did PCI (OR: 0·16 (95 % CI 0·09, 0·31), *P* < 0·001). Compared with MPF, PF had similar odds of an above median micronutrient contribution (i.e. content) per 100 g (OR: 0·95 (95 % CI 0·72, 1·24), *P* < 0·689). Per 100 kcal, MPF had higher odds of an above median micronutrient contribution per 100 kcal than UPF (OR: 5·9 (95 % CI 4·9, 7·2)), and PF (OR 3·2 (95 % CI 2·4, 4·2)). Results were similar for overall micronutrient contributions for males aged 19–64 years. Per 100 g, unhealthy items had higher odds of an above median micronutrient contribution than healthy items (OR: 1·9 (95 % CI 1·6, 2·2), *P* < 0·001). Per 100 kcal, healthy items had higher odds of an above median micronutrient contribution than unhealthy items (OR: 2·6 (95 % CI 2·2, 3·0), *P* < 0·001). Per 100 kcal, healthy items also had higher odds of an above median Na contribution than unhealthy items (OR: 2·0 (95 % CI 1·7, 2·4), *P* < 0·001).

**Table 3.** Binary regression models between Nova groups, MTL front-of-package label score and percentage micronutrient contributions per 100 g and per 100 kcal, for females aged 19–64 years (Beta and 95 % confidence intervals)

	% Contribution per 100 g			% Contribution per 100 kcal		
		95 % CI			95 % CI	
Micronutrient	Exp (β)	Lower, Upper	*P*	Exp (β)	Lower, Upper	*P*
Vitamin A (retinol equivalents) (%)						
Nova						
MPF	Reference	Reference	Reference	Reference	Reference	Reference
PCI	0·330	0·186, 0·588	< 0·001	0·398	0·222, 0·712	0·002
PF	1·563	1·187, 2·057	0·001	1·568	1·187, 2·072	0·002
UPF	0·831	0·704, 0·982	0·030	0·67	0·567, 0·792	< 0·001
FOPL						
Unhealthy (one or more red MTL)	Reference	Reference	Reference	Reference	Reference	Reference
Healthy (no red MTL)	0·683	0·583, 0·800	< 0·001	1·096	0·935, 1·284	0·259
Vitamin D (%)						
Nova						
MPF	Reference	Reference	Reference	Reference	Reference	Reference
PCI	0·707	0·384, 1·299	0·263	0·777	0·42, 1·437	0·421
PF	3·571	2·704, 4·715	< 0·001	3·548	2·687, 4·685	< 0·001
UPF	1·876	1·575, 2·235	< 0·001	1·893	1·588, 2·256	< 0·001
FOPL						
Unhealthy (one or more red MTL)	Reference	Reference	Reference	Reference	Reference	Reference
Healthy (no red MTL)	0·867	0·739, 1·018	0·082	0·885	0·754, 1·039	0·137
Thiamin (%)						
Nova						
MPF	Reference	Reference	Reference	Reference	Reference	Reference
PCI	0·062	0·019, 0·201	< 0·001	0·098	0·041, 0·235	< 0·001
PF	0·578	0·436, 0·767	< 0·001	0·197	0·145, 0·267	< 0·001
UPF	1·236	1·047, 1·459	0·012	0·396	0·331, 0·475	< 0·001
FOPL						
Unhealthy (one or more red MTL)	Reference	Reference	Reference	Reference	Reference	Reference
Healthy (no red MTL)	0·936	0·799, 1·097	0·415	3·802	3·205, 4·51	< 0·001
Riboflavin (%)						
Nova						
MPF	Reference	Reference	Reference	Reference	Reference	Reference
PCI	0·266	0·146, 0·485	< 0·001	0·209	0·113, 0·387	< 0·001
PF	1·308	0·995, 1·72	0·054	0·629	0·479, 0·828	< 0·001
UPF	1·104	0·932, 1·307	0·252	0·334	0·281, 0·397	< 0·001
FOPL						
Unhealthy (one or more red MTLs)	Reference	Reference	Reference	Reference	Reference	Reference
Healthy (no red MTL)	0·425	0·362, 0·499	< 0·001	1·397	1·188, 1·642	< 0·001
Niacin equivalent (%)						
Nova						
MPF	Reference	Reference	Reference	Reference	Reference	Reference
PCI	0·057	0·018, 0·184	< 0·001	0·05	0·015, 0·163	< 0·001
PF	1·606	1·226, 2·103	< 0·001	0·593	0·449, 0·784	< 0·001
UPF	1·356	1·149, 1·601	< 0·001	0·46	0·387, 0·548	< 0·001
FOPL						
Unhealthy (one or more red MTL)	Reference	Reference	Reference	Reference	Reference	Reference
Healthy (no red MTL)	0·799	0·683, 0·936	0·006	2·915	2·471, 3·438	< 0·001
Vitamin C (%)						
Nova						
MPF	Reference	Reference	Reference	Reference	Reference	Reference
PCI	0·201	0·099, 0·405	< 0·001	0·215	0·106, 0·436	< 0·001
PF	0·829	0·629, 1·091	0·181	0·818	0·620, 1·078	0·153
UPF	0·55	0·464, 0·652	< 0·001	0·552	0·466, 0·656	< 0·001
FOPL						
Unhealthy (one or more red MTL)	Reference	Reference	Reference	Reference	Reference	Reference
Healthy (no red MTL)	2·373	2·019, 2·790	< 0·001	2·435	2·070, 2·864	< 0·001
Vitamin E (%)						
Nova						
MPF	Reference	Reference	Reference	Reference	Reference	Reference
PCI	0·692	0·405, 1·183	0·179	0·358	0·198, 0·647	< 0·001
PF	1·059	0·806, 1·391	0·683	0·756	0·578, 0·988	0·041
UPF	1·256	1·061, 1·486	0·008	0·708	0·599, 0·836	< 0·001
FOPL						
Unhealthy (one or more red MTL)	Reference	Reference	Reference	Reference	Reference	Reference
Healthy (no red MTL)	0·457	0·389, 0·536	< 0·001	1·155	0·987, 1·352	0·073
Vitamin B_6_ (%)	2 outliers					
Nova						
MPF	Reference	Reference	Reference	Reference	Reference	Reference
PCI	0·039	0·009, 0·161	< 0·001	0·027	0·008, 0·088	< 0·001
PF	0·513	0·382, 0·688	< 0·001	0·27	0·203, 0·359	< 0·001
UPF	0·616	0·518, 0·733	< 0·001	0·22	0·183, 0·265	< 0·001
FOPL						
Unhealthy (one or more red MTL)	Reference	Reference	Reference	Reference	Reference	Reference
Healthy (no red MTL)	0·714	0·604, 0·843	< 0·001	2·188	1·851, 2·588	< 0·001
Vitamin B_12_ (%)						
Nova						
MPF	Reference	Reference	Reference	Reference	Reference	Reference
PCI	0·577	0·321, 1·036	0·066	0·637	0·352, 1·153	0·136
PF	2·803	2·126, 3·695	< 0·001	2·781	2·109, 3·666	< 0·001
UPF	1·796	1·515, 2·130	< 0·001	1·807	1·523, 2·144	< 0·001
FOPL						
Unhealthy (one or more red MTL)	Reference	Reference	Reference	Reference	Reference	Reference
Healthy (no red MTL)	0·695	0·593, 0·814		0·707	0·603, 0·829	< 0·001
Folate (%)						
Nova						
MPF	Reference	Reference	Reference	Reference	Reference	Reference
PCI	0·072	0·026, 0·201	< 0·001	0·114	0·047, 0·274	< 0·001
PF	0·941	0·719, 1·231	0·657	0·569	0·428, 0·756	< 0·001
UPF	0·824	0·698, 0·974	0·023	0·442	0·370, 0·527	< 0·001
FOPL						
Unhealthy (one or more red MTL)	Reference	Reference	Reference	Reference	Reference	Reference
Healthy (no red MTL)	1·447	1·235, 1·694	< 0·001	3·702	3·129, 4·381	< 0·001
Potassium (%)						
Nova						
MPF	Reference	Reference	Reference	Reference	Reference	Reference
PCI	0·069	0·032, 0·149	< 0·001	0·063	0·029, 0·139	< 0·001
PF	0·309	0·234, 0·408	< 0·001	0·201	0·148, 0·272	< 0·001
UPF	0·418	0·352, 0·496	< 0·001	0·107	0·087, 0·132	< 0·001
FOPL						
Unhealthy (one or more red MTL)	Reference	Reference	Reference	Reference	Reference	Reference
Healthy (no red MTL)	0·735	0·626, 0·864	< 0·001	4·072	3·390, 4·891	< 0·001
Ca (%)						
Nova						
MPF	Reference	Reference	Reference	Reference	Reference	Reference
PCI	0·544	0·305, 0·971	0·04	0·638	0·351, 1·159	0·14
PF	2·128	1·618, 2·8	< 0·001	1·312	0·998, 1·726	0·052
UPF	2·458	2·070, 2·918	< 0·001	1·059	0·895, 1·254	0·503
FOPL						
Unhealthy (one or more red MTL)	Reference	Reference	Reference	Reference	Reference	Reference
Healthy (no red MTL)	0·529	0·45, 0·622	< 0·001	2·228	1·897, 2·616	< 0·001
Mg (%)						
Nova						
MPF	Reference	Reference	Reference	Reference	Reference	Reference
PCI	0·094	0·042, 0·210	< 0·001	0·117	0·057, 0·242	< 0·001
PF	0·598	0·454, 0·786	< 0·001	0·186	0·138, 0·25	< 0·001
UPF	0·914	0·774, 1·079	0·287	0·197	0·163, 0·239	< 0·001
FOPL						
Unhealthy (one or more red MTL)	Reference	Reference	Reference	Reference	Reference	Reference
Healthy (no red MTL)	0·610	0·520, 0·715	< 0·001	3·705	3·111, 4·412	< 0·001
Phosphorus (%)						
Nova						
MPF	Reference	Reference	Reference	Reference	Reference	Reference
PCI	0·078	0·031, 0·199	< 0·001	0·118	0·054, 0·255	< 0·001
PF	1·68	1·277, 2·210	< 0·001	0·553	0·418, 0·73	< 0·001
UPF	1·541	1·302, 1·823	< 0·001	0·337	0·282, 0·402	< 0·001
FOPL						
Unhealthy (one or more red MTL)	Reference	Reference	Reference	Reference	Reference	Reference
Healthy (no red MTL)	0·468	0·398, 0·550	< 0·001	2·446	2·074, 2·884	< 0·001
Fe (%)						
Nova						
MPF	Reference	Reference	Reference	Reference	Reference	Reference
PCI	0·157	0·078, 0·317	< 0·001	0·107	0·047, 0·242	< 0·001
PF	0·603	0·456, 0·797	< 0·001	0·329	0·247, 0·437	< 0·001
UPF	1·139	0·964, 1·346	0·127	0·383	0·321, 0·457	< 0·001
FOPL						
Unhealthy (one or more red MTL)	Reference	Reference	Reference	Reference	Reference	Reference
Healthy (no red MTL)	0·53	0·452, 0·622	< 0·001	2·717	2·303, 3·205	< 0·001
Cu (%)						
Nova						
MPF	Reference	Reference	Reference	Reference	Reference	Reference
PCI	0·207	0·107, 0·40	< 0·001	0·122	0·057, 0·263	< 0·001
PF	0·815	0·618, 1·076	0·149	0·436	0·331, 0·574	< 0·001
UPF	1·525	1·288, 1·804	< 0·001	0·46	0·388, 0·547	< 0·001
FOPL						
Unhealthy (one or more red MTL)	Reference	Reference	Reference	Reference	Reference	Reference
Healthy (no red MTL)	0·457	0·389, 0·537	< 0·001	1·796	1·529, 2·109	< 0·001
Zn (%)						
Nova						
MPF	Reference	Reference	Reference	Reference	Reference	Reference
PCI	0·123	0·057, 0·265	< 0·001	0·105	0·049, 0·227	< 0·001
PF	1·223	0·933, 1·604	0·144	0·508	0·385, 0·671	< 0·001
UPF	1·091	0·923, 1·289	0·31	0·302	0·253, 0·360	< 0·001
FOPL						
Unhealthy (one or more red MTL)	Reference	Reference	Reference	Reference	Reference	Reference
Healthy (no red MTL)	0·540	0·460, 0·633	< 0·001	2·149	1·823, 2·534	< 0·001
Na (%)						
Nova						
MPF	Reference	Reference	Reference	Reference	Reference	Reference
PCI	0·790	0·407, 1·532	< 0·001	0·488	0·216, 1·100	0·084
PF	7·412	5·515, 9·962	< 0·001	4·191	3·157, 5·563	< 0·001
UPF	8·049	6·618, 9·790	< 0·001	4·336	3·616, 5·201	< 0·001
FOPL						
Unhealthy (one or more red MTL)	Reference	Reference	Reference	Reference	Reference	Reference
Healthy (no red MTL)	0·630	0·530, 0·748	< 0·001	2·022	1·709, 2·394	< 0·001
Chloride (%)						
Nova						
MPF	Reference	Reference	Reference	Reference	Reference	Reference
PCI	0·562	0·269, 1·172	0·124	0·306	0·129, 0·728	0·007
PF	7·874	5·849, 10·601	< 0·001	2·913	2·200, 3·857	< 0·001
UPF	7·814	6·427, 9·500	< 0·001	2·523	2·119, 3·005	< 0·001
FOPL						
Unhealthy (one or more red MTL)	Reference	Reference	Reference	Reference	Reference	Reference
Healthy (no red MTL)	0·643	0·541, 0·764	< 0·001	2·344	1·987, 2·766	< 0·001
Iodine (%)						
Nova						
MPF	Reference	Reference	Reference	Reference	Reference	Reference
PCI	0·370	0·205, 0·668	< 0·001	0·269	0·142, 0·510	< 0·001
PF	3·229	2·418, 4·311	< 0·001	1·464	1·108, 1·933	0·007
UPF	1·577	1·330, 1·871	< 0·001	0·644	0·545, 0·762	< 0·001
FOPL						
Unhealthy (one or more red MTL)	Reference	Reference	Reference	Reference	Reference	Reference
Healthy (no red MTL)	0·426	0·362, 0·501	< 0·001	1·333	1·137, 1·563	< 0·001
Se (%)						
Nova						
MPF	Reference	Reference	Reference	Reference	Reference	Reference
PCI	0·131	0·051, 0·331	< 0·001	0·105	0·041, 0·270	< 0·001
PF	1·572	1·201, 2·057	< 0·001	0·643	0·488, 0·847	0·002
UPF	1·23	1·039, 1·457	0·016	0·656	0·553, 0·779	< 0·001
FOPL						
Unhealthy (one or more red MTLs)	Reference	Reference	Reference	Reference	Reference	Reference
Healthy (no red MTL)	0·86	0·734, 1·008	0·062	2·639	2·242, 3·106	< 0·001
Female Overall (%) (/18, Exc VitE, NA, CL)						
Nova						
MPF	Reference	Reference	Reference	Reference	Reference	Reference
PCI	0·163	0·086, 0·309	< 0·001	0·086	0·041, 0·181	< 0·001
PF	0·946	0·722, 1·241	0·689	0·316	0·237, 0·422	< 0·001
UPF	0·758	0·641, 0·896	0·001	0·169	0·139, 0·204	< 0·001
FOPL						
Unhealthy (one or more red MTL)	Reference	Reference	Reference	Reference	Reference	Reference
Healthy (no red MTL)	0·532	0·453, 0·624	< 0·001	2·562	2·159, 3·042	< 0·001
Male Overall (%) (/18, Exc VitE, NA, CL)						
Nova						
MPF	Reference	Reference	Reference	Reference	Reference	Reference
PCI	0·138	0·072, 0·267	< 0·001	0·092	0·044, 0·194	< 0·001
PF	0·874	0·666, 1·146	0·330	0·333	0·249, 0·444	< 0·001
UPF	0·727	0·614, 0·860	< 0·001	0·176	0·145, 0·212	< 0·001
FOPL						
Unhealthy (one or more red MTLs)	Reference	Reference	Reference	Reference	Reference	Reference
Healthy (no red MTL)	0·502	0·427, 0·589	< 0·001	2·820	2·374, 3·349	< 0·001

FOPL, front-of-package label; IQR, interquartile range; MPF, minimally processed food; MTL, multiple traffic light; PCI, processed culinary ingredients; PF, processed food; UPF, ultra-processed food.

Overall (%) (/18) includes all micronutrients except Na, chloride and vitamin E. Items were classified into ‘healthy’ or ‘unhealthy’ based on the presence or absence of a red FOPL traffic light for fat, saturated fat, total sugar or salt. This is based on research that when identifying healthier products, UK consumers are more cautious to avoid items with red traffic lights, than to select items with green traffic lights^(26,27)^.

### Micronutrient-dense food and drink items

UPF in the top 25 % of all items contributing to micronutrient recommendations per 100 kcal included ready-to-drink lower energy fruit juices/squash, drinkable/fortified/fruit yogurts, reduced fat and yeast extract spreads, baby formula (made up), meal replacement drinks/bars liver-based products (patepate, sausage), beef-based ready meals/dishes, mocha, fish in breadcrumbs, lean/low-fat processed meats, breakfast cereals, plant-based meat alternatives and fortified plant-based milk alternatives. Of which items with an unhealthy FOPL spanned reduced fat and yeast extract spreads, baby formula, meal replacement bars, liver-based products, mocha, breakfast cereals and lean/low-fat processed meats. PF in the top 25 % of all items contributing to micronutrient recommendations per 100 kcal included tinned fish, canned soup, sauerkraut and gherkins. The top 10 % of all items contributing to micronutrient recommendations per 100 kcal included several UPF, such as ready-to-drink lower energy fruit juices/squash, drinkable yogurts, yeast extract spreads, liver-based products (pate and sausage), plant-based meat burger and fortified soya milks.

## Discussion

This analysis indicates variation across UK food and drink in meeting government micronutrient recommendations, based on degree of processing and FOPL. Per 100 g, MPF, PF and UPF provided similar average contributions to recommended intakes. However, per 100 kcal, MPF provided on average nearly double the micronutrient content of PF, and nearly two and a half times more than UPF. While PF per 100 kcal also provided nearly a third more micronutrient content than UPF. Micronutrient contributions also differed between healthy and unhealthy items based on the presence of one or more red FOPL. Per 100 g, unhealthy items provided on average 27 % greater contributions to micronutrient recommendations than healthy items. However, per 100 kcal, contributions to micronutrient recommendations of healthy items were on average over double that of unhealthy items. Furthermore, the significant differences in average micronutrient contributions per 100 kcal between MPF, PF and UPF were still observed within healthy items. Per 100 kcal, healthy MPF provided significantly higher contributions than both healthy PF and UPF, where healthy PF and UPF were not significantly different from each other and provided around half the micronutrient contribution of MPF. These data therefore suggest that the degree of processing may impact on the micronutrient content of an individual’s diet.

Previous work on the UK NDNS demonstrated partial overlap between FOPL and the degree of processing in the UK food and drink^(14)^. In general, UPF had a poorer nutrient profile than MPF, but generally similar to PF. This was unchanged after considering only items with healthy FOPL^(14)^. This analysis builds upon these findings by showing that the micronutrient content and respective contributions to government micronutrient recommendations of food and drink in the UK also differs by degree of processing, and the differences are still observed within healthy items. UPF tended to be in the lower quartiles of average micronutrient contributions, and items with zero content for a given micronutrient content were generally over-represented by UPF. As in the previous analysis, some UPF had a nutritional profile comparable to MPF when looking at fat, saturated fat, salt and sugar^(14)^ and would be considered healthy. This analysis of micronutrient content shows similar findings; about 25 % of items in the highest quartile of average micronutrient contributions per 100 kcal were UPF. These items spanned a range of UPF subgroups, including fruit juices/squash, yogurts, ready meals, fish products, breakfast cereals and plant-based meat and milk alternatives. Our data questions the suggestions made by some authors that UPF are relatively deficient in micronutrients^(28)^ and should all be avoided in favour of MPF.

Previous studies across several nations have shown that high-UPF diets tend to contain a lower micronutrient content^(13)^. However, to date, few studies have examined the micronutrient content of food and drink across a nationally representative database based on the degree of processing and none in the UK. In the USA, an analysis of the approximately 370 food and drink items captured in a FFQ demonstrated that UPF tended to have a poorer nutrient density compared with MPF, based on Nutrient Rich Food (NRF_9·3_) score (including vitamins A, C, E, Ca, Mg and potassium, but also saturated fat, added sugar, and Na content per 100 kcal)^(29,30)^. However, similar to this study, some UPF scored well. Our results expand upon these findings by demonstrating similar findings across a UK database of nearly 3000 food and drink items, with greater detail to classify items into the Nova classification as previously described^(12)^.

Arguments for reducing UPF intake have included their displacement of ‘real food’ and their ‘intrinsically unhealthy’ properties^(4)^. The results here indicate that after adjusting for energy content and FOPL score, UPF had a poorer micronutrient profile than MPF. Our analysis of Year 9–11 NDNS data indicates that for a number of micronutrients, large proportions of adults do not meet the recommended RNI, particularly for several minerals, including potassium. When considering micronutrient contributions per 100 kcal reported here as a 2000 kcal diet, as recommended for females aged 19–64 years^(11)^, only five out of twenty micronutrient RNI recommendations would be met with a diet composed solely of UPF (two of which being Na and Cl), compared with twelve for a diet solely of MPF (which does not include Na or Cl). Diets solely of UPF or MPF would also provide at least 80 % of RNI recommendations for further two micronutrients. When considering micronutrient contributions from healthy UPF per 100 kcal for a 2000 kcal diet, only six micronutrient recommendations would still be met (two of which being Na and Cl). However, a diet solely from healthy UPF would provide at least 80 % of RNI recommendations for a further six micronutrients. This may imply that a healthy UPF diet (i.e. avoiding items with a red FOPL) would be less micronutrient dense and less likely to meet micronutrient recommendations than an energy-matched, healthy MPF diet. On average in the UK, UPF contributes to over 50 % of daily energy intake, with nearly one-third of energy intake provided by MPF, and only about 10 % by PF^(31)^. Given the large contributions of UPF to daily energy intake, the lower micronutrient content of UPF compared with MPF has potentially important implications for meeting micronutrient recommendations within the UK population. In contrast, a US modelling study suggested that UPF are necessary to achieve a nutritionally adequate diet, with UPF making considerable contributions to vitamin E, thiamin, niacin, folate and Ca intake^(32)^. Furthermore, modelling studies indicate the potential for nutritional deficiency with avoidance of some fortified UPF^(33)^. In this study, the micronutrient content of healthy PF per 100 kcal was also significantly lower than MPF and similar to UPF. This might imply that a healthy PF diet would also be less likely to meet micronutrient recommendations than an energy-matched, healthy MPF diet, but not more likely than an energy-matched, healthy UPF diet. However, in contrast to the wide range of micronutrient-dense UPF, the range of micronutrient-dense PF in the highest quartile of micronutrient contributions was limited (e.g. tinned fish, soup, sauerkraut and gherkins), potentially limiting the ability to construct a diet solely from healthy PF.

These findings have potential implications for how consumers interpret FOPL and the healthiness of foods and drinks. It is unclear whether FOPL, Nova, or both, are most valuable for identifying micronutrient-dense products and thus the value of the degree of processing in addition to current UK governmental guidance. SACN reported insufficient evidence to justify incorporating ultra-processing into the EWG^(7)^, due to limitations in the largely observational evidence, and that the adverse associations may possible be covered in current UK dietary guidance. Reformulated products, such as UPF lower in fat, saturated fat, salt, sugar or higher in fibre, can include FOPL nutrition claims. Importantly, nutrition claims influence consumer choice, including purchase intentions, consumption guilt, expected tastiness and consumption^(34)^. The implications of this messaging across UPF varying in overall nutritional content requires further investigation and may further confuse the consumer in the retail environment. For example, in this analysis, healthy UPF contained more Na per 100 kcal than unhealthy UPF, given the higher energy density of unhealthy UPF. An ongoing trial assessing healthy MPF *v*. UPF diets meeting the UK EWG will provide valuable insights into the health impacts of nutritionally improved UPF with nutrition claims^(35)^. The UPF diet includes subgroups in the highest quartile of average micronutrient contributions per 100 kcal in this study (including squash, yogurts, ready meals, breakfast cereals and plant-based alternatives)^(35)^.

Discussion on UPF must consider that lower income in the UK is associated with poorer dietary quality^(36)^ and lower social classes with higher UPF intakes^(37)^. With UPF tending to be cheaper than MPF^(29,38)^, many individuals are forced to choose the food which they can afford. Therefore, any policy or legislative action must therefore consider the potential wider social consequences of people reducing their UPF intake.

### Strengths and limitations

Strengths of this study include the nationally representative database of food and drink items for the UK, with a matching nutrient database containing average nutrient compositions. The micronutrient content of items was compared by weight (per 100 g) and energy (per 100 kcal), as well as after considering existing governmental dietary guidance provided through FOPL MTL. Limitations include the lack of analysis based on actual portion size or food consumed. Whilst analyses were conducted per 100 g and 100 kcal allowing for uniform comparability between food and drink items, they may not reflect the micronutrient intakes of actual portion sizes or foods eaten by consumers. However, as the UK government recommends a 2000 kcal per d for females and 2500 kcal per d for males, the analysis per 100 kcal provides important insights into micronutrient contributions for a recommended diet according to degree of processing. However, this assumes items within each grouping are consumed in equivalent amounts, which may not represent actual intakes in a real-world setting and should be taken into account when interpreting the data. The actual average UK adult micronutrient intakes in the total diet and per 100 kcal were therefore also included for comparison. Across food processing classifications, Nova has been most used. However, Nova has been criticised for difficulties in its application^(39)^, with reports of coding inconsistencies^(40)^. Despite this, several studies with multiple coders report that most items are consistently coded using Nova, with misclassified or ambiguous items tending to be only 5–10 % of all items^(41–43)^. Furthermore, the SACN report on food processing also highlighted that Nova was the only food processing classification that met their five criteria, including its applicability to individuals in the UK^(51)^. In this analysis, there was author agreement on classifying items.

### Conclusions

Across a nationally representative food and drink database, MPF, PF and UPF per 100 g provide similar average contributions to UK government micronutrient recommendations. However, contributions significantly differed when compared per 100 kcal. MPF provided the greatest average contributions per 100 kcal, followed by PF and then UPF. Healthy items with no red FOPL provided higher average contributions to micronutrient recommendations per 100 kcal than unhealthy items. Observed differences in average contributions between Nova groups persisted after accounting for healthy/unhealthy FOPL. Within healthy items per 100 kcal, MPF provided the greatest average contributions to micronutrient recommendations, followed by PF and UPF, which were similar. These findings suggest healthy UPF or PF diets would be less likely to meet UK government micronutrient recommendations than an energy-matched healthy MPF diet. The results are important for understanding the healthiness of the UK food and drink supply based on FOPL and degree of processing and implications for future potential policy and legislation regarding UPF.

## Supporting information

Dicken et al. supplementary material Dicken et al. supplementary material

## Supplementary material

For supplementary material/s referred to in this article, please visit https://doi.org/10.1017/S0007114524003374
