# Supplementary material for: Micronutrients or processing? An analysis of food and drink items from the UK National Diet and Nutrition Survey based on micronutrient content, the Nova classification and front-of-package traffic light labelling
Source: Br J Nutr. 2025 Jan 13;133(3):382–99. doi: 10.1017/S0007114524003374 (PMC11946030; doi:10.1017/S0007114524003374)
Supplement: Dicken et al. supplementary material [file S0007114524003374sup001.docx]

**Supplementary Materials**

**NOVA classification (adapted from Monteiro et al., 2019 (1)).**

**Minimally processed foods**

Unprocessed foods altered by industrial processes such as removal of inedible or unwanted parts, drying, crushing, grinding, fractioning, roasting, boiling, pasteurisation, refrigeration, freezing, placing in containers, vacuum packaging, non-alcoholic fermentation, and other methods that do not add salt, sugar, oils or fats or other food substances to the original food.

Examples include fresh, squeezed, chilled, frozen, or dried fruits and vegetables; grains; legumes; meat, poultry, fish; eggs; milk; fruit or vegetable juices (with no added sugar, sweeteners or flavours); flakes or flour made from corn, wheat, oats, or cassava; seeds (with no added salt or sugar); herbs and spices, plain yoghurt; tea, coffee, and drinking water.

**Processed culinary ingredients**

Substances obtained directly from minimally processed foods or from nature by industrial processes such as pressing, centrifuging, refining, extracting or mining. They are used in preparing, seasoning and cooking minimally processed foods.

Examples include vegetable oils; butter and lard; sugar and molasses; honey extracted; starches extracted from corn and other plants, and salt.

**Processed food**

Products made by adding salt, oil, sugar or other processed culinary ingredients to minimally processed foods, using preservation methods such as canning and bottling, or for breads and cheeses, using non-alcoholic fermentation. Processes and ingredients are used to increase the durability of minimally processed foods and make them more enjoyable, by modifying or enhancing sensory qualities.

Examples include canned or bottled vegetables and legumes in brine; salted or sugared nuts and seeds; salted, dried, cured, or smoked meats and fish; canned fish; fruits in syrup and freshly made unpackaged breads and cheeses.

**Ultra-processed food**

Formulations of ingredients mostly of exclusive industrial use, that result from a series of industrial processes. Many processes require sophisticated equipment and technology. Processes enabling the manufacture of ultra-processed foods include fractioning whole foods into substances, chemical modifications of substances, assembly of unmodified and modified food substances using industrial techniques such as extrusion, moulding and pre-frying, frequent application of additives whose function is to make the final product palatable or hyper-palatable (‘cosmetic additives’), and sophisticated packaging, usually with synthetic materials. Ingredients often include sugar, oils and fats, and salt, generally in combination; substances that are sources of energy and nutrients but of no or rare culinary use such as high fructose corn syrup, hydrogenated or interesterified oils, and protein isolates; cosmetic additives such as flavours, flavour enhancers, colours, emulsifiers, sweeteners, thickeners, and anti-foaming, bulking, carbonating, foaming, gelling, and glazing agents; and additives that prolong product duration, protect original properties or prevent proliferation of microorganisms. Processes and ingredients used to manufacture ultra-processed foods are designed to create highly profitable (low cost and long shelf-life), convenient, hyper-palatable snacked products liable to displace all other NOVA food groups, notably minimally processed foods.

Examples include carbonated soft drinks; sweet or savoury packaged snacks; chocolate, confectionery; ice-cream; mass-produced packaged breads and buns; margarines and other spreads; biscuits, pastries, cakes, and cake mixes; breakfast ‘cereals’, ‘cereal’ and ‘energy’ bars; ‘energy’ drinks; milk drinks, ‘fruit’ yoghurts and ‘fruit’ drinks; ‘cocoa’ drinks; ‘instant’ sauces; infant formulas, follow-on milks, other baby products; and ‘health’ and ‘slimming’ products such as meal replacement shakes and powders. Many ready to heat products including pre-prepared pies and pasta and pizza dishes; poultry and fish ‘nuggets’ and ‘sticks’, sausages, burgers, hot dogs, and other reconstituted meat products, and powdered and packaged ‘instant’ soups, noodles and desserts are ultra-processed foods.

**NOVA coding in detail (from Dicken et al., 2024 (2)).**

Classification was determined by considering the food or drink item name, subgroup code, best representation from leading UK supermarkets, and NOVA code of the corresponding food item in the NDNS Year 1 to 11 database.

- If the item was designated a ‘homemade’ subgroup with no added detail and most likely homemade, it is classed as MPF.
- Mixed dishes such as curry, pasta etc if defined as homemade and no other description, they are coded as MPF. If there is a specified PCI, PF or UPF in the food or drink item name, then they are coded as PF or UPF, e.g. homemade cream-based sauces or white wine sauces in a mixed dish, curry in a specified cream/coconut based sauce, or pasta with vegetables in a cream/cheese-based sauce are PF.
- If there is a sauce which is an unspecified recipe and the item is homemade then it is coded as PF, if the sauce is UPF then the dish is UPF.
- If a PF or UPF is fundamental to the dish, it is coded as such - dishes with cheese-based toppings such as lasagne are classed as PF.
- Homemade dishes with mayonnaise (e.g. tuna mayonnaise or potato salad) are PF as a minimum, or UPF. Salads with no dressing are coded as MPF. Unspecified homemade salads or readymade salads with mayonnaise, salad cream or French-dressing are coded as UPF.
- Homemade buns, cakes, pastries, puddings and pancakes are coded as PF, given that they are combinations of MPFs and PCIs. Pies, dumplings or pastry doughs are also PF.
- Homemade stew or meat in gravy (assuming the gravy is from cooking the meat, i.e. use of no OXO cube or gravy granules) is coded as MPF.
- Homemade cottage pie or shepherd’s pie are coded as MPF.
- Homemade battered/breaded fish are coded as PF. Readymade battered/breaded fish are UPF. Unspecified battered/breaded fish are UPF.
- Canned vegetables in an unspecified medium (not described in the food item name or subgroup description) are assumed to be in water and coded as MPF. Stuffed vegetables with an undefined filling are MPF. Canned fruit are assumed to be in a syrup or fruit juice, and therefore are coded as PF.
- Bacon, ham, gammon and similar meats such as deli/pre-packed sliced meat are coded as UPF. Traditional hams (e.g. prosciutto, parma or serrano ham) coded as PF.
- Nut butters are PF.
- Tinned fish in oil, brine or tomato are coded as PF.
- Gluten-free foods are coded as UPF.
- Jams/conserves, marmalades and lemon curd are coded as UPF as typically containing gelling agents in UK supermarkets.
- Breakfast cereals including muesli are coded as UPF. Plain porridge is coded as MPF.
- Items made with a plant-based milk, e.g. lattes/cappuccinos made with plant-based milk or porridge made with plant-based milk are coded as MPF.
- Single cream, double cream and crème fraiche are coded as PCI.
- Chow Mein and Chop Suey (which require soy sauce) are coded as PF. Stir fry (assuming only MPF ingredients) is MPF.

**References**:

1. Monteiro CA, Cannon G, Levy RB, Moubarac JC, Louzada ML, Rauber F, et al. Ultra-processed foods: what they are and how to identify them. *Public Health Nutr*. 2019 Apr;22(5):936–41.
2. Dicken SJ, Batterham RL, Brown A. Nutrients or processing? An analysis of food and drink items from the UK National Diet and Nutrition Survey based on nutrient content, the NOVA classification and front of package traffic light labelling. *British Journal of Nutrition*. 2024;131(9):1619-1632. doi:10.1017/S0007114524000096

**Supplementary Figures**

1. Radar plots of the percentage contributions to UK government dietary micronutrient recommendations for females aged 19-64 for each micronutrient across Nova groups, (a) per 100g and, (b), per 100kcal.

**Supplementary Tables**

1. Front of package label thresholds for fat, saturated fat, total sugar and salt content. Green represents ‘low’, amber ‘medium, and red ‘high’ amounts the nutrient in a food/drink. Multiple traffic light front of package label colours do not represent nutrition/health claims.
2. Daily UK government dietary micronutrient recommendations for females and males aged 19-64 years in the general population, respectively (11) (For females aged 50-64 years, 8.7mg of iron is recommended per day). Percent contributions per 100kcal of reported energy intake from food diaries of adults, aged 19-64 from the National Diet and Nutrition Survey Year 9-11 survey, and percentage of adults, aged 19-64 from the National Diet and Nutrition Survey Year 9-11 survey meeting daily micronutrient recommendations.
3. Average percentage contribution per 100g to UK government dietary micronutrient recommendations from the UK NDNS for females aged 19-64 years, and across each Nova group.
4. Average absolute micronutrient content per 100g, and across each Nova group.
5. Average percentage contribution per 100g to UK government dietary micronutrient recommendations from the UK NDNS for males aged 19-64 years, and across each Nova group.
6. Overall average absolute micronutrient content per 100kcal, and across each Nova group.
7. Average percentage contribution per 100kcal to UK government dietary micronutrient recommendations from the UK NDNS for males aged 19-64 years, and across each Nova group, and percentage of government dietary micronutrient recommendations consumed per 100 kcal of reported energy intake for males, aged 19-64 from the NDNS Year 9-11 survey.
8. Quartiles of average percentage contribution to daily dietary recommendations of food and drink items per 100g and 100kcal, by Nova group and healthy vs. unhealthy FOPL.
9. Average percentage contribution of healthy items per 100g to UK government dietary micronutrient recommendations from the UK NDNS for females aged 19-64 years, and across each Nova group.
10. Average absolute micronutrient content of healthy items per 100g across each Nova group.
11. Average absolute micronutrient content of healthy items per 100kcal across each Nova group.
12. Average percentage contribution of unhealthy items per 100g to UK government dietary micronutrient recommendations from the UK NDNS for females aged 19-64 years, and across each Nova group.
13. Average percentage contribution of unhealthy items per 100kcal to UK government dietary micronutrient recommendations from the UK NDNS for females aged 19-64 years, and across each Nova group.
14. Statistical comparison between average percentage contribution to UK government dietary micronutrient recommendations from the UK NDNS for females aged 19-64 years, of healthy and unhealthy items across each Nova group, per 100g and per 100kcal.
15. Average percentage contribution of healthy items per 100g to UK government dietary micronutrient recommendations from the UK NDNS for males aged 19-64 years, and across each Nova group.
16. Average percentage contribution of healthy items per 100kcal to UK government dietary micronutrient recommendations from the UK NDNS for males aged 19-64 years, and across each Nova group.
17. Average percentage contribution of unhealthy items per 100g to UK government dietary micronutrient recommendations from the UK NDNS for males aged 19-64 years, and across each Nova group.
18. Average percentage contribution of unhealthy items per 100kcal to UK government dietary micronutrient recommendations from the UK NDNS for males aged 19-64 years, and across each Nova group.


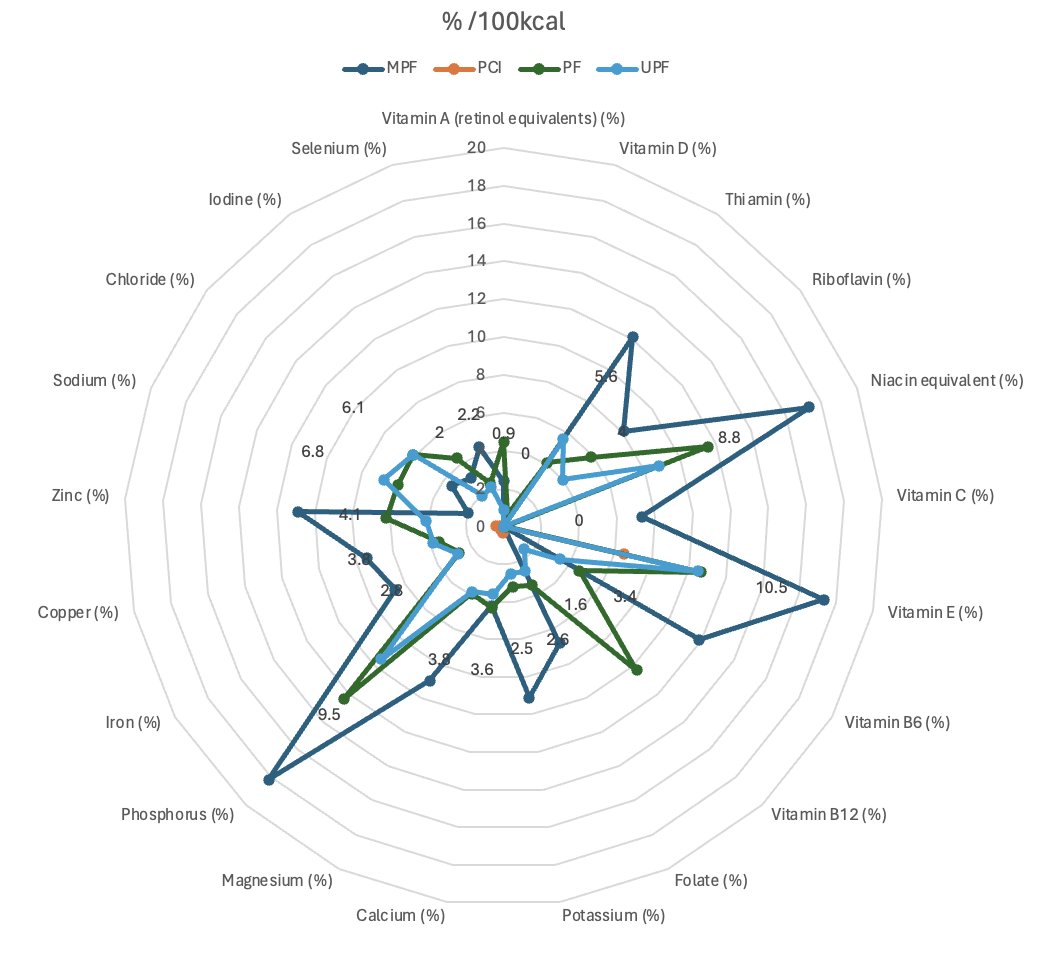
**Figure 1.** Radar plots of the percentage contributions to UK government dietary micronutrient recommendations for females aged 19-64 years for each micronutrient across Nova groups, (a) per 100g and, (b), per 100kcal.


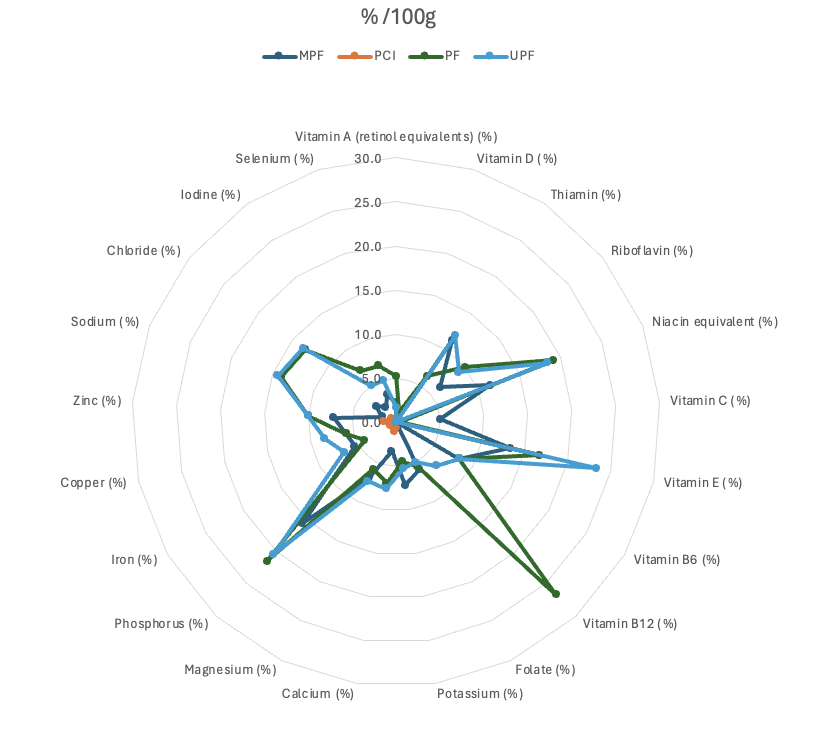


**Table 1. Front of package label thresholds for fat, saturated fat, total sugar and salt content. Green represents ‘low’, amber ‘medium, and red ‘high’ amounts the nutrient in a food/drink. Multiple traffic light front of package label colours do not represent nutrition/health claims.**

| **Nutrient** | **Green FOPL traffic light** | **Amber FOPL traffic light** | **Red FOPL traffic light** |
| --- | --- | --- | --- |
| Fat /100g: Food  Fat /100ml: Drink | ≤ 3.0g  ≤1.5ml | > 3.0g to ≤ 17.5g  > 1.5ml to ≤ 8.75ml | > 17.5g  > 8.75ml |
| Saturated fat /100g: Food  Saturated fat /100ml: Drink | ≤ 1.5g  ≤ 0.75ml | > 1.5g to ≤ 5.0g  > 0.75ml to ≤ 2.5ml | > 5.0g  > 2.5ml |
| Total sugar /100g: Food  Total sugar /100ml: Drink | ≤ 5.0g  ≤ 2.5ml | > 5.0g to ≤ 22.5g  > 2.5ml to ≤ 11.25ml | > 22.5g  > 11.25ml |
| Salt /100g: Food  Salt /100ml: Drink | ≤ 0.3g  ≤ 0.3ml | > 0.3g to ≤ 1.5g  >0.3ml to ≤0.75ml | >1.5g  > 0.75ml |

*FOPL, front of package label; g, grams; ml, millilitres*

**Table 2. Daily UK government dietary micronutrient recommendations for females and males aged 19-64 years in the general population, respectively (11) (For females aged 50-64 years, 8.7mg of iron is recommended per day). Percent contributions per 100kcal of reported energy intake from food diaries of adults, aged 19-64 from the National Diet and Nutrition Survey Year 9-11 survey, and percentage of adults, aged 19-64 from the National Diet and Nutrition Survey Year 9-11 survey meeting daily micronutrient recommendations.**

| **Micronutrient** | **Government daily recommended intake** | | |  | **Reported percentage of RNI consumed in total diet from NDNS Year 9-11** | | | |  | **Percentage of adults meeting RNI from NDNS Year 9-11** | |
| --- | --- | --- | --- | --- | --- | --- | --- | --- | --- | --- | --- |
|  |  |  |  |  | **Females (n = 822)** | | **Males (n = 570)** | |  | **Females (n = 822)** | **Males (n = 570)** |
|  | **Females, aged 19-64** | **Males, aged 19-64** | **Male:Female ratio** |  | **Median (%)** | **IQR** | **Median (%)** | **IQR** |  | **% of adults meeting RNI** | **% of adults meeting RNI** |
| Vitamins |  | | |  |  | | | |  |  | |
| Vitamin A (retinol equivalents) (µg) | 600 | 700 | 1.17 |  | 112.6 | 70.9, 177.4 | 97.3 | 61.7, 157.5 |  | 57.4 | 48.9 |
| Vitamin D (µg) | 10 | 10 | 1.00 |  | 21.6 | 12.5, 36.2 | 25.2 | 15.2, 42.0 |  | 0.7 | 2.6 |
| Thiamin (mg) | 0.8 | 1 | 1.25 |  | 164.9 | 130.0, 204.4 | 165.5 | 127.5, 207.3 |  | 90.9 | 90.5 |
| Riboflavin (mg) | 1.1 | 1.3 | 1.18 |  | 120.5 | 90.1, 157.5 | 120.4 | 94.7, 158.7 |  | 68.0 | 71.8 |
| Niacin equivalent (mg) | 13 | 17 | 1.31 |  | 237.9 | 185.5, 284.2 | 236.3 | 188.8, 287.4 |  | 98.2 | 98.9 |
| Vitamin C (mg) | 40 | 40 | 1.00 |  | 183.7 | 110.5, 276.1 | 182.2 | 113.1, 280.7 |  | 79.1 | 78.8 |
| Vitamin B6 (mg) | 1.2 | 1.4 | 1.17 |  | 118.3 | 94.0, 147.7 | 137.4 | 106.4, 177.2 |  | 69.4 | 78.7 |
| Vitamin B12 (µg) | 1.5 | 1.5 | 1.00 |  | 272.6 | 185.7, 364.6 | 331.2 | 233.7, 445.8 |  | 95.2 | 97.4 |
| Folate (µg) B9 | 200 | 200 | 1.00 |  | 100.0 | 77.4, 129.7 | 125.4 | 97.4, 157.8 |  | 50.1 | 73.2 |
|  |  |  |  |  |  |  |  |  |  |  |  |
| Minerals |  | | |  |  | | | |  |  | |
| Potassium (mg) | 3500 | 3500 | 1.00 |  | 72.3 | 57.5, 86.9 | 87.3 | 70.4, 103.9 |  | 10.0 | 29.0 |
| Calcium (mg) | 700 | 700 | 1.00 |  | 102.2 | 76.1, 126.8 | 119.3 | 92.2, 152.5 |  | 51.6 | 68.5 |
| Magnesium (mg) | 270 | 300 | 1.11 |  | 88.5 | 70.9, 107.6 | 95.7 | 75.7, 118.0 |  | 32.7 | 45.6 |
| Phosphorus (mg) | 550 | 550 | 1.00 |  | 202.2 | 164.0, 240.9 | 246.3 | 200.1, 294.4 |  | 96.6 | 99.2 |
| Iron (mg) | 14.8 | 8.7 | 0.59 |  | 69.9 | 53.9, 93.3 | 128.8 | 98.4, 156.2 |  | 20.7 | 73.6 |
| Copper (mg) | 1.2 | 1.2 | 1.00 |  | 87.7 | 67.9, 108.9 | 100.6 | 78.7, 129.1 |  | 35.2 | 50.4 |
| Zinc (mg) | 7 | 9.5 | 1.36 |  | 106.7 | 85.3, 130.2 | 97.3 | 77.2, 119.0 |  | 57.2 | 44.9 |
| Sodium | 1600 | 1600 | 1.00 |  | 107.2 | 84.2, 137.3 | 142.7 | 107.0, 180.1 |  | 59.2 | 81.5 |
| Chloride (mg) | 2500 | 2500 | 1.00 |  | 117.0 | 92.2, 140.0 | 154.3 | 117.3, 185.9 |  | 67.6 | 86.4 |
| Iodine (µg) | 140 | 140 | 1.00 |  | 88.7 | 63.7, 121.7 | 109.8 | 79.0, 151.9 |  | 38.8 | 58.8 |
| Selenium (µg) | 60 | 75 | 1.25 |  | 68.7 | 52.2, 92.1 | 68.9 | 52.2, 90.5 |  | 18.5 | 17.9 |
|  |  |  |  |  |  |  |  |  |  |  |  |
| Overall (%) /18, Exc NA, CL) |  |  |  |  | 128.1 | 104.5, 157.0 | 143.9 | 115.7, 175.4 |  | 0.0 | 0.7 |
| Overall (%) /20, Inc NA, CL) |  |  |  |  | 127.2 | 102.7, 155.2 | 143.8 | 117.4, 175.3 |  | 0.0 | 0.7 |

*Abbreviations: mg, milligram; µg, microgram; NA, sodium; CL, chloride; Exc, excluded; Inc, included; %, percentage; n, number; RNI, reference nutrient intake; NDNS, National Diet and Nutrition Survey.*

**Table 3. Average percentage contribution per 100g to UK government dietary micronutrient recommendations from the UK NDNS for females aged 19-64** **years, and across each Nova group.**

| **Micronutrient** | **Total (n = 2980)** | | **MPF (n = 986)** | | **PCI (n = 61)** | | **PF (n = 283)** | | **UPF (n = 1650)** | | **Number of items with 0 micronutrient content per 100g** |
| --- | --- | --- | --- | --- | --- | --- | --- | --- | --- | --- | --- |
|  | **Median** | **IQR** | **Median** | **IQR** | **Median** | **IQR** | **Median** | **IQR** | **Median** | **IQR** |  |
| Vitamin A (retinol equivalents) (%) | 2.2 | 0.0, 11.3 | 2.2 (a) | 0.0, 10.0 | 0.0 (b) | 0.0, 51.8 | 5.2 (c) | 0.5, 20.7 | 1.7 (b) | 0.0, 10.2 | 974 |
| Vitamin D (%) | 0.0 | 0.0, 2.4 | 0.0 (a) | 0.0, 1.0 | 0.0 (a) | 0.0, 0.4 | 1.2 (b) | 0.0, 6.0 | 0.0 (c) | 0.0, 2.5 | 1758 |
| Thiamin (%) | 11.3 | 3.8, 21.3 | 11.3 (a) | 5.0, 19.1 | 0.0 (b) | 0.0, 1.3 | 6.3 (c) | 2.5, 13.8 | 11.9 (a) | 3.8, 23.8 | 356 |
| Riboflavin (%) | 8.2 | 2.7, 16.4 | 6.4 (a) | 2.7, 17.3 | 0.0 (b) | 0.0, 15.0 | 10.0 (c) | 2.7, 20.0 | 9.1 (ac) | 3.6, 16.4 | 308 |
| Niacin equivalent (%) | 16.2 | 6.2, 37.7 | 11.5 (a) | 5.4, 42.5 | 0.0 (b) | 0.0, 2.7 | 19.2 (a) | 6.9, 43.1 | 18.5 (a) | 6.9, 36.9 | 196 |
| Vitamin C (%) | 0.0 | 0.0, 11.5 | 5.0 (a) | 0.0, 35.4 | 0.0 (b) | 0.0, 0.0 | 0.5 (c) | 0.0, 9.5 | 0.0 (d) | 0.0, 4.6 | 1505 |
| Vitamin E (%) | 16.7 | 3.3, 50.0 | 13.3 (a) | 3.3, 34.2 | 16.7 (ab) | 0.0, 116.7 | 16.7 (ab) | 6.7, 43.3 | 23.3 (b) | 6.7, 60.0 | 484 |
| Vitamin B6 (%) | 8.3 | 0.0, 16.7 | 8.3 (a) | 8.3, 25.0 | 0.0 (a) | 0.0, 0.0 | 8.3 (b) | 8.3, 16.7 | 8.3 (c) | 0.0, 16.7 | 829 |
| Vitamin B12 (%) | 0.0 | 0.0, 40.0 | 0.0 (a) | 0.0, 46.7 | 0.0 (a) | 0.0, 6.7 | 26.7 (b) | 0.0, 66.7 | 6.7 (c) | 0.0, 33.3 | 1607 |
| Folate (%) | 5.0 | 2.5, 12.0 | 6.0 (a) | 3.0, 15.0 | 0.0 (b) | 0.0, 0.5 | 5.5 (ac) | 2.5, 11.0 | 5.0 (c) | 2.0, 11.1 | 301 |
|  |  |  |  |  |  |  |  |  |  |  |  |
| Potassium (%) | 5.7 | 3.5, 8.9 | 7.2 (a) | 4.7, 10.3 | 0.6 (b) | 0.0, 2.9 | 4.5 (c) | 2.7, 7.3 | 5.3 (c) | 3.3, 7.7 | 49 |
| Calcium (%) | 5.6 | 2.0, 14.3 | 3.3 (a) | 1.6, 7.9 | 1.0 (b) | 0.0, 8.3 | 7.0 (c) | 2.6, 16.1 | 7.6 (c) | 2.4, 15.7 | 46 |
| Magnesium (%) | 7.0 | 4.1, 11.1 | 7.0 (a) | 4.1, 11.1 | 0.4 (b) | 0.0, 3.2 | 5.9 (c) | 4.1, 8.9 | 7.4 (ac) | 4.1, 11.9 | 74 |
| Phosphorus (%) | 18.2 | 6.6, 32.6 | 15.6 (a) | 5.5, 32.7 | 0.6 (b) | 0.0, 10.0 | 21.5 (c) | 5.5, 37.5 | 20.4 (c) | 9.4, 32.2 | 82 |
| Iron (%) | 6.1 | 2.0, 11.5 | 5.4 (a) | 2.0, 12.8 | 0.7 (b) | 0.0, 2.7 | 4.1 (c) | 2.0, 8.1 | 6.8 (a) | 2.7, 11.5 | 232 |
| Copper (%) | 6.7 | 2.5, 13.3 | 5.8 (a) | 2.5, 11.7 | 0.8 (b) | 0.0, 5.4 | 5.8 (a) | 2.5, 9.2 | 8.3 (c) | 3.3, 15.0 | 395 |
| Zinc (%) | 8.6 | 4.3, 17.1 | 7.1 (a) | 2.9, 21.4 | 1.4 (b) | 0.0, 4.3 | 10.0 (a) | 4.3, 21.4 | 10.0 (a) | 4.3, 17.1 | 291 |
| Sodium (%) | 6.8 | 1.3, 21.4 | 1.7 (a) | 0.3, 5.0 | 0.7 (a) | 0.0, 3.1 | 14.0 (b) | 2.8, 26.1 | 14.4 (b) | 4.5, 26.6 | 99 |
| Chloride (%) | 6.8 | 2.3, 20.6 | 2.9 (a) | 1.2, 5.7 | 0.7 (b) | 0.0, 3.1 | 13.2 (c) | 3.9, 24.7 | 13.5 (c) | 4.5, 26.4 | 129 |
| Iodine (%) | 3.6 | 1.4, 10.0 | 2.1 (a) | 0.7, 6.4 | 0.0 (b) | 0.0, 5.7 | 7.1 (c) | 2.9, 13.6 | 5.0 (d) | 1.4, 10.7 | 513 |
| Selenium (%) | 5.0 | 1.77, 11.7 | 3.3 (a) | 1.7, 12.1 | 0.0 (b) | 0.0, 0.0 | 6.7 (a) | 0.0, 11.7 | 5.0 (a) | 1.7, 10.0 | 693 |
|  |  |  |  |  |  |  |  |  |  |  |  |
| Overall (%) (/18, Exc VitE, NA, CL) (n = 2978) | 11.3 | 6.8, 18.0 | 11.7 (a) | 7.2, 22.5 | 3.9 (b) | 0.1, 9.8 | 12.0 (ac) | 6.3, 21.7 | 11.2 (c) | 6.9, 16.4 | 25 |
| Overall (%) (/20, Inc NA, CL, Exc VItE) (n = 2978) | 11.7 | 7.1, 18.6 | 10.9 (a) | 6.8, 20.8 | 4.1 (b) | 0.1, 11.5 | 12.1 (a) | 6.4, 23.0 | 12.0 (a) | 7.5, 17.2 | 20 |

*Overall (%) (/18) includes all micronutrients except Sodium, Chloride and Vitamin E. Overall (%) (/20) includes all micronutrients except Vitamin E. Unlike letters indicate significantly different P < 0.05. Pairwise comparisons conducted using Kruskal–Wallis ANOVA with Bonferroni correction for multiple comparisons. Selenium: total = 2978 (UPF, n = 1648). IQR: inter-quartile range; MPF: minimally processed food; PCI: processed culinary ingredients; PF: processed food; UPF: ultra-processed food.*

**Table 4. Overall average absolute micronutrient content per 100g, and across each Nova group.**

| **Micronutrient** | **Total (n = 2980)** | | **MPF (n = 986)** | | **PCI (n = 61)** | | **PF (n = 283)** | | **UPF (n = 1650)** | | **Number of items with 0 micronutrient content per 100g** |
| --- | --- | --- | --- | --- | --- | --- | --- | --- | --- | --- | --- |
|  | **Median** | **IQR** | **Median** | **IQR** | **Median** | **IQR** | **Median** | **IQR** | **Median** | **IQR** |  |
| Vitamin A (retinol equivalents) (µg) | 13.0 | 0.0, 68.0 | 13.0 (a) | 0.0, 60.3 | 0.0 (b) | 0.0, 311.0 | 31.0 (c) | 3.0, 124.0 | 10.0 (b) | 0.0, 61.0 | 974 |
| Vitamin D (µg) | 0.0 | 0.0, 0.2 | 0.0 (a) | 0.0, 0.1 | 0.0 (a) | 0.0, 0.0 | 0.1 (b) | 0.0, 0.6 | 0.0 (c) | 0.0, 0.3 | 1758 |
| Thiamin (mg) | 0.1 | 0.0, 0.2 | 0.0 (a) | 0.0, 0.2 | 0.0 (b) | 0.0, 0.7 | 0.0 (c) | 0.0, 0.2 | 0.0 (a) | 0.0, 0.1 | 356 |
| Riboflavin (mg) | 0.1 | 0.0, 0.2 | 0.1 (a) | 0.0, 0.2 | 0.0 (b) | 0.0, 0.2 | 0.1 (c) | 0.0, 0.2 | 0.1 (ac) | 0.0, 0.2 | 308 |
| Niacin equivalent (mg) | 2.1 | 0.8, 4.9 | 1.5 (a) | 0.7, 5.5 | 0.0 (b) | 0.0, 0.4 | 2.5 (a) | 0.9, 5.6 | 2.4 (a) | 0.9, 4.8 | 196 |
| Vitamin C (mg) | 0.0 | 0.0, 4.6 | 2.0 (a) | 0.0,14.2 | 0.0 (b) | 0.0, 0.0 | 0.2 (c) | 0.0, 3.8 | 0.0 (d) | 0.0,1.8 | 1505 |
| Vitamin B6 (mg) | 0.1 | 0.0, 0.2 | 0.1 (a) | 0.1, 0.3 | 0.0 (b) | 0.0, 0.0 | 0.1 (c) | 0.1, 0.2 | 0.1 (c) | 0.0, 0.2 | 829 |
| Vitamin B12 (µg) | 0.0 | 0.0, 0.6 | 0.0 (a) | 0.0, 0.7 | 0.0 (a) | 0.0, 0.1 | 0.4 (b) | 0.0, 1.0 | 0.1 (c) | 0.0, 0.5 | 1607 |
| Folate (µg) B9 | 10.0 | 5.0, 24.0 | 12.0 (a) | 6.0, 29.8 | 0.0 (b) | 0.0, 1.0 | 11 (ac) | 5.0, 22.0 | 10 (c) | 4.0, 22.0 | 301 |
|  |  |  |  |  |  |  |  |  |  |  |  |
| Potassium (mg) | 200.0 | 124.0, 310.0 | 252.0 (a) | 166.0, 362.0 | 22.0 (b) | 0.0,102.0 | 159.0 (c) | 96.0, 255.0 | 186.0 (c) | 114.0, 270.3 | 49 |
| Calcium (mg) | 39.0 | 14.0, 100.0 | 23.0 (a) | 11.0, 55.3 | 7.0 (b) | 0.0, 58.0 | 49.0 (c) | 18.0, 113.0 | 53.5 (c) | 17.0, 110.0 | 46 |
| Magnesium (mg) | 19.0 | 11.0, 30.0 | 19.0 (a) | 11.0, 30.0 | 1.0 (b) | 0.0, 8.5 | 16.0 (c) | 11.0, 24.0 | 20.0 (ac) | 11.0, 32.0 | 74 |
| Phosphorus (mg) | 100.0 | 36.0, 179.0 | 86.0 (a) | 30.0, 180.0 | 3.0 (b) | 0.0, 55.0 | 118.0 (c) | 30.0, 206.0 | 112.0 (c) | 51.8, 177.0 | 82 |
| Iron (mg) | 0.9 | 0.3, 1.7 | 0.8 (a) | 0.3, 1.9 | 0.1 (b) | 0.0, 0.4 | 0.6 (c) | 0.3, 1.2 | 1.0 (a) | 0.4, 1.7 | 232 |
| Copper (mg) | 0.1 | 0.0, 0.2 | 0.07 (a) | 0.03, 0.14 | 0.01 (b) | 0.00, 0.07 | 0.07 (a) | 0.03, 0.11 | 0.10 (c) | 0.04, 0.18 | 395 |
| Zinc (mg) | 0.6 | 0.3, 1.2 | 0.5 (a) | 0.2, 1.5 | 0.1 (b) | 0.0, 0.3 | 0.7 (a) | 0.3, 1.5 | 0.7 (a) | 0.3, 1.2 | 291 |
| Sodium | 109.5 | 20.0, 342.0 | 26.5 (a) | 4.0, 80.0 | 11.0 (a) | 0.0, 49.5 | 224.0 (b) | 45.0, 418.0 | 230.0 (b) | 72.8, 426.3 | 99 |
| Chloride (mg) | 170.0 | 58.0, 515.0 | 73.0 (a) | 29.8, 143.3 | 18.0 (b) | 0.0, 78.5 | 331.0 (c) | 97.0, 618.0 | 337.0 (c) | 113.0, 661.0 | 129 |
| Iodine (µg) | 5.0 | 2.0, 14.0 | 3.0 (a) | 1.0, 9.0 | 0.0 (b) | 0.0, 8.0 | 10.0 (c) | 4.0, 19.0 | 7.0 (d) | 2.0, 15.0 | 513 |
| Selenium (µg) | 3.0 | 1.0, 7.0 | 2.0 (a) | 1.0, 7.3 | 0.0 (b) | 0.0, 0.0 | 4.0 (a) | 0.0, 7.0 | 3.0 (a) | 1.0, 6.0 | 693 |
|  |  |  |  |  |  |  |  |  |  |  |  |
| Retinol (µg) | 0.0 | 0.0, 24.0 | 0.0 (a) | 0.0, 2.0 | 0.0 (ab) | 0.0, 291.0 | 14.0 (c) | 0.0, 95.0 | 0.0 (b) | 0.0, 33.0 | 1803 |
| Total carotene (µg) | 10.0 | 0.0, 103.0 | 16.0 (a) | 0.0, 185.5 | 0.0 (b) | 0.0, 85.0 | 34.0 (a) | 0.0, 139.0 | 6.0 (b) | 0.0, 72.3 | 1182 |
| Alpha-carotene (µg) | 0.0 | 0.0, 0.0 | 0.0 (a) | 0.0, 0.0 | 0.0 (b) | 0.0, 0.0 | 0.0 (a) | 0.0, 0.0 | 0.0 (b) | 0.0, 0.0 | 2538 |
| Beta-carotene (µg) | 9.0 | 0.0, 98.0 | 13.0 (a) | 0.0, 177.0 | 0.0 (b) | 0.0, 85.0 | 33.0 (a) | 0.0, 133.0 | 6.0 (b) | 0.0, 70.3 | 1186 |
| Beta cryptoxanthin (µg) | 0.0 | 0.0, 0.0 | 0.0 (a) | 0.0, 0.0 | 0.0 (b) | 0.0, 0.0 | 0.0 (ac) | 0.0, 0.0 | 0.0 (c) | 0.0, 0.0 | 2540 |
| Vitamin E (mg) | 0.5 | 0.1, 1.5 | 0.4 (a) | 0.1, 1.0 | 0.5 (ab) | 0.0, 3.5 | 0.5 (ab) | 0.2, 1.3 | 0.7 (b) | 0.2, 1.8 | 484 |
| Pantothenic acid (mg) | 0.4 | 0.2, 0.6 | 0.4 (a) | 0.2, 0.7 | 0.0 (b) | 0.0, 0.0 | 0.4 (a) | 0.2, 0.6 | 0.4 (a) | 0.2, 0.6 | 379 |
| Biotin (µg) | 2.0 | 1.0, 4.0 | 1.0 (a) | 0.0, 3.0 | 0.0 (b) | 0.0, 0.0 | 2.0 (c) | 1.0, 4.0 | 2.0 (c) | 1.0, 4.0 | 704 |
| Haem iron (mg) | 0.0 | 0.0, 0.0 | 0.0 (a) | 0.0, 0.0 | 0.0 (b) | 0.0, 0.0 | 0.0 (ac) | 0.0, 0.0 | 0.0 (c) | 0.0, 0.0 | 2321 |
| Non-haem iron (mg) | 0.7 | 0.3, 1.6 | 0.7 (a) | 0.3, 1.6 | 0.1 (b) | 0.0, 0.4 | 0.5 (c) | 0.3, 1.1 | 0.9 (a) | 0.4, 1.6 | 234 |
| Manganese (mg) | 0.2 | 0.0, 0.4 | 0.1 (a) | 0.0, 0.4 | 0.0 (b) | 0.0, 0.0 | 0.1 (c) | 0.0, 0.2 | 0.2 (d) | 0.0, 0.4 | 451 |

*Unlike letters indicate significantly different P < 0.05. Pairwise comparisons conducted using Kruskal–Wallis ANOVA with Bonferroni correction for multiple comparisons. IQR: inter-quartile range; MPF: minimally processed food; PCI: processed culinary ingredients; PF: processed food; UPF: ultra-processed food.*

**Table 5. Average percentage contribution per 100g to UK government dietary micronutrient recommendations from the UK NDNS for males aged 19-64** **years, and across each Nova group.**

| **Micronutrient** | **Total (n = 2980)** | | **MPF (n = 986)** | | **PCI (n = 61)** | | **PF (n = 283)** | | **UPF (n = 1650)** | |
| --- | --- | --- | --- | --- | --- | --- | --- | --- | --- | --- |
|  | **Median** | **IQR** | **Median** | **IQR** | **Median** | **IQR** | **Median** | **IQR** | **Median** | **IQR** |
| Vitamin A (retinol equivalents) (%) | 1.8 | 0.0, 9.7 | 1.9 (a) | 0.0, 8.6 | 0.0 (b) | 0.0, 44.4 | 4.4 (c) | 0.4, 17.7 | 1.4 (b) | 0.0, 8.7 |
| Vitamin D (%) | 0.0 | 0.0, 2.4 | 0.0 (a) | 0.0, 1.0 | 0.0 (a) | 0.0, 0.4 | 1.2 (b) | 0.0, 6.0 | 0.0 (c) | 0.0, 2.5 |
| Thiamin (%) | 9.0 | 3.0, 17.0 | 9.0 (a) | 4.0, 15.3 | 0.0 (b) | 0.0, 1.0 | 5.0 (c) | 2.0, 11.0 | 9.5 (a) | 3.0, 19.0 |
| Riboflavin (%) | 6.9 | 2.3, 13.8 | 5.4 (a) | 2.3, 14.6 | 0.0 (b) | 0.0, 12.7 | 8.5 (c) | 2.3, 16.9 | 7.7 (ac) | 3.1, 13.8 |
| Niacin equivalent (%) | 12.4 | 4.7, 28.8 | 8.8 (a) | 4.1, 32.5 | 0.0 (b) | 0.0, 2.1 | 14.7 (a) | 5.3, 32.9 | 14.1 (a) | 5.3, 28.2 |
| Vitamin C (%) | 0.0 | 0.0, 11.5 | 5.0 (a) | 0.0, 35.4 | 0.0 (b) | 0.0, 0.0 | 0.5 (c) | 0.0, 9.5 | 0.0 (d) | 0.0, 4.6 |
| Vitamin E (%) | 12.5 | 2.5, 37.5 | 10.0 (a) | 2.5, 25.6 | 12.5 (ab) | 0.0, 87.5 | 12.5 (ab) | 5.0, 32.5 | 17.5 (b) | 5.0, 45.0 |
| Vitamin B6 (%) | 7.1 | 0.0, 14.3 | 7.1 (a) | 7.1, 21.4 | 0.0 (a) | 0.0, 0.0 | 7.1 (b) | 7.1, 14.3 | 7.1 (c) | 0.0, 14.3 |
| Vitamin B12 (%) | 0.0 | 0.0, 40.0 | 0.0 (a) | 0.0, 46.7 | 0.0 (a) | 0.0, 6.7 | 26.7 (b) | 0.0, 66.7 | 6.7 (c) | 0.0, 33.3 |
| Folate (%) | 5.0 | 2.5, 12.0 | 6.0 (a) | 3.0, 15.0 | 0.0 (b) | 0.0, 0.5 | 5.5 (ac) | 2.5, 11.0 | 5.0 (c) | 2.0, 11.1 |
|  |  |  |  |  |  |  |  |  |  |  |
| Potassium (%) | 5.7 | 3.5, 8.9 | 7.2 (a) | 4.7, 10.3 | 0.6 (b) | 0.0, 2.9 | 4.5 (c) | 2.7, 7.3 | 5.3 (c) | 3.3, 7.7 |
| Calcium (%) | 5.6 | 2.0, 14.3 | 3.3 (a) | 1.6, 7.9 | 1.0 (b) | 0.0, 8.3 | 7.0 (c) | 2.6, 16.1 | 7.6 (c) | 2.4, 15.7 |
| Magnesium (%) | 6.3 | 3.7, 10.0 | 6.3 (a) | 3.7, 10.0 | 0.3 (b) | 0.0, 2.8 | 5.3 (c) | 3.7, 8.0 | 6.7 (ac) | 3.7, 10.7 |
| Phosphorus (%) | 18.2 | 6.6, 32.6 | 15.6 (a) | 5.5, 32.7 | 0.6 (b) | 0.0, 10.0 | 21.5 (c) | 5.5, 37.5 | 20.4 (c) | 9.4, 32.2 |
| Iron (%) | 10.3 | 3.4, 19.5 | 9.2 (a) | 3.4, 21.8 | 1.1 (b) | 0.0, 4.6 | 6.9 (c) | 3.4, 13.8 | 11.5 (a) | 4.6, 19.5 |
| Copper (%) | 6.7 | 2.5, 13.3 | 5.8 (a) | 2.5, 11.7 | 0.8 (b) | 0.0, 5.4 | 5.8 (a) | 2.5, 9.2 | 8.3 (c) | 3.33, 15.0 |
| Zinc (%) | 6.3 | 3.2, 12.6 | 5.3 (a) | 2.1, 15.8 | 1.1 (b) | 0.0, 3.2 | 7.4 (a) | 3.2, 15.8 | 7.4 (a) | 3.2, 12.6 |
| Sodium (%) | 6.8 | 1.3, 21.4 | 1.7 (a) | 0.3, 5.0 | 0.7 (a) | 0.0, 3.1 | 14.0 (b) | 2.8, 26.1 | 14.4 (b) | 4.5, 26.6 |
| Chloride (%) | 6.8 | 2.3, 20.6 | 2.9 (a) | 1.2, 5.7 | 0.7 (b) | 0.0, 3.1 | 13.2 (c) | 3.9, 24.7 | 13.5 (c) | 4.5, 26.4 |
| Iodine (%) | 3.6 | 1.4, 10.0 | 2.1 (a) | 0.7, 6.4 | 0.0 (b) | 0.0, 5.7 | 7.1 (c) | 2.9, 13.6 | 5.0 (d) | 1.4, 10.7 |
| Selenium (%) | 4.0 | 1.3, 9.3 | 2.7 (a) | 1.3, 9.7 | 0.0 (b) | 0.0, 0.0 | 5.3 (a) | 0.0, 9.3 | 4.0 (a) | 1.3, 8.0 |
|  |  |  |  |  |  |  |  |  |  |  |
| Overall (%) (/18, Exc VitE, NA, CL) (n = 2978) | 10.7 | 6.5, 16.7 | 11.1 (a) | 6.9, 21.0 | 3.9 (b) | 0.2, 9.0 | 11.2 (ac) | 5.9, 20.5 | 10.5 (c) | 6.5, 15.2 |
| Overall (%) (/20, Inc NA, CL, Exc VItE) (n = 2978) | 11.0 | 6.7, 17.4 | 10.3 (a) | 6.6, 19.5 | 4.6 (b) | 0.2, 10.4 | 11.5 (a) | 6.2, 21.0 | 11.5 (a) | 7.1, 16.4 |

*Overall (%) (/18) includes all micronutrients except Sodium, Chloride and Vitamin E. Overall (%) (/20) includes all micronutrients except Vitamin E. Unlike letters indicate significantly different P < 0.05. Pairwise comparisons conducted using Kruskal–Wallis ANOVA with Bonferroni correction for multiple comparisons. Selenium: total = 2978 (UPF, n = 1648). IQR: inter-quartile range; MPF: minimally processed food; PCI: processed culinary ingredients; PF: processed food; UPF: ultra-processed food.*

**Table 6. Overall average absolute micronutrient content per 100kcal, and across each Nova group.**

| **Micronutrient** | **Total (n = 2955)** | | **MPF (n = 978)** | | **PCI (n = 57)** | | **PF (n = 283)** | | **UPF (n = 1637)** | | **Number of items with 0 content of micronutrient per 100kcal** |
| --- | --- | --- | --- | --- | --- | --- | --- | --- | --- | --- | --- |
|  | **Median** | **IQR** | **Median** | **IQR** | **Median** | **IQR** | **Median** | **IQR** | **Median** | **IQR** |  |
| Vitamin A (retinol equivalents) (µg) | 8.3 | 0.0, 50.0 | 14.4 (a) | 0.2, 95.6 | 0.0 (b) | 0, 124.97 | 26.9 (a) | 3.2, 64.1 | 5.3 (b) | 0.0, 31.8 | 949 |
| Vitamin D (µg) | 0.0 | 0.0, 0.1 | 0.0 (a) | 0.0, 0.1 | 0.0 (a) | 0.0, 0.1 | 0.0 (b) | 0.1, 0.3 | 0.0 (c) | 0.0, 0.1 | 1733 |
| Thiamin (mg) | 0.06 | 0.02, 0.12 | 0.10 (a) | 0.05, 0.20 | 0.0 (b) | 0.0, 0.01 | 0.03 (c) | 0.01, 0.06 | 0.04 (d) | 0.02, 0.10 | 331 |
| Riboflavin (mg) | 0.06 | 0.02, 0.12 | 0.09 (a) | 0.04, 0.18 | 0.0 (b) | 0.0, 0.08 | 0.07 (c) | 0.03, 0.11 | 0.04 (d) | 0.02, 0.09 | 287 |
| Niacin equivalent (mg) | 1.5 | 0.6, 2.9 | 2.3 (a) | 1.3, 4.3 | 0.0 (b) | 0.0, 0.0 | 1.5 (c) | 0.7, 2.6 | 1.1 (d) | 0.5, 2.3 | 173 |
| Vitamin C (mg) | 0.0 | 0.0, 4.5 | 2.9 (a) | 0.0, 25.8 | 0.0 (b) | 0.0, 0.0 | 0.6 (c) | 0.0, 2.4 | 0.0 (d) | 0.0, 1.0 | 1481 |
| Vitamin B6 (mg) | 0.07 | 0.00, 0.16 | 0.14 (a) | 0.08, 0.30 | 0.0 (b) | 0.0, 0.0 | 0.06 (c) | 0.02, 0.11 | 0.04 (d) | 0.0, 0.10 | 805 |
| Vitamin B12 (µg) | 0.0 | 0.0, 0.3 | 0.0 (a) | 0.0, 0.6 | 0.0 (b) | 0.0, 0.0 | 0.2 (c) | 0.0, 0.5 | 0.0 (a) | 0.0, 0.3 | 1583 |
| Folate (µg) B9 | 7.1 | 2.6, 17.8 | 13.6 (a) | 5.9, 46.2 | 0.0 (b) | 0.0, 1.6 | 6.8 (c) | 2.9, 14.9 | 5.2 (d) | 1.9, 11.8 | 279 |
|  |  |  |  |  |  |  |  |  |  |  |  |
| Potassium (mg) | 124.3 | 62.8, 278.7 | 316.9 (a) | 177.4, 523.7 | 3.7 (b) | 0.0, 53.9 | 112.7 (c) | 55.0, 190.5 | 86.4 (d) | 50.8, 147.4 | 36 |
| Calcium (mg) | 26.4 | 11.5, 65.4 | 29.5 (a) | 11.3, 100.0 | 2.4 (b) | 0.0, 45.7 | 29.8 (a) | 15.0, 75.6 | 25.0 (c) | 11.5, 52.8 | 31 |
| Magnesium (mg) | 12.9 | 7.7, 25.0 | 24.4 (a) | 14.0, 41.7 | 0.3 (b) | 0.0, 5.2 | 10.6 (c) | 7.0, 18.0 | 10.1 (c) | 6.0, 17.0 | 59 |
| Phosphorus (mg) | 64.3 | 32.7, 108.3 | 100.0 (a) | 54.3, 164.3 | 1.2 (b) | 0.0, 40.2 | 68.1 (c) | 31.9, 110.5 | 52.0 (d) | 27.5, 81.3 | 61 |
| Iron (mg) | 0.5 | 0.3, 1.1 | 1.0 (a) | 0.4, 2.0 | 0 (b) | 0.0, 0.1 | 0.4 (c) | 0.2, 0.8 | 0.4 (c) | 0.2, 0.8 | 210 |
| Copper (mg) | 0.05 | 0.03, 0.10 | 0.09 (a) | 0.04, 0.19 | 0.0 (b) | 0.0, 0.02 | 0.04 (c) | 0.02, 0.09 | 0.05 (c) | 0.02, 0.08 | 376 |
| Zinc (mg) | 0.4 | 0.2, 0.8 | 0.8 (a) | 0.4, 1.3 | 0.0 (b) | 0.0, 0.2 | 0.4 (c) | 0.2, 0.8 | 0.3 (d) | 0.1, 0.6 | 266 |
| Sodium | 72.4 | 19.9, 166.0 | 31.7 (a) | 5.3, 93.3 | 3.1 (b) | 0.0, 21.9 | 96.5 (c) | 33.5, 187.8 | 108.8 (c) | 42.2, 201.2 | 85 |
| Chloride (mg) | 125.0 | 40.4, 272.4 | 88.5 (a) | 31.3, 204.8 | 2.4 (b) | 0.0, 36.1 | 152.9 (c) | 68.5, 274.3 | 152.8 (c) | 50.4, 311.7 | 115 |
| Iodine (µg) | 3.5 | 1.1, 8.3 | 4.3 (a) | 1.8, 11.0 | 0.0 (b) | 0.0, 2.2 | 6.2 (a) | 2.3, 11.8 | 2.9 (c) | 0.8, 6.5 | 493 |
| Selenium (µg) | 1.5 | 0.3, 3.9 | 2.6 (a) | 0.4, 6.3 | 0.0 (b) | 0.0, 0.0 | 1.4 (c) | 0.0, 3.6 | 1.3 (c) | 0.4, 3.0 | 668 |
|  |  |  |  |  |  |  |  |  |  |  |  |
| Retinol (µg) | 0.0 | 0.0, 13.1 | 0.0 (a) | 0.0, 1.8 | 0.0 (ac) | 0.0, 104.8 | 9.6 (b) | 0.0, 44.9 | 0.0 (c) | 0.0, 15.4 | 1778 |
| Total carotene (µg) | 6.3 | 0.0, 63.2 | 24.1 (a) | 0.0, 358.6 | 0.0 (b) | 0.0, 37.2 | 24.1 (a) | 0.0, 58.1 | 3.1 (b) | 0.0, 30.5 | 1157 |
| Alpha-carotene (µg) | 0.0 | 0.0, 0.0 | 0.0 (a) | 0.0, 0.0 | 0.0 (b) | 0.0, 0.0 | 0.0 (a) | 0.0, 0.0 | 0.0 (b) | 0.0, 0.0 | 2513 |
| Beta-carotene (µg) | 6.0 | 0.0, 59.4 | 22.4 (a) | 0.0, 329.6 | 0.0 (b) | 0.0, 37.2 | 23.9 (a) | 0.0, 58.1 | 3.0 (b) | 0.0, 29.1 | 1161 |
| Beta cryptoxanthin (µg) | 0.0 | 0.0, 0.0 | 0.0 (a) | 0.0, 0.0 | 0.0 (b) | 0.0, 0.0 | 0.0 (ac) | 0.0, 0.0 | 0.0 (bc) | 0.0, 0.0 | 2515 |
| Vitamin E (mg) | 0.3 | 0.1, 0.9 | 0.5 (a) | 0.1, 1.4 | 0.2 (b) | 0.0, 0.6 | 0.3 (b) | 0.1, 0.8 | 0.3 (b) | 0.1, 0.7 | 459 |
| Pantothenic acid (mg) | 0.2 | 0.1, 0.5 | 0.5 (a) | 0.2, 0.8 | 0.0 (b) | 0.0, 0.1 | 0.2 (c) | 0.1, 0.4 | 0.2 (d) | 0.1, 0.3 | 355 |
| Biotin (µg) | 1.0 | 0.3, 2.3 | 1.4 (a) | 0.0, 3.8 | 0.0 (b) | 0.0, 0.1 | 1.2 (a) | 0.7, 2.4 | 0.9 (c) | 0.4, 2.0 | 679 |
| Haem iron (mg) | 0.0 | 0.0, 0.0 | 0.0 (a) | 0.0, 0.0 | 0.0 (b) | 0.0, 0.0 | 0.0 (ac) | 0.0, 0.0 | 0.0 (c) | 0.0, 0.0 | 2296 |
| Non-haem iron (mg) | 0.5 | 0.2, 1.0 | 0.8 (a) | 0.4, 2.0 | 0.0 (b) | 0.0, 0.1 | 0.4 (c) | 0.2, 0.7 | 0.4 (c) | 0.2, 0.7 | 212 |
| Manganese (mg) | 0.1 | 0.0, 0.3 | 0.2 (a) | 0.0, 0.6 | 0.0 (b) | 0.0, 0.0 | 0.1 (c) | 0.0, 0.1 | 0.1 (d) | 0.0, 0.2 | 431 |

*Unlike letters indicate significantly different P < 0.05. Pairwise comparisons conducted using Kruskal–Wallis ANOVA with Bonferroni correction for multiple comparisons. IQR: inter-quartile range; MPF: minimally processed food; PCI: processed culinary ingredients; PF: processed food; UPF: ultra-processed food.*

**Table 7. Average percentage contribution per 100kcal to UK government dietary micronutrient recommendations from the UK NDNS for males aged 19-64 years, and across each Nova group,** and percentage of government dietary micronutrient recommendations consumed per 100 kcal of reported energy intake for males, aged 19-64 from the NDNS Year 9-11 survey.

| **Micronutrient** | **Total (n = 2955)** | | **MPF (n = 978)** | | **PCI (n = 57)** | | **PF (n = 283)** | | **UPF (n = 1637)** | |  | **Percentage of RNI consumed per 100 kcal of reported energy intake from NDNS Years 9-11** | |
| --- | --- | --- | --- | --- | --- | --- | --- | --- | --- | --- | --- | --- | --- |
|  | **Median** | **IQR** | **Median** | **IQR** | **Median** | **IQR** | **Median** | **IQR** | **Median** | **IQR** |  | **Median (%)** | **IQR** |
| Vitamin A (retinol equivalents) (%) | 1.2 | 0.0, 7.1 | 2.1 (a) | 0.0, 13.7 | 0.0 (b) | 0.0, 17.9 | 3.9 (a) | 0.5, 9.2 | 0.8 (b) | 0.0, 4.5 |  | 4.8 | 3.4, 7.0 |
| Vitamin D (%) | 0.0 | 0, 1.242 | 0.0 (a) | 0, 0.875 | 0.0 (a) | 0.0, 0.5 | 0.7 (b) | 0.0, 2.5 | 0.0 (c) | 0.0, 1.2 |  | 1.3 | 0.8, 2.0 |
| Thiamin (%) | 5.7 | 2.3, 12.3 | 9.7 (a) | 5.3, 20.0 | 0.0 (b) | 0.0, 0.5 | 3.3 (c) | 1.3, 6.4 | 4.4 (d) | 1.6, 10.0 |  | 8.20 | 6.7, 9.9 |
| Riboflavin (%) | 4.4 | 1.7, 9.1 | 6.9 (a) | 3.2, 13.6 | 0.0 (b) | 0.0, 6.0 | 5.0 (c) | 2.2, 8.5 | 3.4 (d) | 1.2, 6.9 |  | 6.2 | 5.0, 7.6 |
| Niacin equivalent (%) | 8.9 | 3.5, 17.1 | 13.2 (a) | 7.3, 25.5 | 0.0 (b) | 0.0, 0.6 | 8.8 (c) | 4.3, 15.2 | 6.7 (d) | 2.7, 13.3 |  | 11.7 | 10.0, 13.8 |
| Vitamin C (%) | 0.0 | 0.0, 11.2 | 7.3 (a) | 0.0, 64.5 | 0.0 (b) | 0.0, 0.0 | 0.1 (c) | 0.0, 6.0 | 0.0 (d) | 0.0, 2.5 |  | 9.1 | 5.8, 13.8 |
| Vitamin E (%) | 11.2 | 3.3, 31.0 | 17.3 (a) | 4.6, 47.6 | 6.5 (b) | 0.0, 18.9 | 10.7 (b) | 4.0, 27.1 | 10.5 (b) | 2.8, 24.7 |  |  |  |
| Vitamin B6 (%) | 4.9 | 0.0, 11.1 | 10.2 (a) | 5.3, 21.3 | 0.0 (b) | 0.0, 0.0 | 3.9 (c) | 1.7, 8.1 | 2.9 (d) | 0.0, 7.1 |  | 6.8 | 5.7, 8.4 |
| Vitamin B12 (%) | 0 | 0.0, 22.5 | 0.0 (a) | 0.0, 38.2 | 0.0 (b) | 0.0, 1.9 | 10.3 (c) | 0.0, 35.8 | 1.6 (a) | 0.0, 18.3 |  | 16.5 | 12.2, 21.5 |
| Folate (%) | 3.6 | 1.3, 8.9 | 6.8 (a) | 2.9, 23.1 | 0.0 (b) | 0.0, 0.8 | 3.4 (c) | 1.5, 7.2 | 2.6 (d) | 1.0, 5.9 |  | 6.2 | 5.0, 7.8 |
|  |  |  |  |  |  |  |  |  |  |  |  |  |  |
| Potassium (%) | 3.6 | 1.8, 8.0 | 9.1 (a) | 5.1, 15.0 | 0.1 (b) | 0.0, 1.5 | 3.2 (c) | 1.6, 5.4 | 2.5 (d) | 1.5, 4.2 |  | 4.3 | 3.7, 4.9 |
| Calcium (%) | 3.8 | 1.6, 9.3 | 4.2 (a) | 1.6, 14.3 | 0.3 (b) | 0.0, 6.5 | 4.3 (a) | 2.1, 10.8 | 3.6 (c) | 1.6, 7.5 |  | 6.0 | 4.9, 7.4 |
| Magnesium (%) | 4.3 | 2.6, 8.3 | 8.1 (a) | 4.7, 13.9 | 0.1 (b) | 0.0, 1.7 | 3.5 (c) | 2.3, 6.0 | 3.4 (c) | 2.0, 5.7 |  | 4.8 | 4.2, 5.6 |
| Phosphorus (%) | 11.7 | 6.0, 19.7 | 18.2 (a) | 9.9, 29.9 | 0.2 (b) | 0.0, 7.3 | 12.4 (c) | 5.8, 20.1 | 9.5 (d) | 5.0, 14.8 |  | 12.0 | 10.9, 13.6 |
| Iron (%) | 5.9 | 3.1, 12.5 | 11.2 (a) | 5.1, 23.0 | 0.1 (b) | 0.0, 1.6 | 4.7 (c) | 2.3, 9.0 | 4.8 (c) | 2.6, 8.8 |  | 6.3 | 5.3, 7.6 |
| Copper (%) | 4.4 | 2.2, 8.3 | 7.4 (a) | 3.5, 15.5 | 0.1 (b) | 0.0, 1.3 | 3.5 (c) | 1.7, 7.6 | 3.8 (c) | 1.9, 6.3 |  | 5.1 | 4.3, 6.0 |
| Zinc (%) | 4.2 | 2.0, 8.8 | 8.0 (a) | 3.7, 14.0 | 0.3 (b) | 0.0, 1.8 | 4.5 (c) | 2.0, 8.5 | 3.0 (d) | 1.5, 5.9 |  | 4.7 | 4.1, 5.7 |
| Sodium (%) | 4.5 | 1.2, 10.4 | 2.0 (a) | 0.3, 5.8 | 0.2 (b) | 0.0, 1.4 | 6.0 (c) | 2.1, 11.7 | 6.8 (c) | 2.6, 12.6 |  | 7.0 | 6.0, 8.1 |
| Chloride (%) | 5.0 | 1.6, 10.9 | 3.5 (a) | 1.3, 8.2 | 0.1 (b) | 0.0, 1.4 | 6.1 (c) | 2.7, 11.0 | 6.1 (c) | 2.0, 12.5 |  | 7.5 | 6.4, 8.7 |
| Iodine (%) | 2.5 | 0.8, 6.0 | 3.1 (a) | 1.3, 7.9 | 0.0 (b) | 0.0, 1.6 | 4.4 (c) | 1.7, 8.4 | 2.0 (c) | 0.6, 4.6 |  | 5.4 | 4.1, 7.2 |
| Selenium (%) | 2.1 | 0.4, 5.2 | 3.5 (a) | 0.5, 8.4 | 0.0 (b) | 0.0, 0.0 | 1.9 (c) | 0.0, 4.7 | 1.7 (c) | 0.5, 4.0 |  | 3.4 | 2.7, 4.3 |
|  |  |  |  |  |  |  |  |  |  |  |  |  |  |
| Overall (%) (/18, Exc VitE, NA, CL) (n = 2953) | 7.1 | 3.8, 13.3 | 13.2 (a) | 7.8, 26.8 | 0.8 (b) | 0.0, 4.6 | 7.1 (c) | 4.4, 10.1 | 5.4 (d) | 2.9, 9.1 |  | 7.1 | 6.2, 8.2 |
| Overall (%) (/20, Inc NA, CL, Exc VItE) (n = 2953) | 7.4 | 4.0, 13.1 | 12.3 (a) | 7.3, 25.3 | 0.7 (b) | 0.0, 4.3 | 7.1 (c) | 4.5, 10.6 | 5.9 (d) | 3.1, 9.7 |  | 7.1 | 6.3, 8.1 |

*Overall (%) (/18) includes all micronutrients except Sodium, Chloride and Vitamin E. Overall (%) (/20) includes all micronutrients except Vitamin E. Unlike letters indicate significantly different P < 0.05. Pairwise comparisons conducted using Kruskal–Wallis ANOVA with Bonferroni correction for multiple comparisons. Selenium: total = 2953 (UPF, n = 1635). IQR: inter-quartile range; MPF: minimally processed food; PCI: processed culinary ingredients; PF: processed food; UPF: ultra-processed food. NA, Sodium; CL, Chloride Exc., Excluded, Inc, Included. %, percentage; n, number, RNI, Reference Nutrient Intake, NDNS. National Diet and Nutrition Survey*

**Table 8. Quartiles of average percentage contribution to daily dietary recommendations for females aged 19-64 years of food and drink items per 100g and 100kcal, by Nova group and by healthy vs. unhealthy FOPL.**

| **Overall (%) (/18) /100g % (n = 2978)** | **MPF** | | | **PCI** | | | **PF** | | | **UPF** | | | **p-value** |  | **Healthy FOPL** | | | **Unhealthy FOPL** | | | **p-value** |
| --- | --- | --- | --- | --- | --- | --- | --- | --- | --- | --- | --- | --- | --- | --- | --- | --- | --- | --- | --- | --- | --- |
|  | **N** | **% quartile** | **% MPF** | **N** | **% quartile** | **% PCI** | **N** | **% quartile** | **% PF** | **N** | **% quartile** | **% UPF** |  |  | **N** | **% quartile** | **% healthy** | **N** | **% quartile** | **% unhealthy** |  |
| Q1 (743) | 222 | 29.9 | 22.5 | 38 | 5.1 | 62.3 | 76 | 10.2 | 26.9 | 407 | 54.8 | 24.7 | <0.001 |  | 517 | 69.6 | 28.0 | 226 | 30.4 | 19.9 | <0.001 |
| Q2 (746) | 257 | 34.5 | 26.1 | 10 | 1.3 | 16.4 | 54 | 7.2 | 19.1 | 425 | 57.0 | 25.8 |  |  | 491 | 65.8 | 26.6 | 255 | 34.2 | 22.5 |  |
| Q3 (741) | 177 | 23.9 | 18.0 | 8 | 1.1 | 13.1 | 62 | 8.4 | 21.9 | 494 | 66.7 | 30.0 |  |  | 467 | 63.0 | 25.3 | 274 | 37.0 | 24.2 |  |
| Q4 (748) | 330 | 44.1 | 33.5 | 5 | 0.7 | 8.2 | 91 | 12.2 | 32.2 | 322 | 43.0 | 19.5 |  |  | 370 | 49.5 | 20.1 | 378 | 50.5 | 33.4 |  |
| **Overall (%) (/18) /100kcal (n = 2953)** | **MPF** | | | **PCI** | | | **PF** | | | **UPF** | | | **p-value** |  | **Healthy FOPL** | | | **Unhealthy FOPL** | | | **p-value** |
|  | **N** | **% quartile** | **% MPF** | **N** | **% quartile** | **% PCI** | **N** | **% quartile** | **% PF** | **N** | **% quartile** | **% UPF** |  |  | **N** | **% quartile** | **% healthy** | **N** | **% quartile** | **% unhealthy** |  |
| Q1 (738) | 60 | 8.1 | 6.1 | 41 | 5.6 | 71.9 | 61 | 8.3 | 21.6 | 576 | 78.0 | 35.2 | <0.001 |  | 206 | 27.9 | 11.3 | 532 | 72.1 | 47.1 | <0.001 |
| Q2 (739) | 144 | 19.5 | 14.7 | 7 | 0.9 | 12.3 | 82 | 11.1 | 29.0 | 506 | 68.5 | 30.9 |  |  | 484 | 65.5 | 26.5 | 255 | 34.5 | 22.6 |  |
| Q3 (738) | 285 | 38.6 | 29.1 | 2 | 0.3 | 3.5 | 97 | 13.1 | 34.3 | 354 | 48.0 | 21.7 |  |  | 518 | 70.2 | 28.4 | 220 | 29.8 | 19.5 |  |
| Q4 (738) | 489 | 66.3 | 50.0 | 7 | 0.9 | 12.3 | 43 | 5.8 | 15.2 | 199 | 27.0 | 12.2 |  |  | 616 | 83.5 | 33.8 | 122 | 16.5 | 10.8 |  |

*Overall (%) (/18) includes all micronutrients except Sodium, Chloride and Vitamin E. Overall (%) (/20) includes all micronutrients except Vitamin E. Items were classified into ‘healthy’ or ‘unhealthy’ based on the presence or absence of a red FOPL traffic light for fat, saturated fat, total sugar or salt. This is based on research that when identifying healthier products, UK consumers are more cautious to avoid items with red traffic lights, than to select items with green traffic lights. FOPL, front of package label ;IQR: inter-quartile range; MPF: minimally processed food; PCI: processed culinary ingredients; PF: processed food; UPF: ultra-processed food.*

**Table 9. Average percentage contribution of healthy items per 100g to UK government dietary micronutrient recommendations from the UK NDNS for females aged 19-64 years, and across each Nova group.**

| **Micronutrient** | **Total (n = 1846)** | | **MPF (n = 820)** | | **PCI (n = 9)** | | **PF (n = 162)** | | **UPF (n = 855)** | | **Number of items with 0 content of micronutrient per 100g** |
| --- | --- | --- | --- | --- | --- | --- | --- | --- | --- | --- | --- |
|  | **Median** | **IQR** | **Median** | **IQR** | **Median** | **IQR** | **Median** | **IQR** | **Median** | **IQR** |  |
| Vitamin A (retinol equivalents) (%) | 1.5 | 0.0, 8.7 | 2.0 (a) | 0.0, 9.7 | 0.0 (b) | 0.0, 0.0 | 2.3 (a) | 0.3, 10.7 | 1.0 (c) | 0.0, 7.3 | 593 |
| Vitamin D (%) | 0.0 | 0.0, 1.6 | 0.0 (a) | 0.0, 0.7 | 0.0 (a) | 0.0, 0.0 | 0.2 (b) | 0.0, 4.0 | 0.0 (b) | 0.0, 1.7 | 1139 |
| Thiamin (%) | 10.0 | 3.8, 20.0 | 10.0 (a) | 5.0, 17.2 | 1.3 (ab) | 0.0, 45.0 | 5.0 (b) | 1.3, 11.3 | 12.5 (c) | 5.0, 26.3 | 174 |
| Riboflavin (%) | 6.4 | 1.8, 13.6 | 5.5 (a) | 1.8, 13.6 | 5.5 (a) | 0.0, 80.0 | 5.9 (a) | 1.8, 12.7 | 7.3 (a) | 2.7, 13.6 | 178 |
| Niacin equivalent (%) | 14.6 | 5.4, 36.2 | 10.0 (a) | 5.4, 35.8 | 0.0 (a) | 0.0, 50.0 | 11.5 (a) | 3.1, 27.9 | 20.0 (b) | 6.2, 36.9 | 85 |
| Vitamin C (%) | 2.5 | 0.0, 20.0 | 7.3 (a) | 0.0, 37.5 | 0.0 (b) | 0.0, 0.0 | 2.6 (c) | 0.0, 16.3 | 0.3 (b) | 0.0, 9.8 | 753 |
| Vitamin E (%) | 13.3 | 3.3, 36.7 | 10.0 (a) | 3.3, 30.00 | 0.0 (b) | 0.0, 0.0 | 13.3 (ac) | 3.3, 30.8 | 16.7 (c) | 3.3, 50.0 | 327 |
| Vitamin B6 (%) | 8.3 | 8.3, 16.7 | 8.3 (a) | 8.3, 16.7 | 0.0 (ab) | 0.0, 25.0 | 8.3 (b) | 0.0, 16.7 | 8.3 (b) | 0.0, 16.7 | 458 |
| Vitamin B12 (%) | 0.0 | 0.0, 33.3 | 0.0 (a) | 0.0, 40.0 | 0.0 (ac) | 0.0, 0.0 | 6.7 (b) | 0.0, 46.7 | 0.0 (bc) | 0.0, 26.7 | 1081 |
| Folate (%) | 6.0 | 2.9, 13.0 | 6.0 (a) | 3.5, 14.0 | 0.5 (bc) | 0.0, 312.8 | 4.5 (b) | 2.0, 8.0 | 6.0 (ac) | 2.5, 13.5 | 125 |
|  |  |  |  |  |  |  |  |  |  |  |  |
| Potassium (%) | 5.7 | 3.7, 8.3 | 6.5 (a) | 4.6, 9.6 | 0.2 (b) | 0.1, 9.2 | 4.7 (b) | 2.7, 6.6 | 5.2 (b) | 3.3, 7.2 | 25 |
| Calcium (%) | 4.4 | 1.7, 12.1 | 3.1 (ab) | 1.6, 6.3 | 1.0 (a) | 0.2, 2.6 | 4.3 (b) | 1.6, 11.3 | 7.1 (c) | 2.4, 15.7 | 24 |
| Magnesium (%) | 6.7 | 3.7, 10.0 | 6.7 (a) | 3.7, 10.0 | 0.4 (b) | 0.0, 11.7 | 4.8 (b) | 3.0, 7.8 | 7.0 (a) | 4.1, 10.0 | 31 |
| Phosphorus (%) | 16.0 | 5.5, 27.8 | 12.4 (a) | 5.1, 27.5 | 0.4 (a) | 0.0, 36.4 | 13.5 (a) | 3.0, 26.6 | 18.5 (b) | 8.5, 28.4 | 45 |
| Iron (%) | 4.7 | 2.0, 10.1 | 4.7 (a) | 2.0, 10.8 | 18.2 (a) | 0.0, 26.0 | 4.0 (a) | 2.0, 7.6 | 5.4 (a) | 2.0, 10.8 | 149 |
| Copper (%) | 5.8 | 2.5, 10.8 | 5.0 (ab) | 2.5, 9.2 | 35.0 (ab) | 0.0, 84.2 | 4.2 (a) | 2.5, 7.5 | 6.7 (b) | 1.7, 11.7 | 240 |
| Zinc (%) | 7.1 | 2.9, 15.7 | 7.1 (ab) | 2.9, 17.1 | 7.1 (ab) | 0.0, 26.4 | 7.1 (a) | 1.4, 12.9 | 8.6 (b) | 4.3, 15.7 | 191 |
| Sodium (%) | 4.4 | 0.5, 15.8 | 1.1 (a) | 0.2, 4.8 | 2.0 (ab) | 0.2, 12.5 | 7.9 (b) | 0.6, 17.9 | 13.5 (c) | 3.3, 22.9 | 71 |
| Chloride (%) | 4.8 | 1.8, 15.6 | 2.8 (a) | 1.2, 5.1 | 0.0 (a) | 0.0, 1.3 | 8.0 (b) | 0.7, 16.8 | 13.6 (c) | 3.8, 24.0 | 87 |
| Iodine (%) | 0.7 | 2.1, 7.9 | 1.4 (a) | 0.7, 5.0 | 0.0 (a) | 0.0, 1.8 | 5.7 (b) | 1.4, 10.7 | 3.6 (b) | 0.7, 8.6 | 333 |
| Selenium (%) | 3.3 | 0.0, 11.7 | 1.7 (a) | 0.0, 11.7 | 0.0 (ab) | 0.0, 22.5 | 3.3 (ab) | 0.0, 12.1 | 5.0 (b) | 1.7, 11.7 | 464 |
|  |  |  |  |  |  |  |  |  |  |  |  |
| Overall (/18, Exc VitE, NA, CL) (n = 1845) | 10.3 | 6.3, 16.4 | 10.4 (a) | 6.7, 18.6 | 3.9 (ab) | 0.3, 39.7 | 8.6 (b) | 3.3, 14.3 | 10.5 (ab) | 6.3, 15.3 | 19 |
| Overall (/20, Inc NA, CL, Exc VItE) | 10.2 | 6.3, 16.2 | 9.8 (a) | 6.3, 17.2 | 4.1 (a) | 0.4, 36.1 | 9.4 (b) | 3.5, 14.2 | 11.2 (a) | 6.9, 16.0 | 16 |

*Overall (%) (/18) includes all micronutrients except Sodium, Chloride and Vitamin E. Overall (%) (/20) includes all micronutrients except Vitamin E. Unlike letters indicate significantly different P < 0.05. Pairwise comparisons conducted using Kruskal–Wallis ANOVA with Bonferroni correction for multiple comparisons. Items were classified into ‘healthy’ or ‘unhealthy’ based on the presence or absence of a red FOPL traffic light for fat, saturated fat, total sugar or salt. This is based on research that when identifying healthier products, UK consumers are more cautious to avoid items with red traffic lights, than to select items with green traffic light. Selenium: total = 1845 (UPF, n = 854). IQR: inter-quartile range; MPF: minimally processed food; PCI: processed culinary ingredients; PF: processed food; UPF: ultra-processed food.*

**Table 10. Average absolute micronutrient content of healthy items per 100g across each Nova group.**

| **Micronutrient** | **Total (n = 1846)** | | **MPF (n = 820)** | | **PCI (n = 9)** | | **PF (n = 162)** | | **UPF (n = 855)** | |
| --- | --- | --- | --- | --- | --- | --- | --- | --- | --- | --- |
|  | **Median** | **IQR** | **Median** | **IQR** | **Median** | **IQR** | **Median** | **IQR** | **Median** | **IQR** |
| Vitamin A (retinol equivalents) (µg) | 9.0 | 0.0, 52.0 | 12.0 (a) | 0.0, 58.0 | 0.0 (b) | 0.0, 0.0 | 14.0 (a) | 0.0, 0.0 | 6.0 (c) | 0.0, 44.0 |
| Vitamin D (µg) | 0.0 | 0.0, 0.2 | 0.0 (a) | 0.0, 0.1 | 0.0 (a) | 0.0, 0.0 | 0.0 (b) | 0.0, 0.4 | 0.0 (b) | 0.0, 0.2 |
| Thiamin (mg) | 0.08 | 0.03, 0.16 | 0.08 (a) | 0.04, 0.14 | 0.01 (ab) | 0.0, 0.36 | 0.04 (b) | 0.01, 0.09 | 0.1 (c) | 0.04, 0.21 |
| Riboflavin (mg) | 0.07 | 0.02, 0.15 | 0.06 (a) | 0.02, 0.15 | 0.06 (a) | 0.00, 0.88 | 0.07 (a) | 0.02, 0.14 | 0.08 (a) | 0.03, 0.15 |
| Niacin equivalent (mg) | 1.9 | 0.7, 4.7 | 1.3 (a) | 0.7, 4.7 | 0.0 (a) | 0, 6.5 | 1.5 (a) | 0.4, 3.63 | 2.6 (b) | 0.8, 4.8 |
| Vitamin C (mg) | 1.0 | 0.0, 8.0 | 2.9 (a) | 0, 15 | 0.0 (b) | 0.0, 0.0 | 1.1 (c) | 0, 6.5 | 0.1 (b) | 0.0, 3.9 |
| Vitamin E (mg) | 0.4 | 0.1, 1.1 | 0.3 (a) | 0.1, 0.9 | 0.0 (b) | 0.0, 0.0 | 0.4 (ac) | 0.1, 0.93 | 0.5 (c) | 0.1, 1.5 |
| Vitamin B6 (mg) | 0.1 | 0.1, 0.2 | 0.1 (a) | 0.1, 0.2 | 0.0 (ab) | 0, 0.3 | 0.1 (b) | 0, 0.2 | 0.1 (b) | 0.0, 0.2 |
| Vitamin B12 (µg) | 0.0 | 0.0, 0.5 | 0.0 (a) | 0, 0.6 | 0.0 (ac) | 0.0, 0.0 | 0.1 (b) | 0, 0.7 | 0.0 (bc) | 0.0, 0.4 |
| Folate (µg) B9 | 12.0 | 5.8, 26.0 | 12.0 (a) | 7, 28 | 1.0 (bc) | 0.0, 625.5 | 9.0 (b) | 4, 16 | 12.0 (ac) | 5.0, 27.0 |
|  |  |  |  |  |  |  |  |  |  |  |
| Potassium (mg) | 200.0 | 130.0, 290.3 | 229.0 (a) | 160, 335.8 | 7.0 (b) | 3.5, 322 | 163.5 (b) | 95, 231 | 182.0 (b) | 117.0, 252.0 |
| Calcium (mg) | 31.0 | 12.0, 85.0 | 22.0 (a) | 11, 44 | 7.0 (a) | 1.5, 18 | 30.0 (b) | 11, 79 | 50.0 (c) | 17.0, 110.0 |
| Magnesium (mg) | 18.0 | 10.0, 27.0 | 18.0 (a) | 10, 27 | 1.0 (b) | 0.0, 31.5 | 13.0 (b) | 8, 21 | 19.0 (a) | 11.0, 27.0 |
| Phosphorus (mg) | 88.0 | 30.0, 153.0 | 68.0 (a) | 28, 151 | 2.0 (a) | 0.0, 200.0 | 74.0 (a) | 16.8, 146.5 | 102.0 (b) | 47.0, 156.0 |
| Iron (mg) | 0.7 | 0.3, 1.5 | 0.7 (a) | 0.3, 1.6 | 2.7 (a) | 0.0, 3.9 | 0.6 (a) | 0.3, 1.1 | 0.8 (a) | 0.3, 1.6 |
| Copper (mg) | 0.07 | 0.03, 0.13 | 0.06 (a) | 0.03, 0.11 | 0.42 (ab) | 0.00, 1.01 | 0.05 (a) | 0.03, 0.09 | 0.08 (b) | 0.02, 0.14 |
| Zinc (mg) | 0.5 | 0.2, 1.1 | 0.5 (a) | 0.2, 1.2 | 0.5 (ab) | 0.0, 1.9 | 0.5 (a) | 0.1, 0.9 | 0.6 (b) | 0.3, 1.1 |
| Sodium | 70.0 | 8.0, 252.0 | 18.0 (a) | 3.0, 77.0 | 32.0 (ab) | 2.5, 200.0 | 126.0 (b) | 9.8, 287.0 | 216.0 (c) | 52.0, 366.0 |
| Chloride (mg) | 120.0 | 45.0, 389.0 | 70.0 (a) | 29.3, 128.3 | 0.0 (a) | 0.0, 33.5 | 201.0 (b) | 17.8, 420 | 339.0 (c) | 95.0, 601.0 |
| Iodine (µg) | 3.0 | 1.0, 11.0 | 2.0 (a) | 1.0, 7.0 | 0.0 (a) | 0.0, 2.5 | 8.0 (b) | 2.0, 15.0 | 5.0 (b) | 1.0, 12.0 |
| Selenium (µg) | 2.0 | 0.0, 7.0 | 1.0 (a) | 0.0, 7.0 | 0.0 (ab) | 0.0, 13.5 | 2.0 (ab) | 0.0, 7.3 | 3.0 (b) | 1.0, 7.0 |

*Unlike letters indicate significantly different P < 0.05. Pairwise comparisons conducted using Kruskal–Wallis ANOVA with Bonferroni correction for multiple comparisons. Items were classified into ‘healthy’ or ‘unhealthy’ based on the presence or absence of a red FOPL traffic light for fat, saturated fat, total sugar or salt. This is based on research that when identifying healthier products, UK consumers are more cautious to avoid items with red traffic lights, than to select items with green traffic lights. Selenium: total = 2953 (UPF, n = 1635). IQR: inter-quartile range; MPF: minimally processed food; PCI: processed culinary ingredients; PF: processed food; UPF: ultra-processed food.*

**Table 11. Average absolute micronutrient content of healthy items per 100kcal across each Nova group.**

| **Micronutrient** | **Total (n = 1825)** | | **MPF (n = 812)** | | **PCI (n = 9)** | | **PF (n = 162)** | | **UPF (n = 842)** | |
| --- | --- | --- | --- | --- | --- | --- | --- | --- | --- | --- |
|  | **Median** | **IQR** | **Median** | **IQR** | **Median** | **IQR** | **Median** | **IQR** | **Median** | **IQR** |
| Vitamin A (retinol equivalents) (µg) | 10.2 | 0.0, 54.5 | 17.6 (a) | 0.2, 109.3 | 0.0 (b) | 0.0, 0.0 | 15.5 (a) | 2.3, 49.7 | 5.1 (c) | 0.0, 34.1 |
| Vitamin D (µg) | 0.0 | 0.0, 0.1 | 0.0 (a) | 0.0, 0.1 | 0.0 (a) | 0.0, 0.0 | 0.0 (b) | 0.0, 0.2 | 0.0 (b) | 0.0, 0.1 |
| Thiamin (mg) | 0.08 | 0.04, 0.15 | 0.11 (a) | 0.06, 0.22 | 1.0 (abc) | 0.00, 1.17 | 0.04 (b) | 0.02, 0.08 | 0.08 (c) | 0.03, 0.12 |
| Riboflavin (mg) | 0.07 | 0.03, 0.14 | 0.09 (a) | 0.04, 0.19 | 2.4 (ab) | 0.0, 6.0 | 0.06 (b) | 0.03, 0.11 | 0.05 (b) | 0.02, 0.10 |
| Niacin equivalent (mg) | 1.9 | 1.04, 3.48 | 2.4 (a) | 1.33, 4.78 | 0.0 (b) | 0.0, 4.6 | 1.4 (b) | 0.7, 2.8 | 1.7 (b) | 0.9, 2.8 |
| Vitamin C (mg) | 0.9 | 0.0, 12.5 | 5.3 (a) | 0.0, 32.2 | 0.0 (b) | 0.0, 0.0 | 0.9 (c) | 0.0, 8.3 | 0.0 (b) | 0.0, 3.2 |
| Vitamin E (mg) | 0.4 | 0.1, 1.0 | 0.5 (a) | 0.1, 1.5 | 0.0 (b) | 0.0, 0.0 | 0.4 (c) | 0.1, 0.9 | 0.3 (c) | 0.1, 0.8 |
| Vitamin B6 (mg) | 0.1 | 0.03, 0.22 | 0.2 (a) | 0.1, 0.3 | 0.0 (b) | 0, 0.566 | 0.1 (b) | 0.0, 0.2 | 0.1 (b) | 0.0, 0.1 |
| Vitamin B12 (µg) | 0.0 | 0.0, 0.42 | 0.0 (a) | 0.0, 0.6 | 0.0 (a) | 0.0, 0.0 | 0.1 (b) | 0.0, 0.4 | 0.0 (ab) | 0.0, 0.4 |
| Folate (µg) B9 | 10.2 | 5.3, 27.3 | 16.5 (a) | 6.7, 60.5 | 100.0 (ab) | 0.0, 1229.2 | 6.8 (b) | 2.5, 17.3 | 8.8 (b) | 4.5, 16.0 |
|  |  |  |  |  |  |  |  |  |  |  |
| Potassium (mg) | 190.2 | 100.0, 376.8 | 348.8 (a) | 203.5, 603.8 | 700.0 (ab) | 3.3, 1000.5 | 165.6 (b) | 102.0, 240.3 | 114.7 (c) | 72.8, 189.9 |
| Calcium (mg) | 32.0 | 15.3, 77.6 | 30.8 (a) | 12.4, 103.4 | 47.3 (a) | 1.6, 700.0 | 28.5 (a) | 15.4, 66.7 | 33.1 (a) | 17.5, 67.4 |
| Magnesium (mg) | 17.2 | 10.7, 30.6 | 25.0 (a) | 15.6, 44.6 | 100.0 (ab) | 0.0, 105.7 | 12.6 (bc) | 9.1, 21.7 | 13.0 (c) | 8.9, 21.0 |
| Phosphorus (mg) | 77.9 | 47.8, 130.9 | 109.5 (a) | 58.6, 180.0 | 200.0 (ab) | 0.0, 492.9 | 63.7 (b) | 31.1, 97.0 | 66.0 (b) | 42.9, 90.3 |
| Iron (mg) | 0.7 | 0.4, 1.4 | 1.0 (a) | 0.5, 2.1 | 9.4 (ab) | 0.0, 270.0 | 0.5 (b) | 0.3, 1.1 | 0.5 (b) | 0.3, 0.9 |
| Copper (mg) | 0.06 | 0.03, 0.12 | 0.09 (a) | 0.05, 0.19 | 2.96 (ab) | 0.00, 42.00 | 0.05 (b) | 0.03, 0.09 | 0.05 (b) | 0.03, 0.08 |
| Zinc (mg) | 0.5 | 0.3, 1.0 | 0.8 (a) | 0.4, 1.4 | 4.73 (ab) | 0.0, 50.0 | 0.4 (b) | 0.2, 0.7 | 0.4 (b) | 0.2, 0.6 |
| Sodium | 82.6 | 18.7, 164.2 | 33.9 (a) | 5.8, 97.5 | 30.2 (abc) | 4.7, 20,000.0 | 93.8 (b) | 20.9, 175.2 | 137.0 (c) | 61.1, 208.0 |
| Chloride (mg) | 146.7 | 50.0, 281.9 | 100.0 (a) | 39.0, 211.9 | 0.0 (a) | 0.0, 205.3 | 147.8 (a) | 37.8, 261.7 | 207.2 (b) | 86.9, 323.1 |
| Iodine (µg) | 3.9 | 1.4, 9.6 | 4.8 (a) | 2.0, 11.1 | 0.0 (b) | 0.0, 2.1 | 7.6 (a) | 2.5, 12.0 | 3.1 (b) | 0.9, 7.3 |
| Selenium (µg) | 2.4 | 0.5, 4.8 | 2.9 (a) | 0.5, 6.8 | 0.0 (ab) | 0.0, 14.7 | 1.4 (b) | 0.0, 4.7 | 2.1 (b) | 0.8, 3.9 |

*Unlike letters indicate significantly different P < 0.05. Pairwise comparisons conducted using Kruskal–Wallis ANOVA with Bonferroni correction for multiple comparisons. Items were classified into ‘healthy’ or ‘unhealthy’ based on the presence or absence of a red FOPL traffic light for fat, saturated fat, total sugar or salt. This is based on research that when identifying healthier products, UK consumers are more cautious to avoid items with red traffic lights, than to select items with green traffic lights. Selenium: total = 1824 (UPF, n = 841). MPF: minimally processed food; PCI: processed culinary ingredients; PF: processed food; UPF: ultra-processed food.*

**Table 12. Average percentage contribution of unhealthy items per 100g to UK government dietary micronutrient recommendations from the UK NDNS for females aged 19-64 years, and across each Nova group.**

| **Micronutrient** | **Total (n = 1134)** | | **MPF (n = 166)** | | **PCI (n = 52)** | | **PF (n = 121)** | | **UPF (n = 795)** | | **Number of items with 0 micronutrient content per 100g** |
| --- | --- | --- | --- | --- | --- | --- | --- | --- | --- | --- | --- |
|  | **Median** | **IQR** | **Median** | **IQR** | **Median** | **IQR** | **Median** | **IQR** | **Median** | **IQR** |  |
| Vitamin A (retinol equivalents) (%) | 3.5 | 0.0, 18.3 | 3.0 (a) | 0.2, 19.7 | 0.0 (a) | 0.0, 53.2 | 20.3 (b) | 3.6, 42.6 | 2.7 (a) | 0.0, 13.2 | 381 |
| Vitamin D (%) | 0.0 | 0.0, 4.6 | 0.0 (a) | 0.0, 4.1 | 0.0 (a) | 0.0, 1.0 | 3.0 (b) | 0.4, 8.7 | 0.0 (a) | 0.0, 4.0 | 619 |
| Thiamin (%) | 11.3 | 3.8, 22.5 | 20.6 (a) | 10.0, 42.5 | 0.0 (b) | 0.0, 0.9 | 10.0 (c) | 3.8, 16.3 | 10.0 (c) | 3.8, 21.3 | 182 |
| Riboflavin (%) | 11.8 | 4.5, 20.0 | 16.4 (a) | 8.2, 26.4 | 0.0 (b) | 0.0, 15.2 | 18.2 (a) | 8.2, 28.2 | 10.9 (c) | 3.6, 17.3 | 130 |
| Niacin equivalent (%) | 17.7 | 6.9, 42.3 | 30.0 (a) | 9.8, 71.0 | 0.0 (b) | 0.0, 2.9 | 33.1 (a) | 16.9, 53.1 | 16.2 (c) | 6.9, 34.6 | 111 |
| Vitamin C (%) | 0.0 | 0.0, 2.0 | 0.0 (a) | 0.0, 7.8 | 0.0 (b) | 0.0, 0.0 | 0.0 (b) | 0.0, 0.9 | 0.0 (b) | 0.0, 2.0 | 752 |
| Vitamin E (%) | 30.0 | 6.7, 83.3 | 45.0 (a) | 10.0, 110.8 | 25.0 (a) | 0.0, 187.5 | 20.0 (a) | 10.0, 66.7 | 30.0 (a) | 6.7, 73.3 | 157 |
| Vitamin B6 (%) | 8.3 | 0.0, 16.7 | 25.0 (a) | 8.3, 33.3 | 0.0 (b) | 0.0, 0.0 | 8.3 (c) | 8.3, 16.7 | 8.3 (c) | 0.0, 16.7 | 371 |
| Vitamin B12 (%) | 6.7 | 0.0, 46.7 | 0.0 (a) | 0.0, 80.0 | 0.0 (b) | 0.0, 13.3 | 40.0 (c) | 6.7, 113.3 | 6.7 (a) | 0.0, 33.3 | 526 |
| Folate (%) | 4.5 | 1.5, 10.0 | 6.3 (a) | 2.0, 25.1 | 0.0 (b) | 0.0, 0.0 | 7.5 (a) | 4.0, 15.5 | 4.0 (c) | 1.5, 8.0 | 176 |
|  |  |  |  |  |  |  |  |  |  |  |  |
| Potassium (%) | 5.6 | 3.1, 9.7 | 11.9 (a) | 7.5, 23.5 | 0.8 (b) | 0.0, 2.9 | 4.3 (c) | 2.8, 8.3 | 5.4 (c) | 3.2, 8.7 | 24 |
| Calcium (%) | 8.6 | 2.6, 17.1 | 8.3 (a) | 2.4, 21.1 | 0.7 (b) | 0.0, 9.6 | 12.1 (c) | 5.7, 44.3 | 8.4 (a) | 2.4, 15.7 | 22 |
| Magnesium (%) | 7.8 | 4.1, 15.2 | 11.1 (a) | 6.3, 49.4 | 0.6 (b) | 0.0, 3.2 | 7.4 (c) | 5.6, 12.2 | 7.8 (c) | 4.1, 14.1 | 43 |
| Phosphorus (%) | 23.6 | 11.1, 38.4 | 34.0 (a) | 16.1, 54.5 | 0.5 (b) | 0.0, 10.3 | 34.7 (a) | 20.5, 60.0 | 22.4 (c) | 10.7, 36.0 | 37 |
| Iron (%) | 7.4 | 2.7, 13.5 | 12.8 (a) | 6.1, 26.4 | 0.7 (b) | 0.0, 2.5 | 5.4 (c) | 2.0, 9.5 | 7.4 (d) | 3.4, 12.2 | 83 |
| Copper (%) | 9.2 | 4.2, 20.8 | 13.3 (a) | 5.0, 50.4 | 0.8 (b) | 0.0, 3.5 | 7.5 (c) | 4.2, 15.8 | 10.0 (c) | 5.0, 19.2 | 155 |
| Zinc (%) | 10.0 | 4.3, 20.0 | 22.9 (a) | 5.7, 48.9 | 1.4 (b) | 0.0, 3.9 | 15.7 (a) | 9.3, 41.4 | 10.0 (c) | 4.3, 17.1 | 100 |
| Sodium (%) | 13.7 | 3.9, 34.0 | 2.8 (a) | 0.9, 9.3 | 0.7 (a) | 0.0, 2.5 | 24.2 (b) | 12.0, 43.1 | 16.3 (b) | 5.7, 36.3 | 28 |
| Chloride (%) | 11.7 | 3.6, 33.6 | 3.2 (a) | 1.2, 10.6 | 0.7 (b) | 0.0, 3.2 | 22.3 (c) | 10.7, 41.6 | 13.4 (d) | 5.0, 35.1 | 42 |
| Iodine (%) | 5.7 | 1.4, 12.9 | 5.4 (a) | 1.4, 14.3 | 0.0 (b) | 0.0, 20.7 | 9.3 (c) | 5.7, 21.4 | 5.7 (a) | 2.1, 11.4 | 180 |
| Selenium (%) | 5.0 | 1.7, 10.0 | 6.7 (a) | 1.7, 21.7 | 0.0 (b) | 0.0, 0.0 | 8.3 (a) | 5.0, 12.5 | 5.0 (c) | 1.7, 10.0 | 229 |
|  |  |  |  |  |  |  |  |  |  |  |  |
| Overall (%) (/18, Exc VitE, NA, CL) (n = 1133) | 13.1 | 8.0, 22.7 | 25.7 (a) | 13.0, 45.1 | 3.0 (b) | 0.1, 9.8 | 18.8 (a) | 11.1, 30.7 | 12.0 (c) | 7.5, 17.8 | 6 |
| Overall (%) (/20, Inc NA, CL, Exc VItE) (n = 1133) | 14.1 | 8.6, 24.6 | 24.4 (a) | 12.7, 42.1 | 5.8 (b) | 0.1, 11.9 | 19.7 (a) | 11.7, 30.5 | 12.6 (c) | 8.2, 21.0 | 4 |

*Overall (%) (/18) includes all micronutrients except Sodium, Chloride and Vitamin E. Overall (%) (/20) includes all micronutrients except Vitamin E. Unlike letters indicate significantly different P < 0.05. Pairwise comparisons conducted using Kruskal–Wallis ANOVA with Bonferroni correction for multiple comparisons. Items were classified into ‘healthy’ or ‘unhealthy’ based on the presence or absence of a red FOPL traffic light for fat, saturated fat, total sugar or salt. This is based on research that when identifying healthier products, UK consumers are more cautious to avoid items with red traffic lights, than to select items with green traffic lights. Selenium: total = 1133 (UPF, n = 794). IQR: inter-quartile range; MPF: minimally processed food; PCI: processed culinary ingredients; PF: processed food; UPF: ultra-processed food.*

**Table 13. Average percentage contribution of unhealthy items per 100kcal to UK government dietary micronutrient recommendations from the UK NDNS for females aged 19-64 years, and across each Nova group.**

| **Micronutrient** | **Total (n = 1130)** | | **MPF (n = 166)** | | **PCI (n = 48)** | | **PF (n = 121)** | | **UPF (n = 795)** | | **Number of items with 0 content of micronutrient per 100kcal** |
| --- | --- | --- | --- | --- | --- | --- | --- | --- | --- | --- | --- |
|  | **Median** | **IQR** | **Median** | **IQR** | **Median** | **IQR** | **Median** | **IQR** | **Median** | **IQR** |  |
| Vitamin A (retinol equivalents) (%) | 1.1 | 0.0, 7.0 | 1.0 (a) | 0.0, 10.2 | 0.0 (ac) | 0.0, 23.1 | 7.2 (b) | 1.2, 14.8 | 0.9 (c) | 0.0, 4.5 | 377 |
| Vitamin D (%) | 0.0 | 0.0, 1.4 | 0.0 (a) | 0.0, 2.0 | 0.0 (a) | 0.0, 0.5 | 0.8 (b) | 0.1, 2.8 | 0.0 (a) | 0.0, 1.4 | 615 |
| Thiamin (%) | 3.6 | 1.3, 7.9 | 7.4 (a) | 4.9, 14.7 | 0.0 (b) | 0.0, 0.5 | 3.4 (c) | 1.2, 5.2 | 3.2 (c) | 1.3, 7.0 | 178 |
| Riboflavin (%) | 3.8 | 1.4, 8.6 | 6.5 (a) | 2.9, 11.3 | 0.0 (b) | 0.0, 3.9 | 6.8 (a) | 2.4, 10.2 | 3.3 (c) | 1.1, 7.3 | 126 |
| Niacin equivalent (%) | 5.8 | 2.6, 13.7 | 12.0 (a) | 6.1, 24.4 | 0.0 (b) | 0.0, 0.9 | 11.6 (a) | 6.0, 16.9 | 4.4 (c) | 2.2, 11.6 | 107 |
| Vitamin C (%) | 0.0 | 0.0, 0.7 | 0.0 (a) | 0.0, 7.6 | 0.0 (b) | 0.0, 0.0 | 0.0 (b) | 0.0, 0.2 | 0.0 (b) | 0.0, 0.6 | 748 |
| Vitamin E (%) | 10.0 | 3.5, 24.2 | 16.0 (a) | 5.1, 42.5 | 8.5 (b) | 0.0, 42.2 | 6.9 (b) | 4.1, 21.8 | 10 (b) | 2.8, 20.6 | 157 |
| Vitamin B6 (%) | 2.6 | 0.0, 7.7 | 8.7 (a) | 5.9, 12.1 | 0.0 (b) | 0.0, 0.0 | 3.1 (c) | 2.0, 6.3 | 2.1 (d) | 0.0, 5.8 | 367 |
| Vitamin B12 (%) | 1.7 | 0.0, 17.0 | 0.0 (a) | 0.0, 47.8 | 0.0 (b) | 0.0, 2.7 | 12.5 (c) | 1.9, 38.5 | 1.7 (a) | 0.0, 12.9 | 522 |
| Folate (%) | 1.4 | 0.5, 3.9 | 3.6 (a) | 0.9, 7.7 | 0.0 (b) | 0.0, 0.3 | 3.4 (a) | 1.2, 5.3 | 1.2 (c) | 0.5, 2.9 | 172 |
|  |  |  |  |  |  |  |  |  |  |  |  |
| Potassium (%) | 1.9 | 1.0, 3.5 | 4.6 (a) | 3.2, 9.4 | 0.1 (b) | 0.0, 1.2 | 1.6 (c) | 0.9, 3.4 | 1.7 (c) | 1.0, 2.9 | 23 |
| Calcium (%) | 2.8 | 1.0, 6.1 | 3.1 (a) | 1.0, 8.2 | 0.2 (b) | 0.0, 3.2 | 4.7 (c) | 1.9, 20.6 | 2.7 (d) | 1.0, 5.1 | 20 |
| Magnesium (%) | 2.9 | 1.5, 4.9 | 5.7 (a) | 3.1, 13.9 | 0.1 (b) | 0.0, 1.5 | 3.0 (c) | 1.9, 4.5 | 2.7 (c) | 1.4, 4.3 | 41 |
| Phosphorus (%) | 7.5 | 4.1, 13.6 | 13.2 (a) | 7.3, 19.2 | 0.2 (a) | 0.0, 2.8 | 12.9 (a) | 6.4, 21.0 | 6.6 (c) | 3.8, 11.7 | 33 |
| Iron (%) | 2.3 | 1.1, 4.2 | 4.7 (a) | 2.3, 11.8 | 0.1 (b) | 0.0, 0.4 | 1.8 (c) | 0.6, 3.2 | 2.2 (d) | 1.3, 3.7 | 81 |
| Copper (%) | 3.1 | 1.4, 6.2 | 7.6 (a) | 2.3, 15.0 | 0.1 (b) | 0.0, 0.4 | 2.6 (c) | 1.3, 7.7 | 3.1 (c) | 1.6, 5.6 | 153 |
| Zinc (%) | 3.2 | 1.7, 8.3 | 8.7 (a) | 3.8, 14.5 | 0.2 (b) | 0.0, 2.1 | 6.4 (a) | 2.9, 13.8 | 2.7 (c) | 1.5, 6.1 | 96 |
| Sodium (%) | 3.9 | 1.3, 10.6 | 1.4 (a) | 0.2, 4.1 | 0.1 (b) | 0.0, 0.9 | 7.2 (c) | 3.5, 12.8 | 4.5 (d) | 1.9, 11.3 | 28 |
| Chloride (%) | 3.7 | 1.2, 9.8 | 1.3 (a) | 0.4, 5.0 | 0.1 (b) | 0.0, 1.4 | 6.7 (c) | 3.5, 12.8 | 3.9 (d) | 1.5, 11.0 | 41 |
| Iodine (%) | 2.0 | 0.6, 4.5 | 2.0 (a) | 0.8, 6.2 | 0.0 (b) | 0.0, 3.0 | 3.3 (c) | 1.7, 7.4 | 1.9 (a) | 0.5, 4.2 | 178 |
| Selenium (%) | 1.5 | 0.5, 3.4 | 2.8 (a) | 0.6, 7.6 | 0.0 (a) | 0.0, 0.0 | 2.4 (a) | 1.2, 4.4 | 1.3 (c) | 0.5, 3.2 | 225 |
|  |  |  |  |  |  |  |  |  |  |  |  |
| Overall (%) (/18, Exc VitE, NA, CL) (n = 1129) | 4.4 | 2.2, 8.6 | 9.8 (a) | 6.0, 15.2 | 0.6 (b) | 0.0, 2.9 | 6.8 (c) | 3.5, 9.3 | 3.5 (d) | 2.0, 7.0 | 5 |
| Overall (%) (/20, Inc NA, CL, Exc VItE) (n = 1129) | 4.7 | 2.3, 9.4 | 9.1 (a) | 5.7, 14.5 | 0.6 (b) | 0.0, 2.6 | 7.3 (c) | 4.2, 10.5 | 3.7 (d) | 2.2, 8.4 | 5 |

*Overall (%) (/18) includes all micronutrients except Sodium, Chloride and Vitamin E. Overall (%) (/20) includes all micronutrients except Vitamin E. Unlike letters indicate significantly different P < 0.05. Pairwise comparisons conducted using Kruskal–Wallis ANOVA with Bonferroni correction for multiple comparisons. Items were classified into ‘healthy’ or ‘unhealthy’ based on the presence or absence of a red FOPL traffic light for fat, saturated fat, total sugar or salt. This is based on research that when identifying healthier products, UK consumers are more cautious to avoid items with red traffic lights, than to select items with green traffic lights. Selenium: total = 1129 (UPF, n = 794). IQR: inter-quartile range; MPF: minimally processed food; PCI: processed culinary ingredients; PF: processed food; UPF: ultra-processed food.*

**Table 14. Statistical comparison between average percentage contribution to UK government dietary micronutrient recommendations from the UK NDNS for females aged 19-64 years, of healthy and unhealthy items across each Nova group, per 100g and per 100kcal.**

| **Micronutrient** | **Healthy vs Unhealthy comparison % contribution per 100g** | | | | |  | **Healthy vs Unhealthy comparison % contribution per 100kcal** | | | | |
| --- | --- | --- | --- | --- | --- | --- | --- | --- | --- | --- | --- |
|  | **Healthy vs Unhealthy Overall (n = 2980)** | **Healthy vs Unhealthy MPF (n = 986)** | **Healthy vs Unhealthy PCI (n = 61)** | **Healthy vs Unhealthy PF (n = 283)** | **Healthy vs Unhealthy UPF (n = 1650)** |  | **Healthy vs Unhealthy Overall (n = 2955)** | **Healthy vs Unhealthy MPF (n = 978)** | **Healthy vs Unhealthy PCI (n = 57)** | **Healthy vs Unhealthy PF (n = 283)** | **Healthy vs Unhealthy UPF (n = 1637)** |
|  | **p-value** | **p-value** | **p-value** | **p-value** | **p-value** |  | **p-value** | **p-value** | **p-value** | **p-value** | **p-value** |
| Vitamin A (retinol equivalents) (%) | <0.001 | 0.113 | 0.041 | <0.001 | <0.001 |  | <0.001 | 0.008 | 0.032 | <0.001 | 0.657 |
| Vitamin D (%) | <0.001 | 0.003 | 0.069 | <0.001 | 0.164 |  | <0.001 | 0.021 | 0.056 | 0.005 | 0.549 |
| Thiamin (%) | 0.952 | <0.001 | 0.093 | <0.001 | 0.005 |  | <0.001 | <0.001 | 0.013 | 0.002 | <0.001 |
| Riboflavin (%) | <0.001 | <0.001 | 0.434 | <0.001 | <0.001 |  | <0.001 | 0.005 | 0.072 | 0.557 | <0.001 |
| Niacin equivalent (%) | <0.001 | <0.001 | 0.564 | <0.001 | 0.203 |  | <0.001 | <0.001 | 0.480 | 0.686 | <0.001 |
| Vitamin C (%) | <0.001 | <0.001 | 0.154 | <0.001 | <0.001 |  | <0.001 | <0.001 | 0.137 | <0.001 | <0.001 |
| Vitamin E (%) | <0.001 | <0.001 | <0.001 | <0.001 | <0.001 |  | 0.001 | 0.896 | <0.001 | 0.277 | 0.241 |
| Vitamin B6 (%) | 0.639 | <0.001 | 0.076 | 0.015 | 0.282 |  | <0.001 | <0.001 | 0.094 | <0.001 | <0.001 |
| Vitamin B12 (%) | <0.001 | 0.020 | 0.049 | <0.001 | 0.023 |  | 0.128 | 0.223 | 0.038 | 0.020 | 0.179 |
| Folate (%) | <0.001 | 0.863 | 0.072 | <0.001 | <0.001 |  | <0.001 | <0.001 | 0.009 | 0.050 | <0.001 |
|  |  |  |  |  |  |  |  |  |  |  |  |
| Potassium (%) | 0.439 | <0.001 | 0.885 | 0.429 | 0.002 |  | <0.001 | <0.001 | 0.010 | <0.001 | <0.001 |
| Calcium (%) | <0.001 | <0.001 | 0.812 | <0.001 | 0.043 |  | <0.001 | 0.002 | 0.021 | 0.300 | <0.001 |
| Magnesium (%) | <0.001 | <0.001 | 0.884 | <0.001 | 0.005 |  | <0.001 | <0.001 | 0.029 | <0.001 | <0.001 |
| Phosphorus (%) | <0.001 | <0.001 | 0.932 | <0.001 | <0.001 |  | <0.001 | <0.001 | 0.033 | 0.233 | <0.001 |
| Iron (%) | <0.001 | <0.001 | 0.217 | 0.260 | <0.001 |  | <0.001 | 0.006 | 0.176 | <0.001 | <0.001 |
| Copper (%) | <0.001 | <0.001 | 0.056 | <0.001 | <0.001 |  | <0.001 | 0.045 | 0.039 | 0.038 | <0.001 |
| Zinc (%) | <0.001 | <0.001 | 0.082 | <0.001 | 0.009 |  | <0.001 | <0.001 | 0.039 | 0.055 | <0.001 |
| Sodium (%) | <0.001 | <0.001 | 0.364 | <0.001 | <0.001 |  | 0.517 | 0.010 | 0.017 | 0.012 | <0.001 |
| Chloride (%) | <0.001 | 0.003 | 0.150 | <0.001 | <0.001 |  | <0.001 | <0.001 | 0.552 | 0.033 | <0.001 |
| Iodine (%) | <0.001 | <0.001 | 0.427 | <0.001 | <0.001 |  | <0.001 | 0.001 | 0.859 | 0.167 | 0.155 |
| Selenium (%) | <0.014 | <0.001 | 0.013 | <0.001 | 0.248 |  | <0.001 | 0.021 | 0.013 | 0.340 | <0.001 |
|  |  |  |  |  |  |  |  |  |  |  |  |
| Overall female (%) (/18, Exc VitE, NA, CL) | <0.001 | <0.001 | 0.027 | 0.012 | <0.001 |  | <0.001 | <0.001 | 1.000 | <0.001 | <0.001 |
| Overall male (%) (/18, Exc VitE, NA, CL) | <0.001 | <0.001 | 0.032 | 0.006 | <0.001 |  | <0.001 | <0.001 | 0.968 | <0.001 | <0.001 |

*Overall (%) (/18) includes all micronutrients except Sodium, Chloride and Vitamin E. Overall (%) (/20) includes all micronutrients except Vitamin E. Unlike letters indicate significantly different P < 0.05. Pairwise comparisons conducted using Kruskal–Wallis ANOVA with Bonferroni correction for multiple comparisons. Items were classified into ‘healthy’ or ‘unhealthy’ based on the presence or absence of a red FOPL traffic light for fat, saturated fat, total sugar or salt. This is based on research that when identifying healthier products, UK consumers are more cautious to avoid items with red traffic lights, than to select items with green traffic lights. Selenium: total (g) = 2978 (UPF, n = 1648); Selenium: total (kcal) = 2953 (UPF, n = 1635). IQR: inter-quartile range; MPF: minimally processed food; PCI: processed culinary ingredients; PF: processed food; UPF: ultra-processed food.*

**Table 15. Average percentage contribution of healthy items per 100g to UK government dietary micronutrient recommendations from the UK NDNS for males aged 19-64 years, and across each Nova group.**

| **Micronutrient** | **Total (n = 1846)** | | **MPF (n = 820)** | | **PCI (n = 9)** | | **PF (n = 162)** | | **UPF (n = 855)** | |
| --- | --- | --- | --- | --- | --- | --- | --- | --- | --- | --- |
|  | **Median** | **IQR** | **Median** | **IQR** | **Median** | **IQR** | **Median** | **IQR** | **Median** | **IQR** |
| Vitamin A (retinol equivalents) (%) | 1.3 | 0.0, 7.4 | 1.7 (a) | 0.0, 8.3 | 0.0 (b) | 0.0, 0.0 | 2.0 (a) | 0.3, 9.1 | 0.9 (c) | 0.0, 6.3 |
| Vitamin D (%) | 0.0 | 0.0, 1.6 | 0.0 (a) | 0.0, 0.7 | 0.0 (a) | 0.0, 0.0 | 0.2 (b) | 0.0, 4.0 | 0.0 (b) | 0.0, 1.7 |
| Thiamin (%) | 8.0 | 3.0, 16.0 | 8.0 (a) | 4.0, 13.8 | 1.0 (ab) | 0.0, 36.0 | 4.0 (b) | 1.0, 9.0 | 10.0 (c) | 4.0, 21.0 |
| Riboflavin (%) | 5.4 | 1.5, 11.5 | 4.6 (a) | 1.5, 11.5 | 4.6 (a) | 0.0, 67.7 | 5.0 (a) | 1.5, 10.8 | 6.2 (a) | 2.3, 11.5 |
| Niacin equivalent (%) | 11.2 | 4.1, 27.6 | 7.6 (a) | 4.1, 27.4 | 0.0 (a) | 0.0, 38.2 | 8.8 (a) | 2.4, 21.3 | 15.3 (b) | 4.7, 28.2 |
| Vitamin C (%) | 2.5 | 0.0, 20.0 | 7.3 (a) | 0.0, 37.5 | 0.0 (b) | 0.0, 0.0 | 2.6 (c) | 0.0, 16.3 | 0.3 (b) | 0.0, 9.8 |
| Vitamin E (%) | 10.0 | 2.5, 27.5 | 7.5 (a) | 2.5, 22.5 | 0.0 (b) | 0.0, 0.0 | 10.0 (ac) | 2.5, 23.1 | 12.5 (c) | 2.5, 37.5 |
| Vitamin B6 (%) | 7.1 | 7.1, 14.3 | 7.1 (a) | 7.1, 14.3 | 0.0 (ab) | 0.0, 21.4 | 7.1 (b) | 0.0, 14.3 | 7.1 (b) | 0.0, 14.3 |
| Vitamin B12 (%) | 0.0 | 0.0, 33.3 | 0.0 (a) | 0.0, 40.0 | 0.0 (ac) | 0.0, 0.0 | 6.7 (b) | 0.0, 46.7 | 0.0 (bc) | 0.0, 26.7 |
| Folate (%) | 6.0 | 2.9, 13.0 | 6.0 (a) | 3.5, 14.0 | 0.5 (bc) | 0.0, 312.8 | 4.5 (b) | 2.0, 8.0 | 6.0 (ac) | 2.5, 13.5 |
|  |  |  |  |  |  |  |  |  |  |  |
| Potassium (%) | 5.7 | 3.7, 8.3 | 6.5 (a) | 4.6, 9.6 | 0.2 (b) | 0.1, 9.2 | 4.7 (b) | 2.7, 6.6 | 5.2 (b) | 3.3, 7.2 |
| Calcium (%) | 4.4 | 1.7, 12.1 | 3.1 (a) | 1.6, 6.3 | 1.0 (a) | 0.2, 2.6 | 4.3 (b) | 1.6, 11.3 | 7.1 (c) | 2.4, 15.7 |
| Magnesium (%) | 6.0 | 3.3, 9.0 | 6.0 (a) | 3.3, 9.0 | 0.3 (b) | 0.0, 10.5 | 4.3 (b) | 2.7, 7.0 | 6.3 (a) | 3.7, 9.0 |
| Phosphorus (%) | 16 | 5.5, 27.8 | 12.4 (a) | 5.1, 27.5 | 0.4 (a) | 0.0, 36.4 | 13.5 (a) | 3.0, 26.6 | 18.5 (b) | 8.5, 28.4 |
| Iron (%) | 8.0 | 3.4, 17.2 | 8.0 (a) | 3.4, 18.4 | 31.0 (a) | 0.0, 44.3 | 6.9 (a) | 3.4, 12.9 | 9.2 (a) | 3.4, 18.4 |
| Copper (%) | 5.8 | 2.5, 10.8 | 5.0 (a) | 2.5, 9.2 | 35.0 (ab) | 0.0, 84.2 | 4.2 (a) | 2.5, 7.5 | 6.7 (b) | 1.7, 11.7 |
| Zinc (%) | 5.3 | 2.1, 11.6 | 5.3 (a) | 2.1, 12.6 | 5.3 (ab) | 0.0, 19.5 | 5.3 (a) | 1.1, 9.5 | 6.3 (b) | 3.2, 11.6 |
| Sodium (%) | 4.4 | 0.5, 15.8 | 1.1 (a) | 0.2, 4.8 | 2.0 (ab) | 0.2, 12.5 | 7.9 (b) | 0.6, 17.9 | 13.5 (c) | 3.3, 22.9 |
| Chloride (%) | 4.8 | 1.8, 15.6 | 2.8 (a) | 1.2, 5.1 | 0.0 (a) | 0, 1.3 | 8.0 (b) | 0.7, 16.8 | 13.6 (c) | 3.8, 24.0 |
| Iodine (%) | 0.7 | 2.1, 7.9 | 1.4 (a) | 0.7, 5.0 | 0.0 (a) | 0.0, 1.8 | 5.7 (b) | 1.4, 10.7 | 3.6 (b) | 0.7, 8.6 |
| Selenium (%) | 2.7 | 0.0, 9.3 | 1.3 (a) | 0.0, 9.3 | 0.0 (ab) | 0.0, 18.0 | 2.7 (ab) | 0.0, 9.7 | 4.0 (b) | 1.3, 9.3 |
|  |  |  |  |  |  |  |  |  |  |  |
| Overall (%) (/18, Exc VitE, NA, CL) (n = 1845) | 9.7 | 5.9, 15.1 | 9.8 (a) | 6.4, 17.1 | 4.4 (abc) | 0.3, 38.1 | 8.2 (bc) | 3.7, 13.3 | 9.8 (c) | 6.0, 14.1 |
| Overall (%) (/20, Inc NA, CL, Exc VItE) (n = 1845) | 9.8 | 6.0, 15.1 | 9.2 (a) | 6.0, 15.7 | 4.6 (abc) | 0.3, 34.7 | 8.8 (b) | 3.4, 13.7 | 10.6 (c) | 6.5, 14.9 |

*Overall (%) (/18) includes all micronutrients except Sodium, Chloride and Vitamin E. Overall (%) (/20) includes all micronutrients except Vitamin E. Unlike letters indicate significantly different P < 0.05. Pairwise comparisons conducted using Kruskal–Wallis ANOVA with Bonferroni correction for multiple comparisons. Items were classified into ‘healthy’ or ‘unhealthy’ based on the presence or absence of a red FOPL traffic light for fat, saturated fat, total sugar or salt. This is based on research that when identifying healthier products, UK consumers are more cautious to avoid items with red traffic lights, than to select items with green traffic lights. Selenium: total = 1845 (UPF, n = 854). IQR: inter-quartile range; MPF: minimally processed food; PCI: processed culinary ingredients; PF: processed food; UPF: ultra-processed food.*

**Table 16. Average percentage contribution of healthy items per 100kcal to UK government dietary micronutrient recommendations from the UK NDNS for males aged 19-64 years, and across each Nova group.**

| **Micronutrient** | **Total (n = 1825)** | | **MPF (n = 812)** | | **PCI (n = 9)** | | **PF (n = 162)** | | **UPF (n = 842)** | |
| --- | --- | --- | --- | --- | --- | --- | --- | --- | --- | --- |
|  | **Median** | **IQR** | **Median** | **IQR** | **Median** | **IQR** | **Median** | **IQR** | **Median** | **IQR** |
| Vitamin A (retinol equivalents) (%) | 1.5 | 0.0, 7.8 | 2.5 (a) | 0.0, 15.6 | 0.0 (b) | 0.0, 0.0 | 2.2 (a) | 0.3, 7.1 | 0.7 (c) | 0.0, 4.9 |
| Vitamin D (%) | 0.0 | 0.0, 1.1 | 0.0 (a) | 0.0, 0.8 | 0.0 (a) | 0.0, 0.0 | 0.1 (b) | 0.0, 2.2 | 0.0 (b) | 0.0, 1.1 |
| Thiamin (%) | 8.2 | 4.1, 15.4 | 10.8 (a) | 5.9, 21.9 | 100.0 (abc) | 0.0, 117.0 | 4.1 (b) | 1.9, 7.6 | 7.5 (c) | 3.4, 12.4 |
| Riboflavin (%) | 5.1 | 2.3, 10.5 | 7.0 (a) | 3.4, 14.9 | 182.1 (ab) | 0.0, 461.5 | 4.6 (b) | 2.3, 8.5 | 3.8 (b) | 1.6, 7.7 |
| Niacin equivalent (%) | 11.3 | 6.1, 20.5 | 14.0 (a) | 7.8, 28.1 | 0.0 (b) | 0.0, 27.0 | 8.3 (b) | 4.2, 16.5 | 10.1 (b) | 5.1, 16.2 |
| Vitamin C (%) | 2.2 | 0.0, 31.1 | 13.3 (a) | 0.0, 80.6 | 0.0 (b) | 0.0, 0.0 | 2.2 (c) | 0.0, 20.9 | 0.1 (b) | 0.0, 8.1 |
| Vitamin E (%) | 9.3 | 2.4, 25.5 | 13.2 (a) | 3.4, 37.8 | 0.0 (b) | 0.0, 0.0 | 9.7 (c) | 2.9, 22.2 | 8.2 (c) | 2.1, 20.8 |
| Vitamin B6 (%) | 6.6 | 2.0, 15.7 | 12.0 (a) | 5.4, 23.8 | 0.0 (b) | 0.0, 40.4 | 5.9 (b) | 0.0, 11.5 | 4.1 (b) | 0.0, 8.9 |
| Vitamin B12 (%) | 0.0 | 0.0, 27.9 | 0.0 (a) | 0.0, 36.7 | 0.0 (a) | 0.0, 0.0 | 5.5 (b) | 0.0, 29.4 | 0.0 (ab) | 0.0, 24.5 |
| Folate (%) | 5.1 | 2.6, 13.7 | 8.2 (a) | 3.3, 30.2 | 50.0 (ab) | 0.0, 614.6 | 3.4 (b) | 1.8, 8.6 | 4.4 (b) | 2.3, 8.0 |
|  |  |  |  |  |  |  |  |  |  |  |
| Potassium (%) | 5.4 | 2.9, 10.8 | 10.0 (a) | 5.8, 17.3 | 20.0 (ab) | 0.1, 28.6 | 4.7 (b) | 2.9, 6.9 | 3.3 (c) | 2.1, 5.4 |
| Calcium (%) | 4.6 | 2.2, 11.1 | 4.4 (a) | 1.8, 14.8 | 6.8 (a) | 0.2, 100.0 | 4.1 (a) | 2.2, 9.5 | 4.7 (a) | 2.5, 9.6 |
| Magnesium (%) | 5.7 | 3.6, 10.2 | 8.3 (a) | 5.2, 14.9 | 33.3 (ab) | 0.0, 35.2 | 4.2 (bc) | 3.0, 7.2 | 4.3 (c) | 3.0, 7.0 |
| Phosphorus (%) | 14.2 | 8.7, 23.8 | 19.9 (a) | 10.7, 32.7 | 36.4 (ab) | 0.0, 89.6 | 11.6 (b) | 5.7, 17.6 | 12.0 (b) | 7.8, 16.4 |
| Iron (%) | 7.8 | 4.2, 15.5 | 11.8 (a) | 5.1, 24.6 | 108.4 (ab) | 0.0, 3103.4 | 6.0 (b) | 3.9, 12.3 | 6.3 (b) | 3.7, 10.0 |
| Copper (%) | 5.4 | 2.8, 9.7 | 7.4 (a) | 3.8, 15.8 | 246.5 (ab) | 0.0, 3500.0 | 4.3 (b) | 2.2, 7.6 | 4.4 (b) | 2.4, 6.9 |
| Zinc (%) | 5.4 | 2.8, 10.2 | 8.4 (a) | 3.9, 15.0 | 49.8 (ab) | 0.0, 526.3 | 4.3 (b) | 1.8, 7.0 | 4.1 (b) | 2.4, 6.7 |
| Sodium (%) | 5.2 | 1.2, 10.3 | 2.1 (a) | 0.4, 6.1 | 1.9 (abc) | 0.3, 1250.0 | 5.9 (b) | 1.3, 11.0 | 8.6 (c) | 3.8, 13.0 |
| Chloride (%) | 5.9 | 2.0, 11.3 | 4.0 (a) | 1.6, 8.5 | 0.0 (a) | 0.0, 8.2 | 5.9 (a) | 1.5, 10.5 | 8.3 (b) | 3.5, 12.9 |
| Iodine (%) | 2.8 | 1.0, 6.8 | 3.4 (a) | 1.4, 7.9 | 0.0 (b) | 0.0, 1.5 | 5.4 (a) | 1.8, 8.6 | 2.2 (b) | 0.6, 5.2 |
| Selenium (%) | 3.1 | 0.6, 6.3 | 3.9 (a) | 0.6, 9.0 | 0.0 (ab) | 0.0, 19.5 | 1.9 (b) | 0.0, 6.3 | 2.8 (b) | 1.0, 5.2 |
|  |  |  |  |  |  |  |  |  |  |  |
| Overall (%) (/18, Exc VitE, NA, CL) (n = 1824) | 9.4 | 5.7, 17.8 | 14.6 (a) | 8.5, 29.8 | 128.9 (ab) | 0.3, 440.6 | 7.4 (b) | 5.3, 11.8 | 6.7 (b) | 4.7, 10.6 |
| Overall (%) (/20, Inc NA, CL, Exc VItE) (n = 1824) | 9.3 | 6.0, 16.8 | 13.7 (a) | 8.2, 28.3 | 116.8 (ab) | 0.3, 459.1 | 7.2 (b) | 5.2, 11.6 | 7.1 (b) | 5.1, 11.0 |

*Overall (%) (/18) includes all micronutrients except Sodium, Chloride and Vitamin E. Overall (%) (/20) includes all micronutrients except Vitamin E. Unlike letters indicate significantly different P < 0.05. Pairwise comparisons conducted using Kruskal–Wallis ANOVA with Bonferroni correction for multiple comparisons. Items were classified into ‘healthy’ or ‘unhealthy’ based on the presence or absence of a red FOPL traffic light for fat, saturated fat, total sugar or salt. This is based on research that when identifying healthier products, UK consumers are more cautious to avoid items with red traffic lights, than to select items with green traffic lights. Selenium: total = 1824 (UPF, n = 841). MPF: minimally processed food;; PCI: processed culinary ingredients; PF: processed food; UPF: ultra-processed food.*

**Table 17. Average percentage contribution of unhealthy items per 100g to UK government dietary micronutrient recommendations from the UK NDNS for males aged 19-64 years, and across each Nova group.**

| **Micronutrient** | **Total (n = 1134)** | | **MPF (n = 166)** | | **PCI (n = 52)** | | **PF (n = 121)** | | **UPF (n = 795)** | |
| --- | --- | --- | --- | --- | --- | --- | --- | --- | --- | --- |
|  | **Median** | **IQR** | **Median** | **IQR** | **Median** | **IQR** | **Median** | **IQR** | **Median** | **IQR** |
| Vitamin A (retinol equivalents) (%) | 3.0 | 0.0, 15.7 | 2.6 (a) | 0.1, 16.9 | 0.0 (a) | 0.0, 45.6 | 17.4 (b) | 3.1, 36.5 | 2.3 (a) | 0.0, 11.3 |
| Vitamin D (%) | 0.0 | 0.0, 4.6 | 0.0 (a) | 0.0, 4.1 | 0.0 (a) | 0.0, 1.0 | 3.0 (b) | 0.4, 8.7 | 0.0 (a) | 0.0, 4.0 |
| Thiamin (%) | 9.0 | 3.0, 18.0 | 16.5 (a) | 8.0, 34.0 | 0.0 (b) | 0.0, 0.8 | 8.0 (c) | 3.0, 13.0 | 8.0 (c) | 3.0, 17.0 |
| Riboflavin (%) | 10.0 | 3.8, 16.9 | 13.8 (a) | 6.9, 22.3 | 0.0 (b) | 0.0, 12.9 | 15.4 (a) | 6.9, 23.8 | 9.2 (c) | 3.1, 14.6 |
| Niacin equivalent (%) | 13.5 | 5.3, 32.4 | 22.9 (a) | 7.5, 54.3 | 0.0 (b) | 0.0, 2.2 | 25.3 (a) | 12.9, 40.6 | 12.4 (c) | 5.3, 26.5 |
| Vitamin C (%) | 0.0 | 0.0, 2.0 | 0.0 (a) | 0.0, 7.8 | 0.0 (b) | 0.0, 0.0 | 0.0 (b) | 0.0, 0.9 | 0.0 (b) | 0.0, 2.0 |
| Vitamin E (%) | 22.5 | 5.0, 62.5 | 33.8 (a) | 7.5, 83.1 | 18.8 (a) | 0.0, 140.6 | 15.0 (a) | 7.5, 50.0 | 22.5 (a) | 5.0, 55.0 |
| Vitamin B6 (%) | 7.1 | 0.0, 14.3 | 21.4 (a) | 7.1, 28.6 | 0.0 (b) | 0.0, 0.0 | 7.1 (c) | 7.1, 14.3 | 7.1 (c) | 0.0, 14.3 |
| Vitamin B12 (%) | 6.7 | 0.0, 46.7 | 0.0 (a) | 0.0, 80.0 | 0.0 (b) | 0.0, 13.3 | 40.0 (c) | 6.7, 113.3 | 6.7 (a) | 0.0, 33.3 |
| Folate (%) | 4.5 | 1.5, 10.0 | 6.3 (a) | 2.0, 25.1 | 0.0 (b) | 0.0, 0.0 | 7.5 (a) | 4.0, 15.5 | 4.0 (c) | 1.5, 8.0 |
|  |  |  |  |  |  |  |  |  |  |  |
| Potassium (%) | 5.6 | 3.1, 9.7 | 11.9 (a) | 7.5, 23.5 | 0.8 (b) | 0.0, 2.9 | 4.3 (c) | 2.8, 8.3 | 5.4 (c) | 3.2, 8.7 |
| Calcium (%) | 8.6 | 2.6, 17.1 | 8.3 (a) | 2.4, 21.1 | 0.7 (b) | 0.0, 9.6 | 12.1 (c) | 5.7, 44.3 | 8.4 (a) | 2.4, 15.7 |
| Magnesium (%) | 7.0 | 3.7, 13.7 | 10.0 (a) | 5.7, 44.5 | 0.5 (b) | 0.0, 2.9 | 6.7 (c) | 5.0, 11.0 | 7.0 (c) | 3.7, 12.7 |
| Phosphorus (%) | 23.6 | 11.1, 38.4 | 34.0 (a) | 16.1, 54.5 | 0.5 (b) | 0.0, 10.3 | 34.7 (a) | 20.5, 60.0 | 22.4 (c) | 10.7, 36.0 |
| Iron (%) | 12.6 | 4.6, 23.0 | 21.8 (a) | 10.3, 44.8 | 1.1 (b) | 0.0, 4.3 | 9.2 (c) | 3.4, 16.1 | 12.6 (d) | 5.7, 20.7 |
| Copper (%) | 9.2 | 4.2, 20.8 | 13.3 (a) | 5.0, 50.4 | 0.8 (b) | 0.0, 3.5 | 7.5 (c) | 4.2, 15.8 | 10.0 (c) | 5.0, 19.2 |
| Zinc (%) | 7.4 | 3.2, 14.7 | 16.8 (a) | 4.2, 36.1 | 1.1 (b) | 0.0, 2.9 | 11.6 (a) | 6.8, 30.5 | 7.4 (c) | 3.2, 12.6 |
| Sodium (%) | 13.7 | 3.9, 34.0 | 2.8 (a) | 0.9, 9.3 | 0.7 (a) | 0.0, 2.5 | 24.2 (b) | 12.0, 43.1 | 16.3 (b) | 5.7, 36.3 |
| Chloride (%) | 11.7 | 3.6, 33.6 | 3.2 (a) | 1.2, 10.6 | 0.7 (b) | 0.0, 3.2 | 22.3 (c) | 10.7, 41.6 | 13.4 (d) | 5.0, 35.1 |
| Iodine (%) | 5.7 | 1.4, 12.9 | 5.4 (a) | 1.4, 14.3 | 0.0 (b) | 0.0, 20.7 | 9.3 (c) | 5.7, 21.4 | 5.7 (a) | 2.1, 11.4 |
| Selenium (%) | 4.0 | 1.3, 8 | 5.3 (a) | 1.3, 17.3 | 0.0 (b) | 0.0, 0.0 | 6.7 (a) | 4.0, 10.0 | 4.0 (c) | 1.3, 8.0 |
|  |  |  |  |  |  |  |  |  |  |  |
| Overall (%) (/18, Exc VitE, NA, CL) (n = 1133) | 12.4 | 7.5, 20.7 | 23.5 (a) | 12.5, 42.8 | 3.2 (b) | 0.1, 9.0 | 17.6 (a) | 10.4, 28.2 | 11.4 (c) | 7.1, 16.6 |
| Overall (%) (/20, Inc NA, CL, Exc VItE) (n = 1133) | 13.4 | 8.3, 22.9 | 22.8 (a) | 12.7, 40.9 | 5.1 (b) | 0.2, 10.7 | 18.2 (a) | 10.8, 28.3 | 12.0 (c) | 7.9, 20.0 |

*Overall (%) (/18) includes all micronutrients except Sodium, Chloride and Vitamin E. Overall (%) (/20) includes all micronutrients except Vitamin E. Unlike letters indicate significantly different P < 0.05. Pairwise comparisons conducted using Kruskal–Wallis ANOVA with Bonferroni correction for multiple comparisons. Items were classified into ‘healthy’ or ‘unhealthy’ based on the presence or absence of a red FOPL traffic light for fat, saturated fat, total sugar or salt. This is based on research that when identifying healthier products, UK consumers are more cautious to avoid items with red traffic lights, than to select items with green traffic lights. Selenium: total = 1133 (UPF, n = 794). IQR: inter-quartile range; MPF: minimally processed food; PCI: processed culinary ingredients; PF: processed food; UPF: ultra-processed food.*

**Table 18. Average percentage contribution of unhealthy items per 100kcal to UK government dietary micronutrient recommendations from the UK NDNS for males aged 19-64 years, and across each Nova group.**

| **Micronutrient** | **Total (n = 1130)** | | **MPF (n = 166)** | | **PCI (n = 48)** | | **PF (n = 121)** | | **UPF (n = 795)** | |
| --- | --- | --- | --- | --- | --- | --- | --- | --- | --- | --- |
|  | **Median** | **IQR** | **Median** | **IQR** | **Median** | **IQR** | **Median** | **IQR** | **Median** | **IQR** |
| Vitamin A (retinol equivalents) (%) | 0.9 | 0.0, 6.0 | 0.9 (a) | 0.0, 8.7 | 0.0 (ac) | 0.0, 19.8 | 6.2 (b) | 1.0, 12.7 | 0.8 (c) | 0.0, 3.8 |
| Vitamin D (%) | 0.0 | 0.0, 1.4 | 0.0 (a) | 0.0, 2.0 | 0.0 (a) | 0.0, 0.5 | 0.8 (b) | 0.1, 2.8 | 0.0 (a) | 0.0, 1.4 |
| Thiamin (%) | 2.9 | 1.0, 6.3 | 5.9 (a) | 3.9, 11.7 | 0.0 (b) | 0.0, 0.4 | 2.7 (c) | 0.9, 4.1 | 2.6 (c) | 1.0, 5.6 |
| Riboflavin (%) | 3.2 | 1.2, 7.2 | 5.5 (a) | 2.5, 9.6 | 0.0 (b) | 0.0, 3.3 | 5.8 (a) | 2.0, 8.7 | 2.8 (c) | 0.9, 6.2 |
| Niacin equivalent (%) | 4.4 | 2.0, 10.5 | 9.2 (a) | 4.7, 18.7 | 0.0 (b) | 0.0, 0.7 | 8.9 (a) | 4.6, 12.9 | 3.3 (c) | 1.7, 8.9 |
| Vitamin C (%) | 0.0 | 0.0, 0.7 | 0.0 (a) | 0.0, 7.6 | 0.0 (b) | 0.0, 0.0 | 0.0 (b) | 0.0, 0.2 | 0.0 (b) | 0.0, 0.6 |
| Vitamin E (%) | 7.5 | 2.7, 18.2 | 12.0 (a) | 3.8, 31.9 | 6.4 (b) | 0.0, 31.7 | 5.2 (b) | 3.1, 16.4 | 7.5 (b) | 2.1, 15.5 |
| Vitamin B6 (%) | 2.3 | 0.0, 6.6 | 7.5 (a) | 5.1, 10.4 | 0.0 (b) | 0.0, 0.0 | 2.6 (c) | 1.7, 5.4 | 1.8 (d) | 0.0, 5.0 |
| Vitamin B12 (%) | 1.7 | 0.0, 17.0 | 0.0 (a) | 0.0, 47.8 | 0.0 (b) | 0.0, 2.7 | 12.5 (c) | 1.9, 38.5 | 1.7 (a) | 0.0, 12.9 |
| Folate (%) | 1.4 | 0.5, 3.9 | 3.6 (a) | 0.9, 7.7 | 0.0 (b) | 0.0, 0.3 | 3.4 (a) | 1.2, 5.3 | 1.2 (c) | 0.5, 2.9 |
|  |  |  |  |  |  |  |  |  |  |  |
| Potassium (%) | 1.9 | 1.0, 3.5 | 4.6 (a) | 3.2, 9.4 | 0.1 (b) | 0.0, 1.2 | 1.6 (c) | 0.9, 3.4 | 1.7 (c) | 1.0, 2.9 |
| Calcium (%) | 2.8 | 1.0, 6.1 | 3.1 (a) | 1.0, 8.2 | 0.2 (b) | 0.0, 3.2 | 4.7 (c) | 1.9, 20.6 | 2.7 (d) | 1.0, 5.1 |
| Magnesium (%) | 2.6 | 1.4, 4.4 | 5.1 (a) | 2.8, 12.5 | 0.1 (b) | 0.0, 1.4 | 2.7 (c) | 1.7, 4.0 | 2.4 (c) | 1.2, 3.9 |
| Phosphorus (%) | 7.5 | 4.1, 13.6 | 13.2 (a) | 7.3, 19.2 | 0.2 (a) | 0.0, 2.8 | 12.9 (a) | 6.4, 21.0 | 6.6 (c) | 3.8, 11.7 |
| Iron (%) | 3.9 | 1.9, 7.2 | 8.1 (a) | 3.9, 20.1 | 0.1 (b) | 0.0, 0.7 | 3.1 (c) | 1.0, 5.4 | 3.8 (d) | 2.1, 6.3 |
| Copper (%) | 3.1 | 1.4, 6.2 | 7.6 (a) | 2.3, 15.0 | 0.1 (b) | 0.0, 0.4 | 2.6 (c) | 1.3, 7.7 | 3.1 (c) | 1.6, 5.6 |
| Zinc (%) | 2.3 | 1.3, 6.1 | 6.4 (a) | 2.8, 10.7 | 0.2 (b) | 0.0, 1.6 | 4.7 (a) | 2.1, 10.2 | 2.0 (c) | 1.1, 4.5 |
| Sodium (%) | 3.9 | 1.3, 10.6 | 1.4 (a) | 0.2, 4.1 | 0.1 (b) | 0.0, 0.9 | 7.2 (c) | 3.5, 12.8 | 4.5 (d) | 1.9, 11.3 |
| Chloride (%) | 3.7 | 1.2, 9.8 | 1.3 (a) | 0.4, 5.0 | 0.1 (b) | 0.0, 1.4 | 6.7 (c) | 3.5, 12.8 | 3.9 (d) | 1.5, 11.0 |
| Iodine (%) | 2.0 | 0.6, 4.5 | 2.0 (a) | 0.8, 6.2 | 0.0 (b) | 0.0, 3.0 | 3.3 (c) | 1.7, 7.4 | 1.9 (a) | 0.5, 4.2 |
| Selenium (%) | 1.2 | 0.4, 2.7 | 2.3 (a) | 0.5, 6.1 | 0.0 (a) | 0.0, 0.0 | 1.9 (a) | 1.0, 3.6 | 1.1 (c) | 0.4, 2.5 |
|  |  |  |  |  |  |  |  |  |  |  |
| Overall (%) (/18, Exc VitE, NA, CL) (n = 1129) | 4.1 | 2.1, 8.2 | 8.9 (a) | 5.8, 14.5 | 0.6 (b) | 0.0, 2.6 | 6.4 (c) | 3.3, 8.7 | 3.3 (d) | 1.9, 6.5 |
| Overall (%) (/20, Inc NA, CL, Exc VItE) (n = 1129) | 4.4 | 2.2, 9.0 | 8.4 (a) | 5.4, 13.8 | 0.6 (b) | 0.0, 2.4 | 6.7 (c) | 3.9, 9.9 | 3.5 (d) | 2.1, 7.8 |

*Overall (%) (/18) includes all micronutrients except Sodium, Chloride and Vitamin E. Overall (%) (/20) includes all micronutrients except Vitamin E. Unlike letters indicate significantly different P < 0.05. Pairwise comparisons conducted using Kruskal–Wallis ANOVA with Bonferroni correction for multiple comparisons. Items were classified into ‘healthy’ or ‘unhealthy’ based on the presence or absence of a red FOPL traffic light for fat, saturated fat, total sugar or salt. This is based on research that when identifying healthier products, UK consumers are more cautious to avoid items with red traffic lights, than to select items with green traffic lights. Selenium: total = 1129 (UPF, n = 794). IQR: inter-quartile range; MPF: minimally processed food; PCI: processed culinary ingredients; PF: processed food; UPF: ultra-processed food.*
